# Plant Genotype Influences Physicochemical Properties of Substrate as Well as Bacterial and Fungal Assemblages in the Rhizosphere of Balsam Poplar

**DOI:** 10.3389/fmicb.2020.575625

**Published:** 2020-11-23

**Authors:** Karelle Rheault, Denis Lachance, Marie-Josée Morency, Évelyne Thiffault, Marie Guittonny, Nathalie Isabel, Christine Martineau, Armand Séguin

**Affiliations:** ^1^Natural Resources Canada, Canadian Forest Service, Laurentian Forestry Centre, Quebec City, QC, Canada; ^2^Renewable Materials Research Centre, Department of Wood and Forest Sciences, Université Laval, Quebec City, QC, Canada; ^3^Research Institute of Mines and Environment (RIME), Université du Québec en Abitibi-Témiscamingue, Rouyn-Noranda, QC, Canada

**Keywords:** *Populus balsamifera*, genotype-by-environment interactions, microbiome, tree genetics, mine waste revegetation, plant–microbe interactions

## Abstract

Abandoned unrestored mines are an important environmental concern as they typically remain unvegetated for decades, exposing vast amounts of mine waste to erosion. Several factors limit the revegetation of these sites, including extreme abiotic and unfavorable biotic conditions. However, some pioneer tree species having high levels of genetic diversity, such as balsam poplar *(Populus balsamifera)*, can naturally colonize these sites and initiate plant succession. This suggests that some tree genotypes are likely more suited for acclimation to the conditions of mine wastes. In this study, we selected two contrasting mine waste storage facilities (waste rock from a gold mine and tailings from a molybdenum mine) from the Abitibi region of Quebec (Canada), on which poplars were found to have grown naturally. First, we assessed *in situ* the impact of vegetation presence on each mine waste type. The presence of balsam poplars improved soil health locally by modifying the physicochemical properties (e.g., higher nutrient content and pH) of the mine wastes and causing an important shift in their bacterial and fungal community compositions, going from lithotrophic communities that dominate mine waste environments to heterotrophic communities involved in nutrient cycling. Next, in a greenhouse experiment we assessed the impact of plant genotype when grown in these mine wastes. Ten genotypes of *P. balsamifera* were collected locally, found growing either at the mine sites or in the surrounding natural forest. Tree growth was monitored over two growing seasons, after which the effects of genotype-by-environment interactions were assessed by measuring the physicochemical properties of the substrates and the changes in microbial community assembly. Although substrate type was identified as the main driver of rhizosphere microbiome diversity and community structure, a significant effect due to tree genotype was also detected, particularly for bacterial communities. Plant genotype also influenced aboveground tree growth and the physicochemical properties of the substrates. These results highlight the influence of balsam poplar genotype on the soil environment and the potential importance of tree genotype selection in the context of mine waste revegetation.

## Introduction

The mining industry has an important environmental impact due to abandoned and unrestored mine sites that can remain unvegetated for decades. Indeed, the nature and composition of mine wastes make them challenging substrates for plant growth: they are nutrient poor, have either a very low or very high pH and have poor physical structure and deficient water-holding capacity (Kumaresan et al., 2017). Abandoned mine sites often constitute a major eyesore to adjacent communities and pose further risk to surrounding ecosystems as vast amounts of soil, waste rock and tailings are exposed to aeolian and water erosion (Mendez and Maier, 2008). Soil microorganisms are in part responsible for the negative impacts of mine sites, mainly through the formation of acid mine drainage, generally at pH lower than five (Bussière et al., 2005). Revegetation could help initiate the restoration of these disturbed ecosystems. Established plants, together with their associated microbiome, have the ability to reduce acid mine drainage and modify the physicochemical properties of their environment, improving soil quality and fertility (Hubbard et al., 2018). Furthermore, plants form a biological cap that reduces soil erosion while increasing water retention and organic matter content in coarse mine wastes, which contributes to soil stability (Tordoff et al., 2000).

Parameters used to assess the success of revegetation are based on plant, soil and microbial criteria. Plant criteria include plant survival and biomass, leaf and shoot metal concentrations, the establishment of other native colonizers and the ability to self-propagate (Tordoff et al., 2000). Soil criteria include improvement in soil structure such as increased soil aggregation and reduced erosion; and improvement in soil physicochemical properties such as less acidic pH, increased organic matter content and reduced metal bioavailability and mobility (Mendez and Maier, 2008; Lauber et al., 2009). Although currently not widely used, microbial criteria include a decrease in autotrophic bacteria followed by an increase in heterotrophic bacteria and fungi (Rosario et al., 2007) and an increase in bacterial diversity and richness (Garbeva et al., 2004). Throughout this paper, the expression “improved soil health” will be used to summarize the above-mentioned soil and microbial criteria.

The efficiency of revegetation largely depends on the establishment of a large root network and beneficial root-soil microbe interactions (Callender et al., 2016). Poplars, with their high level of genetic diversity, could thus be ideal candidates for revegetation purposes. Indeed, poplars are pioneer trees: they rapidly grow in a vast range of environmental conditions and they easily propagate by root suckering and crown breakage (Dickmann and Kuzovkina, 2014). They also have a large and deep root system (Braatne et al., 1996). As a perennial species, they can tolerate harsh environments and promote the establishment of primary and successive plant species through the addition of soil nutrients from their abundant litter production, increasing ecosystem health and function (Pardon et al., 2017). Additionally, they can establish root associations with both arbuscular mycorrhizal and ectomycorrhizal fungi (Gehring et al., 2006), as well as other endophytic and rhizospheric organisms (Bulgarelli et al., 2013). Those associations have the potential to increase access to nutrients, relieve abiotic stresses such as hydric stress, suppress plant pathogens, alter phenology and promote plant growth (Bulgarelli et al., 2013). Finally, *Populus* is considered as a model genus for the study of woody perennials and therefore massive genomic resources are available (e.g., a fully sequenced genome, Tuskan et al., 2006) which provides unique opportunity to support selection of most suited genotypes to address ecosystem restoration issues (Fini et al., 2017).

Many studies have attempted to characterize the root microbiome of *Populus*. It has been shown that soil type is the main driver of microbial community assembly, since physicochemical properties, such as granulometry, pH and nutrient content, influence microbial composition and functional group prevalence (Gottel et al., 2011; Danielsen et al., 2012; Shakya et al., 2013; Cregger et al., 2018). However, genetic variations of the host plant are also associated with differential microbial colonization (Badri et al., 2009; Philippot et al., 2013; Bonito et al., 2014; Lebeis et al., 2015; Veach et al., 2019). Differentiating between the effects of soil properties and those of the host plant genotype has not yet been sufficiently addressed (Bonito et al., 2019).

The aim of this study was to assess the suitability of *Populus balsamifera* for the revegetation of abandoned mine sites and to determine if the selection of specific tree genotypes can improve soil health and the rhizosphere microbiome. To do so, a two-part study was conducted: two mine sites from Abitibi, Quebec, where poplars have naturally grown, were selected to harvest contrasting mine wastes and balsam poplar cuttings. First, in a field study, we assessed the effect of balsam poplar presence on waste rock from a gold mine and tailings from a molybdenum mine on the physicochemical properties of the mine wastes and the composition of their microbiome. Once the field study confirmed our hypothesis that *P. balsamifera* is suitable to improve soil health *in situ*, we investigated the effect of genotype-by-environment interaction in a greenhouse study. Ten genotypes of *P. balsamifera* collected from both mine sites and from a natural forest nearby were grown in mine substrates (waste rock and tailings). Tree growth was monitored over two growing seasons, after which the effect of genotype on physicochemical properties and microbial communities of the substrates were investigated. The main objectives were (1) to assess the effect of vegetation presence on two contrasting mine wastes by measuring physicochemical properties and characterizing bacterial and fungal communities of the mine wastes *in situ*; and (2) to assess the effect of tree genotype on the physicochemical properties of contrasting substrates and the diversity and composition of the rhizosphere microbiome in a greenhouse study.

## Materials and Methods

### Field Site Description and Sampling

Two mine sites, 50 km apart, located in Abitibi (western Quebec, Canada), were chosen for the contrasting characteristics of their mine wastes and for the fact that they both have balsam poplars naturally growing on the periphery, among other plant species such as willows (*Salix*), trembling aspen (*Populus tremuloides)*, alder (*Alnus*), and birch (*Betula*). The Westwood site, formerly the Doyon site, is characterized by its acid generating and coarse waste rock piles; the La Corne Mine site is dominated by neutral and fine-grained tailing piles. Both mine wastes are nutrient poor. The Westwood site is a former gold mine, recently put back into operation and owned by IAMGOLD Corporation. The La Corne Mine site is a former molybdenum and bismuth mine out of operation since 1972 and owned by Romios. See Supplementary Figures S1, S2 for pictures of the sites and Supplementary Figure S3 for a summary of the field and the greenhouse experiments. Mine wastes, vegetated soil samples and tree cuttings were sampled in November 2016.

Mine waste was collected from the tailing stockpiles at the La Corne Mine site as well as from waste rock stockpiles from the former Doyon mine site at the Westwood site. Samples were collected from the top 20 cm of the stockpiles and stored at 4°C until used. Fifteen bulk soil samples of 15 g from five unvegetated areas along with five areas colonized by balsam poplars were collected on each mine site for DNA extraction and physicochemical analyses. Upon returning to the laboratory, subsamples were taken from each 15 g sample, placed in 1.5 mL tubes and stored at −20°C until DNA extraction. The remainder of each sample was air-dried for physicochemical analyses.

### Cuttings Sampling, Genotyping, and Growth

Eight mature balsam poplars per mine site and two from a natural forest near the La Corne Mine site were selected, from which branches were harvested to produce cuttings. Branches were dormant when collected. Harvested branches were kept on ice during transport and stored frozen at −5°C immediately upon arrival to the laboratory, until used. In order to reduce the risk of harvesting the same genotype (clone), trees were sampled at a minimum distance of approximately 200 m. Their unique genotypes were then verified using a 40 SNP array designed to reveal *P. balsamifera* intraspecific variations (Supplementary Table S1), as described by Meirmans et al. (2017). For this, DNA was extracted from bud tissue using a Nucleospin 96 Plant II kit (Macherey-Nagel, Bethlehem, PA, United StatesUSA) following the manufacturer’s protocol for centrifugation processing with the following modification: buffer PL2 was used at the cell lysis step and was heated for 2 h at 65°C instead of 30 min. All samples were sent to the Genome Quebec Innovation Centre at McGill University to be genotyped.

Ten cuttings, each containing three buds, were prepared from the tree branches collected from each selected tree in the field. From November 2016 to January 2017, cuttings were first started using a hydroponic system to better allow for root development, then transferred into pots and grown until the end of April 2017 (see Supplementary Data Sheet 1 for additional details about the tree production process). Trees measured at least 30 cm at the start of the experiment. Genotypes for which a minimum of nine replicates (three replicates per substrate type) remained at the end of the tree production process were kept for the experiment, leaving four genotypes per mine site and two genotypes from the natural forest.

### Greenhouse Experiment

#### Substrate Preparation and Experimental Design

Mine wastes were sieved through a 10 mm sieve before use. A peat mix composed of four parts peat (Agro Mix G6, Fafard), two parts vermiculite (Perlite Canada, Inc.) and one part Turface (calcined clay particles, Turface MVP, Turface Athletics) was also prepared. Using a cement mixer, equal volumes of mine waste and the peat mix were combined. In total three substrate treatments were prepared: (1) a 50:50 mix containing waste rock from the Westwood site and the peat mix (waste rock; WR); (2) a 50:50 mix containing tailings from the La Corne Mine site and the peat mix (tailing; TA); and (3) a control substrate composed only of the peat mix and slow-release fertilizer (9 g/L; 18.6.8, N.P.K; Nutricote Total, Type:100, Chisso Asahi Fertilizer, Co., Ltd.; control; CO).

At the end of April, three trees from each genotype were repotted into 4 L pots with each substrate treatment, randomly distributed in a greenhouse and allowed to grow until budset. The trees were then transferred outside in August so as to harden off naturally. In January they were returned to the greenhouse, cut back to 30–50 cm and allowed to complete a second growth season ending at budset. Trees were watered as required.

#### Tree Growth and Health Measurements

The following parameters were measured to assess tree growth and health during the two growing seasons: increase in height (growth); chlorophyll content of leaves; shoot diameter; dry biomass of shoots and leaves; and blooming. Height was measured from the soil surface to the terminal bud, at the beginning and end of the first growth season. Height was not measured after the second growth season because the trees were cut back at the start of it. Growth was expressed as a percentage of the difference in heights between the beginning of the experiment and the end of the first season of growth as to normalize the measure between all trees:

%Growth=Heightday 91-Heightday 4Heightday 91×100

As an estimation of leaf nitrogen content, the leaf chlorophyll content, or “greenness,” was measured with a spectrophotometer, following the manufacturer’s instructions (SPAD 502 Plus Chlorophyll Meter, Spectrum Technologies, Inc.). Chlorophyll measurements were made around the middle of each growing season, starting on the fifth leaf from the base of the plant and on every third leaf thereafter, avoiding diseased or immature leaves that were not representative of the whole tree. Shoot diameter was measured 20 cm above the initial cutting at the end of the experiment. Dry biomass was the total mass of the shoot and leaves from the second growth season after drying at 50°C for 7 days. Blooming was measured as the number of days before bud break at the start of the second growth season.

### Rhizosphere and Bulk Sampling

Trees were removed from their pots and the shallow roots removed. Fine roots less than 2 mm in diameter were then sampled, avoiding the taproot in the centre of the pots. Bulk soil was obtained by collecting the soil that detached from those roots with gentle shaking. Rhizosphere soil, the soil still attached to the roots after shaking, was then collected by placing the roots into 50 mL Falcon tubes containing 25 mL of sterile PBS (8 g NaCl, 0.2 g KCl, 1.44 g Na_2_HPO_4_, 0.24 g KH_2_PO_4_, pH 7.4, 1 L distilled water). After briefly shaking the tubes, the roots were removed, the tubes centrifuged at 4700 RPM for 10 min at 4°C, and the supernatant discarded. The pellet, or rhizosphere soil, was then collected and placed on sterile filter papers to absorb excess moisture, then stored in 1.5 mL tubes at −20°C until DNA extraction.

### Processing of Samples

#### Physicochemical Analyses of Bulk Soil

Samples of bulk soil, either collected directly on the mine sites or from the greenhouse experiment, were air-dried, sieved at 2 mm, and kept in plastic bags until further processing. One gram of this soil was ground to a fine powder (0.5 mm diameter particles) prior to physicochemical analyses for carbon, nitrogen and sulfur.

Carbon (C), nitrogen (N), and sulfur (S) were quantified using the TruMac CNS analyzer (LECO Corporation, MI, United States) following the manufacturer’s protocol. Water pH and buffer pH were measured using the methods described by the Canadian Society of Soil Science (Gregorich and Carter, 2007) using the Thermo Scientific Orion 2-Star Benchtop pH meter. Extractable phosphorus (P) and exchangeable cations (potassium (K), calcium (Ca), magnesium (Mg), manganese (Mn), iron (Fe), aluminum (Al), and sodium (Na) were extracted with a Mehlich III extraction buffer (Gregorich and Carter, 2007) and analyzed by inductively coupled plasma (ICP) using an optical emission spectrometer (OES; Optima 7300 DV, Perkin Elmer, Waltham, MA, United States).

#### DNA Isolation and Library Preparation

Bulk soil samples from the field experiment and rhizosphere soil samples from the greenhouse experiment were kept for microbiome analyses. Up to 250 mg of these samples were transferred to PowerBead tubes for DNA extraction using the DNeasy PowerSoil DNA Isolation Kit (Qiagen, Valencia, CA, United States), in accordance with the manufacturer’s instructions, except that DNA was eluted in 50 μL instead of 100 μL. DNA was quantified using a Qubit dsDNA HS Assay Kit and Qubit Fluorometer (Thermo Fisher Scientific, Waltham, MA, United States). Library preparation for Illumina sequencing was performed according to the manufacturer’s instructions for user-defined primers (Illumina, 2013)^1^, with the following modifications. Each sample was amplified in triplicate to ensure reproducibility (Schmidt et al., 2013; Kennedy et al., 2014). Bacterial communities were amplified using primers 515 F-Y (5′-GTGYCAGCMGCCGCGGTAA-3′) and 926R (5′- CCGYCAATTYMTTTRAGTTT-3′) targeting the V4–V5 regions of the 16S rRNA gene of bacteria and archaea (Parada et al., 2016; Rivers, 2016). The ITS2 region of the fungal ribosomal DNA was amplified using the primer set ITS9F (5′-GAACGCAGCRAAIIGYGA-3′) and ITS4R (5′-TCCTCCGCTTATTGATATGC-3′; White et al., 1990; Rivers, 2016). Primers contained the required Illumina adaptors at the 5′ end of the primer sequences (5′-TCGTCGGCAGCGTCAGATGTGTATAAGAGACAG-3′ for the forward primer and 5′-GTCTCGTGGGCTCGGAGATGTGTATAAGAGACAG-3′ for the reverse primer). PCR reactions were set up by first mixing 37.5 μL of HotStarTaq Plus Master Mix (QIAGEN, Inc., Germantown, MD, United States), 27 μL RNase-free water, 1.5 μL of each 10 μM primer and 7.5 μL of gDNA at 5 ng/μL. The final volume of 75 μL was then equally distributed in three 96-well plates placed in distinct thermocyclers. Thermal cycling conditions were as follows: initial denaturation at 95°C for 5 min; 40 cycles (for ITS2 amplification; and 35 cycles for 16S amplification) at 94°C for 30 s, 50°C for 30 s, 72°C for 1 min; and a final elongation at 72°C for 10 min. PCR products were pooled and purified using 81 μL of magnetic beads solution (Agencourt AMPure XP), then unique codes were added to each sample using the Nextera XT Index Kit, in accordance with the above-mentioned Illumina’s protocol. Indexed amplicons were purified with magnetic beads, quantified using a Qubit dsDNA BR Assay Kit (Thermo Fisher Scientific, Waltham, MA, United States) and combined at equimolar concentration. Paired-end sequencing (2 × 250 bp) of the pools was carried out on an Illumina MiSeq at the Illumina Sequencing Platform, Nucleic Acids Solutions, Aquatic and Crop Resource Development, National Research Council Canada-Saskatoon. To compensate for low base diversity when sequencing amplicon libraries, PhiX Control v3 Library was denatured and diluted to 12.5 pM before being added to the denatured and diluted amplicon library at 15% v/v. The amplicon libraries were sequenced at a concentration of 6.5 pM for most of the sequencing runs. The Illumina data generated in this study was deposited in the NCBI Sequence Read Archive and is available under the project number PRJNA615167.

### Bioinformatic Analyses

All bioinformatics analyses were performed in QIIME (version 1.9.1; Caporaso et al., 2010). Briefly, sequence reads were merged with their overlapping paired-end (fastq_mergepairs), trimmed to remove primers (fastx_truncate), and filtered for quality (fastq_filter) using USEARCH (Edgar, 2010). Unique identifiers were inserted into the header of the remaining high-quality sequences, and sequences from the different samples were pooled together (add_qiime_labels) prior to further analyses.

UPARSE (Edgar, 2013) was then used to dereplicate the sequences (derep_fulllength), discard singletons (sortbysize), group high quality reads into operational taxonomic units (OTUs) using a 97% identity threshold (cluster_otus; Schloss et al., 2009), and identify chimeras (uchime_ref). The taxonomic assignment of OTUs was done using the QIIME “assign_taxonomy” command with Mothur as the assignment method and Greengenes Database (McDonald et al., 2012) files as the reference for bacteria and the UNITE database (Abarenkov et al., 2010) files for fungi. The “make_OTU_table” command was then used to generate the OTU table in the “biom” format, which was then used by QIIME in the next steps. OTUs from non-bacterial (or non-fungal) taxa were excluded using the “filter_taxa_from_otu_table” command. For the 16S rRNA analysis, sequences corresponding to chloroplasts, mitochondria and to the kingdom *Plantae* were removed; for the ITS2 analysis, sequences assigned to the kingdom *Protozoa*, *Protista*, *Chromista*, and *Plantae* were removed.

Operational taxonomic units with relative abundances below 0.005% were excluded as previously described (Bokulich et al., 2013) prior to diversity analyses using the “filter_otus_from_otu_table” command. Each sample was rarefied to the lowest number of reads observed among libraries from each data set with QIIME’s “single_rarefaction” command, so the rarefied samples all contained the same number of sequences. Finally, the OTU rarefied list file was used in QIIME’s “core_diversity_analyses” and “alpha_diversity” commands to generate alpha diversity measures (chao1 and Shannon indices), calculate the beta diversity between samples (Bray-Curtis dissimilarity), and generate community composition profiles at different taxonomic levels.

Fungal functional groups were predicted using a homemade Python script based on a predetermined list of fungi genus associated with their respective function (Tedersoo et al., 2014). First, the script optimizes the list to detect and remove non-unique entries (these data are compared to each other to determine which data contains the most information). The corrected data is then compared to an excel file, where the genus is the search key: the script analyzes each line of the file to extract the genus of each OTU; this genus is then compared to a reference library to identify the associated biological function. OTUs unidentified to the genus level are assigned unidentified function.

### Statistical Analyses

Statistical analyses were conducted in R version 3.5.3 (Rproject.org) and figures were produced using the “ggplot2” package. Statistical significance was determined at *p* < 0.05 throughout the analyses. Parametric assumptions were verified before analysis: data normality was checked graphically with normal quantile–quantile plots and computationally with the Shapiro–Wilk test of normality using the “shapiro.test” function. Homoscedasticity was verified using both the Bartlett test (“bartlett.test” function) and the Fligner-Killeen test (“fligner.test” function). Data were transformed using square root (“sqrt” function) or Tukey’s Ladder of Powers (“transformTukey” function, “rcompanion” package) when necessary to meet parametric ANOVA assumptions. A generalized least squares model (“nlme” package) with a stepwise selection and Akaike’s Information Criterion (AIC) minimization approach was performed with vegetation presence (vegetated or unvegetated soil) and waste type (waste rock or tailings) as explanatory variables for the field experiment and tree genotype and substrate type (WR, TA, or CO) as explanatory variables for the greenhouse experiment. Genotype origin (La Corne Mine site, Westwood site or natural forest) was also included in these models (“corComSymm” correlation) to account for its overall large influence. Substrate types were weighted (“varIdent,” weights) to reduce variance due to the fact that they highly differed in their physicochemical properties.

Two-way ANOVA (“anova” function) was used to discern how waste type, vegetation presence, substrate type, genotype, and their interactions influenced taxa relative abundances, physicochemical properties of substrates, and alpha diversity indices. When a factor was revealed as a statistically significant predictor, a Tukey HSD *post hoc* pairwise comparison test (“predictmeans” function, adjusted to “tukey,” “predictmeans” package) was performed between all treatments. For the greenhouse experiment, if the interaction between substrate type and genotype was deemed statistically significant, additional analyses were performed for each substrate type separately to better assess the effect of genotype.

Spearman linear correlation analyses were performed using the “corr.test” function (“psych” package) to determine if there were correlations between physicochemical properties of the substrates, tree growth and taxa relative abundances for both field and greenhouse experiments.

Non-Euclidean distances were calculated from Bray Curtis dissimilarity matrices and implemented in a non-metric multidimensional scaling plot (NMDS, “metaMDS” function, “vegan” package) to visualize both bacterial and fungal community compositional differences between vegetation presence and waste type on the field, and balsam poplar genotypes and substrate type in the greenhouse experiment. A permutational multivariate analysis of variance model [PERMANOVA, “adonis2” function, “vegan” package; (Oksanen et al., 2019)] was also implemented to discern the amount of variation attributed to each factor and their interaction (with 999 permutations). Additional multivariate analyses were performed on communities from each substrate type of the greenhouse experiment separately to better assess the effect of genotype. Variance heterogeneity between the *a priori* selected groups was tested with the functions “betadisper” and “permutes,” in the “vegan” package. Clusters between treatments were determined by a multilevel pairwise comparison test (“pairwise.adonis2” function, “pairwiseAdonis” package). Correlations between NMDS axes and physicochemical properties of substrates were determined using the “envfit” function (with 999 permutations, “vegan” package).

One sample (genotype C21 in control substrate) was removed from analyses because its results were considered aberrant. Two samples from the greenhouse study (genotype W13 in tailings and waste rock) failed to amplify and/or get sequenced for ITS and were therefore excluded from further analyses. Additionally, many samples from the field study failed to amplify and/or get sequenced for ITS and 16S, mainly from unvegetated samples due to a low abundance of reads, and were therefore excluded from further analyses.

## Results

### Assessment of Vegetation’s Effect on Mine Wastes Under Field Conditions

#### Soil Physicochemical Properties

Physicochemical analyses of the mine wastes indicated absence of N, low concentration of C and macronutrients such as P and K, and a relatively high concentration of elements like Fe and S in the waste rock from the Westwood site (Table 1). Additionally, the pH of waste rock was highly acidic with values varying between 2.5 and 3.0, whereas in tailings from La Corne Mine site the pH was almost neutral with values between 5.9 and 7.8. For both tailings and waste rock, significantly higher levels of C, N, K, P, Ca, Mg, and Mn were observed when vegetated (Table 1). Vegetation also reduced S and Fe concentrations, increased pH in waste rock, and reduced pH in tailings.

**TABLE 1:** Physicochemical properties of substrates from the field.

	Reference Forest Soil	Westwood			La Corne Mine		
		Waste Rock	Vegetated	*p*-Value	Tailings	Vegetated	*p*-Value
C total (%)	6.218	0.084	0.863	**0.002**	0.042	2.983	**<0.001**
N total (%)	0.253	<0.006	0.021	0.053	<0.006	0.106	**<0.001**
S total (%)	0.045	0.700	0.134	**<0.001**	0.017	0.030	0.091
pH	4.9	2.8	4.7	**<0.001**	6.4	4.5	**<0.001**
P (mg/kg)	9.83	7.02	23.98	**<0.001**	0.68	3.80	**0.004**
K (cmol_*c*_/kg)	0.418	0.011	0.199	**<0.001**	0.053	0.202	**<0.001**
Ca(cmol_*c*_/kg)	7.69	8.96	1.80	0.069	0.43	2.93	**<0.001**
Mg(cmol_*c*_/kg)	1.590	0.564	0.676	0.413	0.272	0.902	**<0.001**
Mn(cmol_*c*_/kg)	0.143	0.011	0.040	**0.024**	0.024	0.073	**0.003**
Fe (cmol_*c*_/kg)	2.00	6.26	2.68	**<0.001**	2.85	3.10	0.550
Na (cmol_*c*_/kg)	0.035	0.022	0.021	0.805	0.028	0.029	0.929
CEC (cmol_*c*_/kg)	13.6	17.0	8.7	0.102	5.3	9.4	**0.003**
BCSR	71.6	46.3	31.3	0.344	14.1	43.2	**<0.001**

*CEC, cation exchange capacity; BCSR, base cation saturation ratio. Significant differences between unvegetated and vegetated mine wastes are highlighted in bold.*

#### Microbial Diversity, Community Structure, and Composition

**FIGURE 1:**
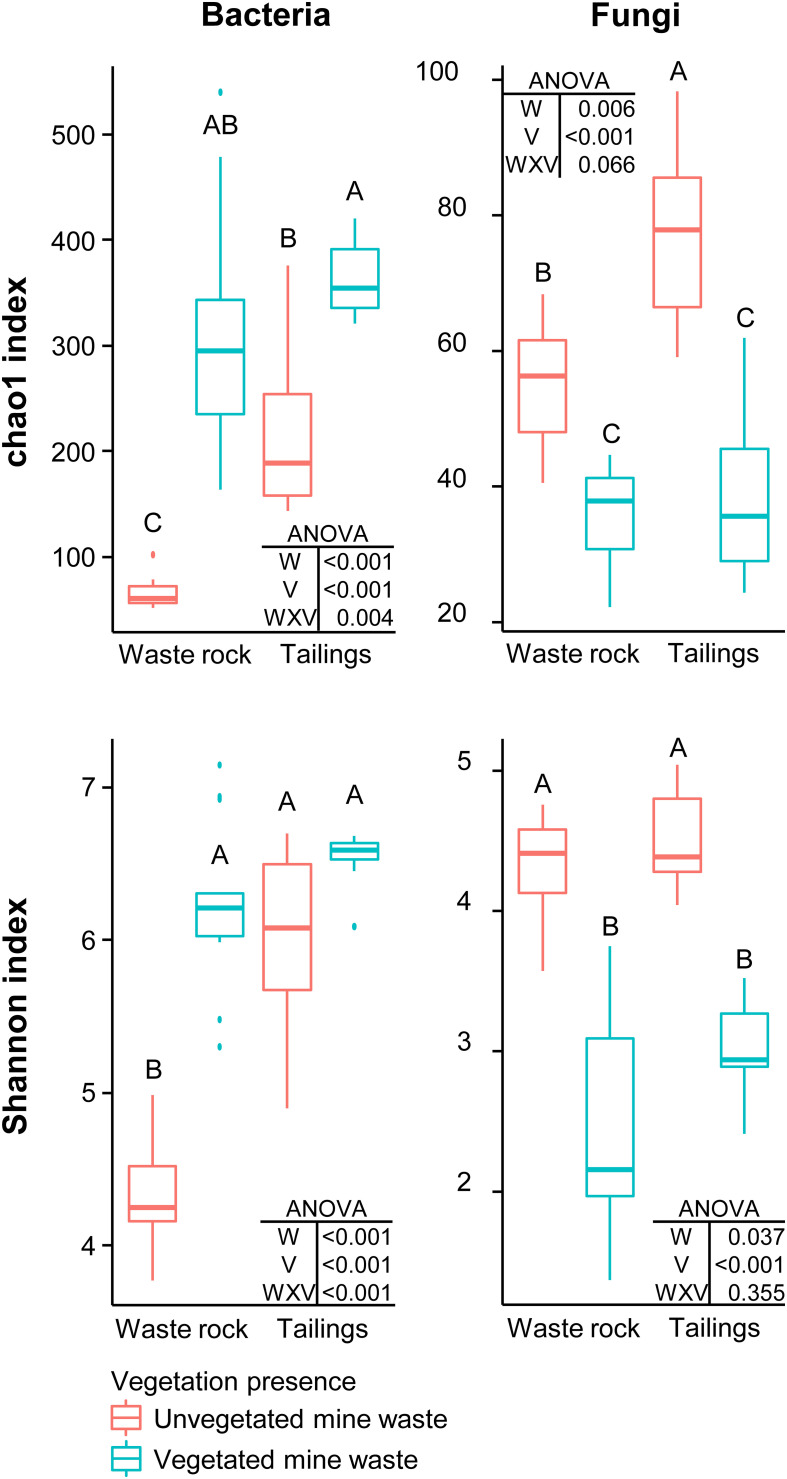
Alpha diversity indices of bacterial and fungal community profiles in field samples. A two-way ANOVA was used to discern how waste type, vegetation presence and their interaction influenced alpha diversity indices. In the ANOVA table, W is waste type; V is vegetation presence; and WXV is the interaction between waste type and vegetation presence. Treatments are composed of two factors: waste rock and tailings as waste type; and vegetated or unvegetated mine waste as vegetation presence. Shared letters between treatments means there is no significant difference between these treatments, as determined by Tukey HSD *post hoc* pairwise comparison test (*n* ≥ 8). Significance level is *p* < 0.05.

Factorial analyses of alpha diversity indices (Figure 1) indicated that vegetation presence (vegetated vs. unvegetated soil), waste type (tailings vs. waste rock) and the interaction between both factors had a consistently significant effect on bacterial richness (chao1 index: *p* < 0.005) and diversity (Shannon index: *p* < 0.001). For fungal richness and diversity, the interaction between factors was not significant (*p* = 0.066 and 0.355). Pairwise comparison of alpha diversity indices indicated that vegetation presence significantly (*p* < 0.05) increased bacterial richness in both waste types and bacterial diversity in waste rock; and reduced fungal richness and diversity in both waste types.

Analysis of beta diversity (Supplementary Figure S4) indicates that bacterial and fungal community structures highly differed between waste types and vegetation presence. The model explains 62% and 35% of the variation in bacterial and fungal community structure, respectively. The main driver of bacterial and fungal community structure was vegetation presence (*R*^2^ = 31.4% and 18.9%, respectively). Waste type also had a significant effect on community structure (*R*^2^ = 14.6 and 9.7%). The interaction between these two factors being significant (*R*^2^ = 16.0 and 6.7%), a multilevel pairwise comparison test was performed on all combinations of waste type by vegetation presence. All combinations clustered separately, indicating that bacterial and fungal community structures differed between each treatment.

**FIGURE 2:**
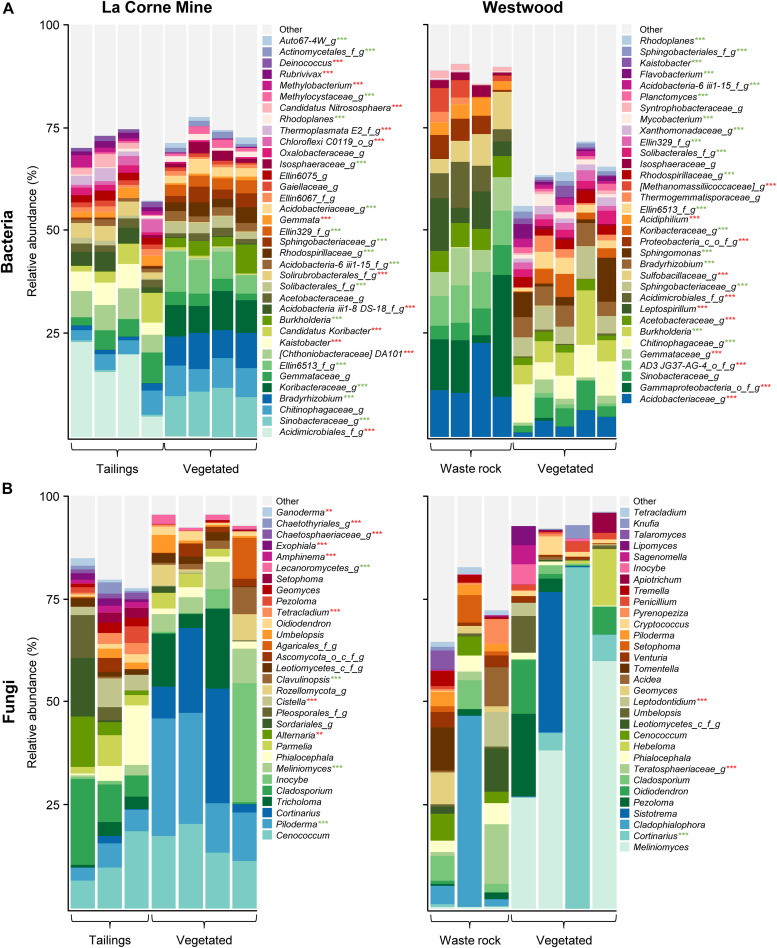
Taxonomic profiles of bacterial and fungal communities in vegetated and unvegetated samples of waste rock from the Westwood site and tailings from the La Corne Mine site. Taxonomic profiles of bacterial communities **(A)** and fungal communities **(B)** at the genus level. Only bacterial and fungal taxa with a relative abundance > 1% in at least one treatment are shown. Green and red stars represent a significant increase and decrease, respectively, in the relative abundance of the taxa in the vegetated soil compared to the unvegetated soil as determined by a Tukey HSD *post hoc* pairwise comparison test (*n* ≥ 3). Significance level is represented as follows: *p* < 0.05 *, *p* < 0.01 **, *p* < 0.001 ***. Pseudo-replications of each location were pooled to simplify visualization.

Taxonomic profiling of bacterial and fungal communities revealed high heterogeneity as shown by the variability among field replicates. Many bacterial and fungal taxa were only identified at a high taxonomic level. Figure 2 illustrates the relative abundance of the most abundant taxa (> 1%, at the genus level) in vegetated soil and mine wastes, at the La Corne Mine site and the Westwood site. Factorial analyses of the relative abundances of bacterial taxa (for taxa > 1%, Supplementary Table S2) showed that waste type had a significant effect on 32 taxa, vegetation presence had a significant effect on 44 taxa, and a significant interaction between both factors was detected for 40 taxa. Pairwise comparisons between all treatments revealed that 50 of the 51 most abundant bacterial taxa (> 1%, at the genus level) had a significant difference in at least one treatment (*p* < 0.001). In fungal communities (Supplementary Table S2), waste type had a significant effect on 11 taxa, vegetation presence had a significant effect on 21 taxa and a significant interaction between both factors was detected for 11 taxa. Pairwise comparison between all treatments showed a significant difference in at least one treatment for 21 of the 47 fungal taxa (*p* < 0.01).

The presence of vegetation on mine wastes reduced the relative abundance of microorganisms associated with acid mine drainage like *Acidiphilium*, *Leptospirillum*, and *Sulfobacillaceae* on waste rock (Figure 2). Additionally, it also reduced the relative abundance of fungal plant pathogens like *Alternaria* and *Ganoderma* on tailings and *Teratosphaeriaceae* on waste rock. Conversely, the presence of vegetation increased the relative abundance of many microorganisms previously found in rhizosphere samples and associated with beneficial ecological functions like the ectomycorrhiza *Meliniomyces* on tailings and the rhizobacteria *Burkholderia* and *Rhodoplanes* on both mine wastes.

Functions associated with fungal communities in all samples comprised ectomycorrhizae (35%), saprotrophs (27%), ericoid mycorrhizae (13%), plant pathogens (3%), white rot (1%) and lichenized (1.8%) and arbuscular (0.05%) mycorrhizae. Factorial analyses of the relative abundances of fungal functions (Supplementary Table S2) showed that there was no effect of waste type nor interaction between waste type and vegetation presence on functional group prevalence. Pairwise comparisons between all treatments revealed that there was no effect of vegetation on the relative abundance of fungal functions in waste rock. However, the presence of vegetation on tailings significantly increased (*p* < 0.001) the relative abundance of ectomycorrhizae and ericoid mycorrhizae; and significantly decreased the relative abundance of saprotrophs (*p* < 0.001), plant pathogens (*p* < 0.001) and arbuscular mycorrhizae (*p* = 0.003).

The relative abundance of all bacterial and fungal taxa was significantly correlated with at least one physicochemical property of the substrates (Supplementary Table S3). Bacterial genera like *Leptospirillum* and *Acidiphilium*, two bacterial genera associated with the oxidoreduction of iron and acid mine drainage were strongly correlated with iron content and the reduction of pH. Similarly, *Sulfobacillaceae*, a bacterial family associated with the oxidation of sulfur and acid mine drainage was strongly correlated with sulfur content and the reduction of pH.

### Assessment of Tree Genotype Effect Under Greenhouse Experiment

#### Tree Growth

**FIGURE 3:**
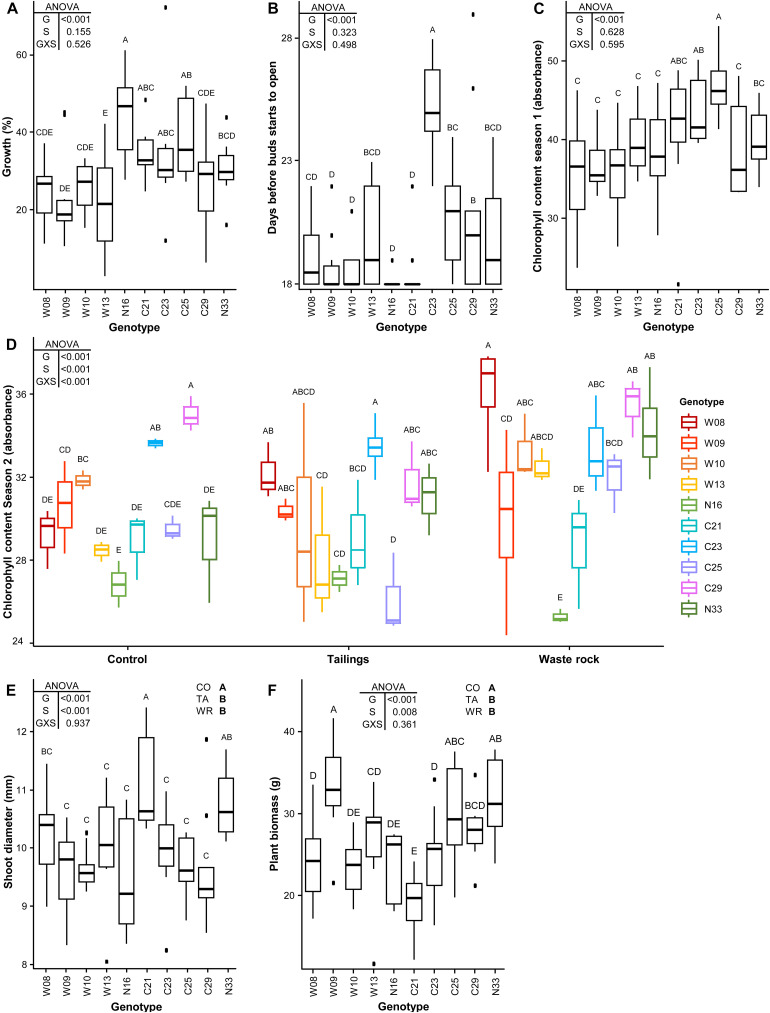
Effect of genotype and substrate type on tree growth measurements. Tree growth during the first season **(A)**; chlorophyll content at the end of the first **(B)** and second **(C)** seasons; blooming during the second season **(D)**; shoot diameter **(E)**; and biomass produced during the second season **(F)**. A two-way ANOVA was used to discern how genotype, substrate type and their interaction influenced tree growth measurements. Shared letters between treatments means there is no significant difference between these treatments as determined by Tukey HSD *post hoc* pairwise comparison test (*n* ≥ 3). In the ANOVA tables, G is genotype; S is substrate type; and GXS is the interaction between genotype and substrate type. In **(E)** and **(F)** CO is control; TA is tailings; and WR is waste rock. Significance level is *p* < 0.05. In **(A–C)**, because only the effect of genotype is significant, measures from all substrate types were pooled. In **(D)**, because the interaction of both factors is significant, all treatments were shown separately. In **(E)** and **(F)**, pooled measures from all substrate types are shown for each genotype as a boxplot and the effect for each substrate type is shown in the upper right corner of the plots.

A significant effect on tree growth measurements was detected for all parameters for genotype, on three parameters for substrate type and on only one parameter for an interaction between the two factors (Figure 3). Tree growth (Figure 3A), the number of days before bud flush at the beginning of the second season (Figure 3B) and chlorophyll content during the first season (Figure 3C) differed among plant genotypes (*p* < 0.001) but not between substrate types (*p* = 0.155, 0.323 and 0.628 respectively); the interaction between both factors was not significant for these parameters (*p* = 0.526, 0.498, and 0.595). Shoot diameter (Figure 3E) and biomass produced during the second season (Figure 3F) differed among plant genotypes (*p* < 0.001) as well as between substrate types (*p* < 0.001 and 0.008 respectively), but again no significant interaction was detected (*p* = 0.937 and 0.361). Shoot diameter and plant biomass measurements were greater in the nutrient-rich control substrate versus mine substrates. Chlorophyll content during the second season (Figure 3D) differed between genotypes and it was greater in the waste rock for some genotypes (significant interaction between genotype and substrate type, *p* < 0.001). The ranking of genotypes strongly differed depending on the measured growth parameter (Figure 3). As an example, genotypes W08, W10, W13, and C29 had lower values for most parameters except for their chlorophyll content after the second season of growth in mine substrates, for which they had the highest values. Genotypes W09 and N16 had the highest biomass and highest growth, respectively, but had lower values for other parameters. Genotype N33 had intermediate to high values for all parameters.

Apart from the effect of genotype, there also was an overall effect for cutting origin (either the Westwood site: W; the La Corne Mine site: C; or the Natural forest: N) on measured growth parameters when the effect of substrate type was not significant: W genotypes grew significantly less than C and N genotypes (Supplementary Figure S5A, *p* < 0.001); C genotypes flushed later than W and N genotypes (Supplementary Figure S5B, *p* < 0.001); and C genotypes had a higher chlorophyll content during the first season than W genotypes (Supplementary Figure S5C, *p* < 0.001). An indication of selection pressure was not observed between cuttings regarding their growth in mine substrates. Indeed, when the effect of substrate type was significant, there was no obvious association between cutting origin and their growth in the substrate from which they were collected. For example, the genotypes that had a higher chlorophyll content in tailings were not only those originating from the La Corne Mine site (Figure 3D). For shoot diameter (Figure 3E) and plant biomass (Figure 3F), there was no significant difference between mine substrates.

#### Physicochemical Properties of Substrates

A factorial analysis of the impact of treatments on physicochemical properties of substrates revealed that all parameters were significantly affected by substrate type, 11 of the 13 parameters had a significant effect of genotype, and the interaction between substrate type and genotype was significant for 10 parameters (Supplementary Table S4). To better assess the effect of genotype, pairwise comparisons were made on each substrate type separately (Table 2 and Supplementary Table S5). All physicochemical properties were significantly affected by genotype in at least one substrate. Although significant, the differences between genotypes were small, generally resulting in only two genotypes being different from the others. Genotypes having a significant effect on physicochemical properties varied depending on the substrate type (Table 2). For example, genotypes associated with higher carbon content were W13 and C29 in the control substrate; C21 and C25 in tailings; and W13 in waste rock. However, trends were observed for some genotypes (Table 2). In all substrates, genotypes W08 and N16 were associated with less favorable soil conditions (lower nutrient and higher sulfur content). In waste rock, genotype C21 led to the lowest content of all elements and the lowest pH, but, in tailings, it was associated with higher nutrient content. In both mine substrates, genotype C29 was associated with higher nutrient content and pH.

**TABLE 2:** Physicochemical properties of substrates after the greenhouse experiment.

	C Total (%)			N Total (/oooo)			P (mg/kg)		
	Control	Tailings	Waste rock	Control	Tailings	Waste rock	Control	Tailings	Waste rock
W08	9.43ab	1.47b	2.50abc	17.8ab	0.35c	3.09bcd	15.7b	2.43	6.2b
W09	8.23ab	1.63ab	2.56ab	17.1ab	0.52c	3.98abc	30.9ab	4.12	10.9a
W10	6.08b	1.93ab	2.55abc	11.7b	1.16bc	3.19bcd	38.0a	6.11	12.6a
W13	10.96a	1.83ab	3.21A	22.2a	0.98bc	4.87ab	34.6ab	7.10	10.9a
N16	10.24ab	2.21ab	2.25bc	21.8ab	1.87abc	2.74cd	27.6ab	5.07	11.1a
C21	10.02ab	2.19a	1.79C	19.3ab	1.91abc	1.32d	33.0ab	5.1	49.2ab
C23	7.60ab	2.05ab	2.61abc	13.9ab	1.22bc	3.50abcd	37.9a	6.06	11.5a
C25	7.81ab	2.14a	2.21bc	15.6ab	1.47bc	4.39abc	35.2a	3.96	11.9a
C29	10.59a	1.95ab	2.71ab	21.1a	3.22a	5.39a	37.3a	7.07	11.9a
N33	7.16ab	1.59ab	2.22bc	14.3ab	2.50ab	4.29abc	29.8ab	2.68	10.5a
*p*-Value	0.009	0.006	0.002	0.009	<0.001	<0.001	0.017	0.027	< 0.001
	**K (cmol_*c*_/kg)**			**Ca (cmol_*c*_/kg)**			**Mg (cmol_*c*_/kg)**		
	**Control**	**Tailings**	**Waste rock**	**Control**	**Tailings**	**Waste rock**	**Control**	**Tailings**	**Waste rock**

W08	4.42cd	1.14b	2.16	9.20c	1.40b	3.56c	3.97	0.64b	0.86ab
W09	9.61abcd	1.60ab	2.20	24.80a	2.85ab	5.93ab	7.07	1.12a	1.28a
W10	11.06a	2.59ab	2.00	21.50abc	3.45a	5.46abc	6.43	1.45a	1.12ab
W13	8.75bc	1.88ab	2.00	23.90ab	3.49a	6.47ab	7.26	1.30a	1.28a
N16	7.56d	2.22ab	1.67	20.00abc	4.66a	4.66abc	6.49	1.62a	1.18ab
C21	9.07abcd	2.67a	1.37	19.50abc	4.10a	3.10c	6.13	1.45a	0.67b
C23	10.11ab	1.98ab	1.72	17.60bc	3.35a	5.99ab	5.70	1.22a	1.24a
C25	9.35abcd	2.84a	1.58	20.90abc	3.59a	4.27bc	6.40	1.46a	1.01ab
C29	7.10d	1.67ab	2.05	19.70abc	4.06a	6.74a	5.95	1.22a	1.28a
N33	8.61abcd	1.83ab	1.57	21.10abc	3.19a	4.88abc	5.77	1.24a	1.06ab
*p*-value	0.013	0.013	0.068	0.005	<0.001	< 0.001	0.094	<0.001	0.003
	**pH**			**S total (‰)**			**Fe (cmol_*c*_/kg)**		
	**Control**	**Tailings**	**Waste rock**	**Control**	**Tailings**	**Waste rock**	**Control**	**Tailings**	**Waste rock**

W08	5.81	5.83	4.66Ab	1.04ab	0.52	10.60a	0.450	0.910D	2.100
W09	5.59	5.51	4.86A	1.27ab	0.54	10.30a	1.054	1.800Ab	2.320
W10	5.68	5.58	4.73Ab	0.62ab	0.70	12.70a	0.869	1.890A	2.450
W13	5.77	5.64	4.92A	1.84a	0.14	10.40a	0.957	1.720abc	2.170
N16	5.64	6.03	4.84A	1.21ab	0.05	10.20a	0.813	1.220Cd	2.130
C21	5.39	5.61	4.32B	1.03ab	0.89	11.20a	0.922	1.420bc	2.660
C23	5.63	5.64	4.83A	1.19ab	0.48	3.40b	1.089	1.580abc	2.700
C25	5.65	5.48	4.66Ab	0.30b	0.11	3.50b	1.028	1.430abc	2.140
C29	5.54	5.62	4.92A	0.34b	0.02	2.60b	0.973	1.650abc	2.270
N33	5.44	5.53	4.75Ab	0.03b	0.03	4.10b	0.998	1.780ab	2.260
*p*-Value	0.282	0.111	0.015	0.011	0.684	<0.001	0.354	< 0.001	0.497

*See Supplementary Table S5 for the other parameters.*

There was an overall effect for the origin of the cuttings on the physicochemical properties of the substrates. Indeed, several elements showed higher concentrations when genotypes were grown in their original mine waste: in tailings, C (*p* = 0.003), N (*p* < 0.001), Ca (*p* = 0.019), and Mn (*p* = 0.029) contents were higher for genotypes originating from the La Corne Mine site; in waste rock, C (*p* = 0.022), N (*p* < 0.001), and K (*p* = 0.002) contents were higher for genotypes originating from the Westwood site. Lastly, plant biomass was significantly, albeit weakly, correlated with N and Na contents of the substrates (Supplementary Table S6).

#### Microbial Diversity, Community Structure, and Composition

**FIGURE 4:**
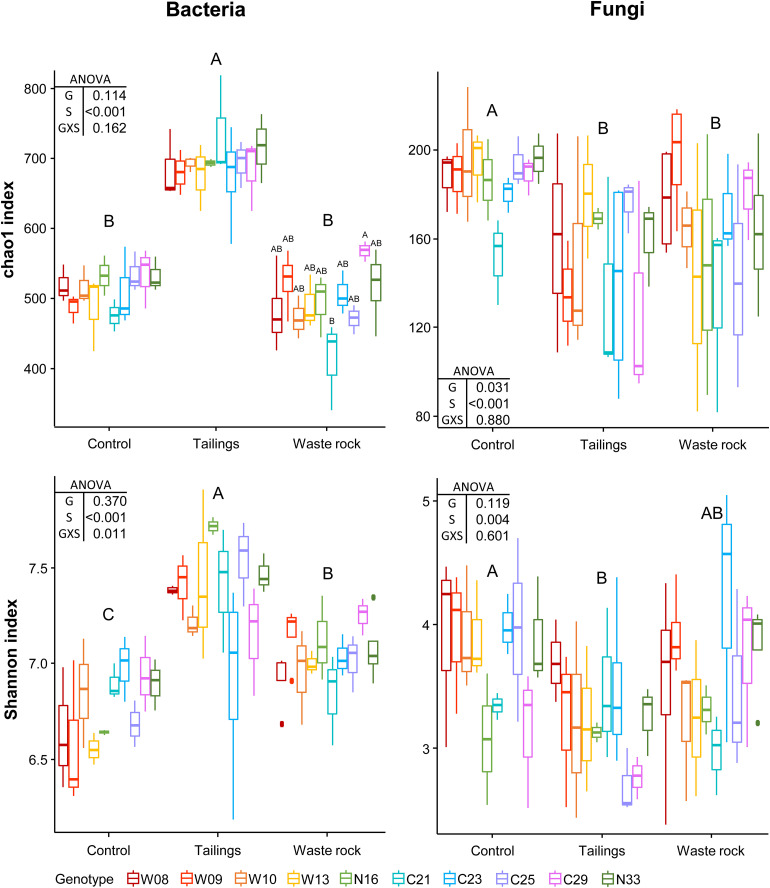
Alpha diversity indices of bacterial and fungal community profiles in greenhouse samples. A two-way ANOVA was used to discern how genotype, substrate type and their interaction influenced alpha diversity indices. In the ANOVA table, G is genotype; S is substrate type; and GXS is the interaction between genotype and substrate type. Shared letters between treatments means there is no significant difference between these treatments, as determined by Tukey HSD *post hoc* pairwise comparison test (*n* ≥ 3). Significance level is *p* < 0.05. Genotype had a significant effect on bacterial richness in waste rock only (chao1 index; *p* = 0.040).

Factorial analyses of alpha diversity indices indicated that substrate type had a significant effect on bacterial and fungal richness (chao1: *p* < 0.001) and diversity (Shannon: *p* < 0.001 and 0.004; Figure 4). There was a significant interaction between substrate type and genotype on bacterial diversity (*p* = 0.031). Pairwise comparison between substrate types indicated that bacterial richness (*p* < 0.001) and diversity (*p* < 0.001) were significantly higher in tailings compared to waste rock and control substrate, but that fungal richness (*p* < 0.001) and diversity (*p* = 0.001) were higher in the control substrate than in mine substrates. Alpha diversity was analyzed by substrate type to better assess the effect of genotype. Bacterial richness was higher in genotype C29 compared to genotype C21 in the waste rock (*p* = 0.040; chao1 index, Figure 4). There was no effect of genotype on fungal alpha diversity.

**FIGURE 5:**
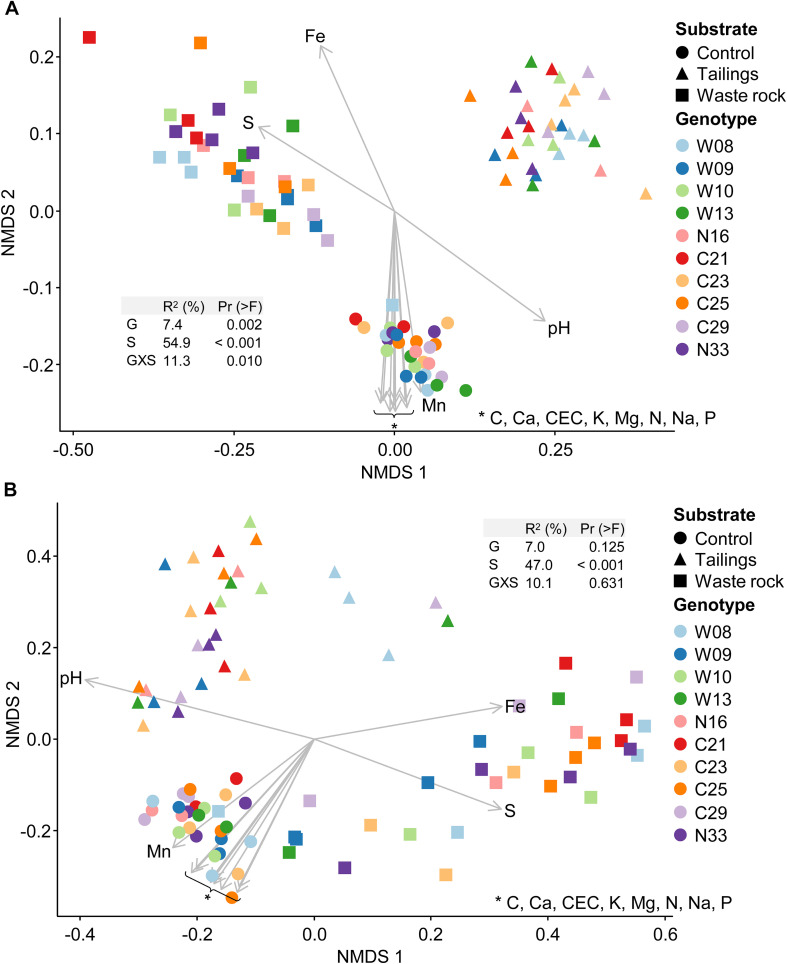
Non-metric multidimensional scaling (NMDS) ordination of variation in bacterial and fungal community structure of balsam poplar rhizosphere in the greenhouse experiment. Bacterial community **(A)** and fungal community **(B)**. Points represent samples and arrows represent the significant (*p* < 0.001) correlations between NMDS axes and the physicochemical properties of the substrates. In PERMANOVA tables, G is for genotype; S for substrate type and GXS for the interaction between genotype and substrate type. The model explains 73.6 and 64.1% of the variation in bacterial and fungal community structure, respectively (*n* ≥ 3).

Bacterial and fungal community structure highly differed between substrate types as shown by variation in beta diversity (Figure 5). The main driver of bacterial and fungal community structure was substrate type (*R*^2^ = 54.9% and 47.0%, respectively), and a multilevel pairwise comparison test revealed that all substrate types, for bacterial and fungal communities, clustered separately (*p* < 0.001). In bacterial beta diversity, genotype and the interaction between substrate type and genotype, explained, respectively, 7.4 and 11.3% of the variation in rhizosphere community structure. Effect of genotype and the interaction were not significant for the fungal community structure. All physicochemical properties of the substrates were significantly (*p* < 0.001) correlated with bacterial and fungal community structures as shown by the arrows on Figure 5.

Beta diversity was analyzed by substrate type separately to better assess the effect of genotype on bacterial and fungal community structure (Supplementary Figure S6). There were differences in bacterial community structure between a few genotypes in both mine substrates (Supplementary Figures S6B,C) and in tailings for fungal community structure (Supplementary Figure S6E). In all substrates, bacterial and fungal community structure was correlated with at least one physicochemical property (arrows in Supplementary Figure S6).

**FIGURE 6:**
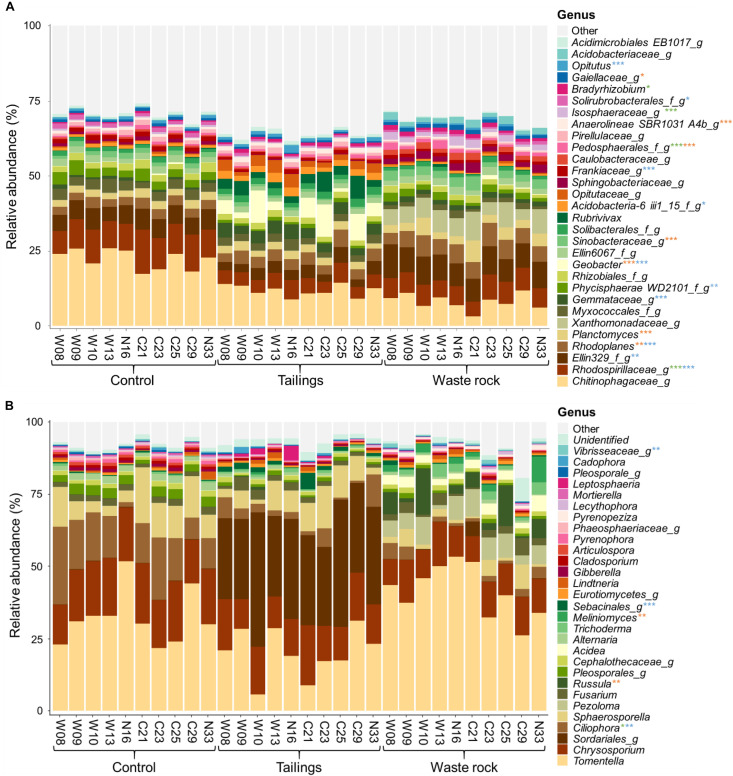
Taxonomic profiles of bacterial and fungal communities in treatments from the greenhouse experiment. Bacterial **(A)** and fungal **(B)** communities at the genus level. Only bacterial and fungal taxa with a relative abundance > 1% in at least one treatment are shown. Replicates for each genotype were pooled for visual simplification (*n* ≥ 3). Stars represent levels of significant difference between genotypes in the three substrates analyzed separately: green for control, blue for tailings and orange for waste rock. Significant differences were determined by a Tukey HSD *post hoc* pairwise comparison test. Significance level is represented as follows: *p* < 0.001 ***, *p* < 0.01 **, *p* < 0.05 *.

Figure 6 illustrates the relative abundance of the most abundant bacterial and fungal taxa (> 1%, at the genus level) in the rhizosphere of balsam poplars after two seasons of growth in tailings, waste rock and control substrates. Many bacterial and fungal taxa were only identified at a high taxonomic level. Functions associated with fungal communities of the rhizosphere comprised mostly ectomycorrhizae (40%), saprotrophs (22%), plant pathogens (5%), ericoid mycorrhizae (1%), and brown rot (1%). Factorial analyses of the relative abundance of each function (Supplementary Table S7) revealed that substrate type had a significant effect on the relative abundance of ectomycorrhizae, saprotrophs, plant pathogens, ericoid mycorrhizae and white rot. Pairwise comparisons between genotypes among each substrate type were performed to better assess the effect of genotype. The effect of genotype was significant on the relative abundance of ericoid mycorrhizae in waste rock: they were more abundant in the rhizosphere of genotype N33 compared to genotypes W09, C25, and C29 (*p* = 0.005).

Factorial analyses of taxa abundance in bacterial and fungal communities (for taxa > 1%, Supplementary Table S8) showed that the effect of substrate type was significant for all bacterial taxa and most fungal taxa (24/29); the effect of genotype was significant for many bacterial and fungal taxa (17/30 and 7/29, respectively); and the interaction between substrate type and genotype was significant for a few bacterial and fungal taxa (13/30 and 5/29, respectively). Pairwise comparisons between genotypes among each substrate type were made to better assess the effect of genotype. The effect of genotype was significant in at least one substrate for half of bacterial taxa (17/30) and for a few fungal taxa (5/29). The bacterial and fungal taxa significantly affected by genotype were marked with stars in Figure 6.

The relative abundance of most bacterial and fungal taxa was significantly correlated with physicochemical properties of substrates (Supplementary Table S9). In waste rock, bacterial and fungal communities were characterized by acid tolerant taxa like the bacterial family *Xanthomonadaceae* and the fungal genus *Acidea*, while the tailings were dominated by microorganisms tolerant to stress like the oligotrophic bacterial genus *Geobacter*. As for the community composition of the control substrate, it was mostly composed of decomposer microorganisms like the bacterial family *Chitinophagaceae* and the fungal genus *Chrysosporium*. A few plant growth parameters were weakly correlated (| 0.3| < *r* < | 0.5|) with the relative abundance of a few bacterial and fungal taxa (Supplementary Table S10).

There was an overall effect of the origin of the cuttings on the relative abundance of bacterial and fungal taxa (Supplementary Table S11). For example, the bacterial genus *Bradyrhizobium* was more abundant in the rhizosphere of genotypes originating from the La Corne Mine site compared to the genotypes originating from the Westwood site, in the control substrate (*p* = 0.030).

## Discussion

### Vegetation Improved Physicochemical and Microbiological Properties of Mine Wastes *in situ*

Mine site restoration aims to mitigate the negative impacts of mining on environmental and human health. Land restoration is a long process since affected ecosystems have lost plant and microbial biodiversity as well as most of their functions and services (Prach and Tolvanen, 2016). Revegetation by pioneer tree species could help initiate the restoration of mine sites. In this study, we first assessed the impact of *P. balsamifera* growth on two contrasting mine wastes *in situ*. These mine wastes were considered unfavorable for plant growth because they contain only small concentrations of essential nutrients, have either a very low or very high pH, and have poor physical structure and deficient water holding capacity (Kumaresan et al., 2017). The pioneer tree *P. balsamifera* has previously been found to naturally grow on mine wastes (Markham et al., 2012), a phenomenon that was also observed in this study. As expected, well established vegetation significantly improved soil health, bringing it closer to a natural forest soil environment. By providing organic matter to mining residues, growth of balsam poplar increased concentrations of C and N as well as several other nutrients. In waste rock, this input of organic matter further led to the observed changes in pH as well as S and Fe concentrations. Indeed, organic matter in soil is known to buffer pH by binding to H^+^ in acidic soils. Similarly, Fe binds to organic matter, making it less available (Johnson et al., 1992). Consequently, it has been shown that S adsorption decreases when organic matter content is higher, pH is higher, and Fe and Al oxides contents are lower, leading to higher S uptake by plants and leaching (Johnson et al., 1992).

In addition to improving physicochemical properties of mine wastes, growth of vegetation also led to a beneficial shift in microbial communities. A greater vegetation presence has been previously shown to increase bacterial richness and diversity in mine wastes, which suggests an improvement in ecosystem productivity and stability (Tilman et al., 2006). After introduction of balsam poplar trees, we observed an overall shift from a lithotrophic to a heterotrophic bacterial community, which is consistent with other studies that found a similar succession of microbial communities during plant growth on mine wastes (Chen et al., 2013; Li et al., 2015, 2016). This shift suggests that initial soil conditions of mine wastes, which favored the growth of lithotrophic microorganisms, changed during the establishment of balsam poplars on site, confirming the positive influence of these trees on microbial community structure and function as well as ecosystem health. For example, *Leptospirillum* and *Acidiphilium*, two bacterial genera associated with the oxidoreduction of iron and acid mine drainage (Harrison, 1981; Hippe, 2000), as well as *Sulfobacillaceae*, a bacterial family associated with the oxidation of sulfur and acid mine drainage (Hottenstein et al., 2019), were found at higher relative abundances in unvegetated than in vegetated zones of the waste rock pile. A previous study also reported that vegetation growth on acid mine tailings increased the pH and lowered the abundance of these key iron and sulfur oxidizing bacteria (Li et al., 2016).

In both our study’s mine sites, *Proteobacteria* were more abundant, and the *Proteobacteria*-to-*Acidobacteria* ratio was higher in vegetated soils than in mine wastes. This corroborates with previous studies showing that this ratio is an indicator of soil trophic level as *Proteobacteria* have been linked to nutrient-rich soils and *Acidobacteria* to nutrient-poor soils (Smit et al., 2001; Fierer et al., 2007). Similarly to our results, Herrera et al. (2007) found that introduction of plants on mine waste increased the abundance of *Proteobacteria* and reduced the abundance of *Acidobacteria*. Furthermore, many rhizobacteria of the orders *Rhizobiales*, *Sphingomonadales* and *Burkholderiales* and the phyla *Planctomycetes*, *Bacteroidetes* and *Actinobacteria* were found at higher relative abundances in vegetated soils than in unvegetated mine wastes. These taxa have previously been associated with the rhizosphere microbiome (da Rocha et al., 2013; McBride et al., 2014; Madhaiyan et al., 2015; Qiao et al., 2017); plant growth promotion (e.g., through nitrogen fixation (Caballero-Mellado et al., 2004; Dai et al., 2014; Sun et al., 2015; Jeanbille et al., 2016); the production of IAA (Mehnaz et al., 2010); disease suppression (Xue et al., 2015), and nutrient cycling (Webb et al., 2014; Santoyo et al., 2016; Wu et al., 2017). Additionally, we observed that vegetation increased the relative abundance of ectomycorrhizal taxa, such as *Meliniomyces*, which have previously been isolated from poplars growing in mine wastes (Gaster et al., 2015; Katanić et al., 2015).

In contrast to our observations for bacteria, growth of vegetation on mine wastes decreased overall fungal richness and diversity. This might be due to the competitive exclusion of ectomycorrhizal fungi on other groups of fungi, corroborating previous studies which showed that forested or restored soils have a lower fungal diversity than soils in disturbed ecosystems (Ding et al., 2011). Surprisingly, the presence of vegetation on mine wastes reduced the relative abundance of fungal taxa assigned as plant pathogens. This could indicate that the assignation of fungal ecological functions based on taxonomy at the genus level may not always be applicable. Indeed, Tedersoo et al. (2014) described their procedure as follows: “If different functional categories were present within a specific genus, we chose the dominant group (> 75% of species assigned to a specific category).” As an example, although the genus *Alternaria* has been assigned as a plant pathogen, some species are known as soil saprotrophs (Thomma, 2003). Additionally, *Alternaria* and some other taxa assigned as plant pathogens, like *Ganoderma* and the *Teratosphaeriaceae* family (Flood et al., 2000; Thomma, 2003; Quaedvlieg et al., 2014), have also been previously isolated in mine wastes and various acidic environments (Wong, 1981; Hujslová et al., 2013; Callender et al., 2016; Mosier et al., 2016).

It was also surprising that the presence of vegetation on mine wastes reduced the relative abundance of fungal saprotrophs as it would be expected that an increase in organic matter would also increase the presence of these microorganisms. These results illustrate a well-known limitation of the use of relative abundances in metabarcoding studies (Zhang et al., 2017; Lin et al., 2019). Although the relative abundance of saprotrophs was lower in the vegetated soil samples, the absolute abundance of these microorganisms might still be higher than in unvegetated mine wastes. This issue could be avoided in future studies by using quantitative PCR to estimate the total populations in these environments (Rastogi et al., 2010) or by spiking samples with exogenous bacteria, fungi, or synthetic DNA prior to processing (Tourlousse et al., 2017).

### Tree Genotype Can Modify the Physicochemical Properties and Microbial Communities of Their Growth Substrates

A greenhouse experiment was conducted in the second part of this study to determine if growth of distinct balsam poplar genotypes can differently impact the physicochemical properties and microbial communities of two contrasting mine substrates. Our results clearly identified substrate type as the main driver of bacterial and fungal community structure and diversity in the rhizosphere of balsam poplar trees. This is most likely due to the very distinct physicochemical properties of the substrates in contrast to the weaker effect of tree genotype. This supports Bonito et al. (2019), who also reported that soil origin and properties influenced microbial communities to a greater degree than host genotype. Additionally, this is a consistent finding among studies on the *Populus* root microbiome (Gottel et al., 2011; Bonito et al., 2014; Veach et al., 2019) and other plant species (Marschner et al., 2004; Lebeis et al., 2015; Wagner et al., 2016; Colin et al., 2017; Gallart et al., 2018), indicating that larger-scale edaphic conditions primarily regulate overall rhizosphere microbiomes. Physicochemical properties, including granulometry and pH, and other unmeasured factors, like water holding capacity and variations in soil temperature, contribute to larger-scale edaphic conditions and likely play a role in the *Populus* root microbiome assembly (Chaparro et al., 2012; Philippot et al., 2013).

Despite important differences between the two mine substrates used in this study, significant and consistent effects of some balsam poplar genotypes on physicochemical properties of their growth substrates were observed. For instance, genotype C29 was associated with higher nutrient content in both substrates, suggesting that this genotype could be well adapted to harsher conditions and has the potential to improve the physicochemical properties of a broad range of substrate types. Interestingly, this genotype was also characterized by its generally lower growth compared to the other genotypes, indicating that common growth parameters may not be suitable as criteria to select the most performant genotypes for revegetation purposes. Conversely, genotypes W08 and N16 led to less favorable physicochemical properties (i.e. lower C and nutrient content) in all substrates, suggesting that these genotypes may be ill-adapted for revegetation purposes.

The effects of genotype on physicochemical properties were not always consistent across substrates, indicating that genotype-by-environment interactions occurred. For example, growth of genotype C21 led to the most significant improvement of the physicochemical properties in tailings but this was not reproduced in waste rock. Interestingly, genotype C21 was collected at the La Corne Mine site, which may suggest a fine-scale local adaptation of this genotype that could be further investigated (Boshier et al., 2015).

The effects of genotype on the rhizosphere microbiome were also generally variable depending on the substrate, as shown by the significant interaction between the substrate type and genotype. Additionally, the effects of genotype on the rhizosphere microbiome were linked to the effects of genotype on physicochemical properties. Indeed, in waste rock, genotype C29 was associated with higher nutrient content and pH as well as bacterial richness when compared to C21, which was associated with the lowest nutrient content, pH, and bacterial richness. These results suggest that genotype selection could further improve soil health by not only modifying the physicochemical properties of a specific substrate, but also by influencing bacterial diversity (Garbeva et al., 2004). Additionally, some balsam poplar genotypes could contribute to the recruitment of specific taxa associated with more favorable soil conditions. For example, *Rhodoplanes* was previously found in the rhizosphere of rice paddy soils irrigated by acid mine drainage contaminated water, and it has been suggested that they may have a beneficial ecological function by enhancing soil fertility (Sun et al., 2015). In our study, genotype C23 was associated with a higher nutrient content and a greater abundance of *Rhodoplanes* when compared to genotype W08 in waste rock, supporting the idea that this bacterial genus may play a role in soil health.

In line with the results from the field experiment, diversity and structure of fungal communities were much more conserved between treatments compared to bacterial communities. All of the most abundant bacterial taxa were affected by substrate type, and several of them were also affected by the tree genotype; however, the impact of these factors on the relative abundance of fungal taxa was much lower. Fungal guild designations also revealed that there was barely any effect of genotype on the relative abundance of fungal functions. This could be explained by the fact that plants recruit taxonomic groups in order to balance functions (Maherali and Klironomos, 2007). In other words, different genotypes may select different taxa, but these taxa ultimately provide similar functions.

Interestingly, there was an overall effect of cutting origin on growth measurements, physicochemical properties of the substrates, and abundance of some bacterial and fungal taxa in each substrate type. This effect could suggest that there may have been a fine-scale adaptation of the genotypes in these novel environments due to high environmental selective pressures. Further research is needed to better assess the effect of this fine-scale adaptation on the composition of the microbiome of poplar trees in such novel environments.

Previous studies have shown that changes in the presence-absence or abundance of just a few microbial taxa can affect plant performance because of their broad functions (Zolla et al., 2013; Henning et al., 2016). In this study, we found that plant genotype had a significant effect on the abundance of some bacterial and fungal taxa, but there was no obvious correlation between the rhizosphere microbiome and tree growth. This may be because of the relatively lenient conditions of our treatments. Indeed, the mine substrates were amended with a peat mix to help plant growth and reduce stress caused by acidity, non-proper hydric conditions, and low nutrient content. Nonetheless, tree growth measurements during the second growth season were influenced by substrate type, showing that longer exposure to the mine substrates could amplify the effect of genotype on the rhizosphere microbiome, which will in turn affect plant growth. Further investigation involving harsher treatments, either directly on the field or with non-amended mine substrates in a greenhouse experiment, would be necessary to better assess the effect of genotype-by-environment interactions on balsam poplar performance and its associated microbiome.

## Conclusion

The aim of this study was to assess the suitability of *P. balsamifera* for the revegetation of abandoned mine sites and to determine the impact of genotype-by-environment interactions on the improvement of soil health and the rhizosphere microbiome. Our results have provided evidence that (1) balsam poplars are suitable to initiate a community of microorganisms closer to a functional vegetated ecosystem on various types of mine wastes in addition to increasing soil nutrient content and improving pH; (2) substrate type has a stronger effect on rhizosphere microbial community composition than genotype; (3) nevertheless, plant genotype can act as a selective pressure in modifying physicochemical properties of the substrate and structuring rhizosphere microbial communities, particularly bacterial taxa; and (4) genotype-by-environment interactions have an impact on the physicochemical properties of substrates and the composition of the rhizosphere microbiome.

From a practical point of view, the selection of tree genotypes together with associated microbiomes benefitting their growth in mine wastes is a strategy that could facilitate the ecological restoration of mine sites. This study confirms the importance of large-scale conditions and environmental heterogeneity on driving soil microbiome assembly, but additionally validates the contribution of plant host genotype in acting as a selective pressure in the surrounding rhizosphere soil. Future research efforts should take into consideration the interdependence between host genotype and associated microbiomes in forest ecosystems, in order to better understand plant-soil feedbacks and incorporate microbiome community ecology into mining restoration strategies.

## Data Availability Statement

The datasets generated in this study can be found in online repositories. The names of the repository/repositories and accession number(s) can be found below: https://www.ncbi.nlm.nih.gov/, PRJNA615167.

## Author Contributions

KR, DL, MG, and AS contributed conception and design of the study. KR, DL, and M-JM contributed to acquisition of data. KR and CM performed the statistical analyses. KR, ÉT, CM, and AS contributed to data interpretation. KR wrote the first draft of the manuscript. CM and NI wrote sections of the manuscript. All authors contributed to manuscript revision, read and approved the submitted version.

## Conflict of Interest

The authors declare that the research was conducted in the absence of any commercial or financial relationships that could be construed as a potential conflict of interest.

## Footnotes

1 https://support.illumina.com/content/dam/illumina-support/documents/documentation/chemistry_documentation/16s/16s-metagenomic-library-prep-guide-15044223-b.pdf
