# Supplementary material for: Plant Genotype Influences Physicochemical Properties of Substrate as Well as Bacterial and Fungal Assemblages in the Rhizosphere of Balsam Poplar
Source: Front Microbiol. 2020 Nov 23;11:575625. doi: 10.3389/fmicb.2020.575625 (PMC7719689; doi:10.3389/fmicb.2020.575625)

Supplementary Figure 1. Pictures of the Westwood site. Sampling of waste rock **(A)**; poplars growing on waste rock **(B)**; screenshot from Google Map of the Westwood site **(C)**; vegetated compared to unvegetated mine waste **(D)**.

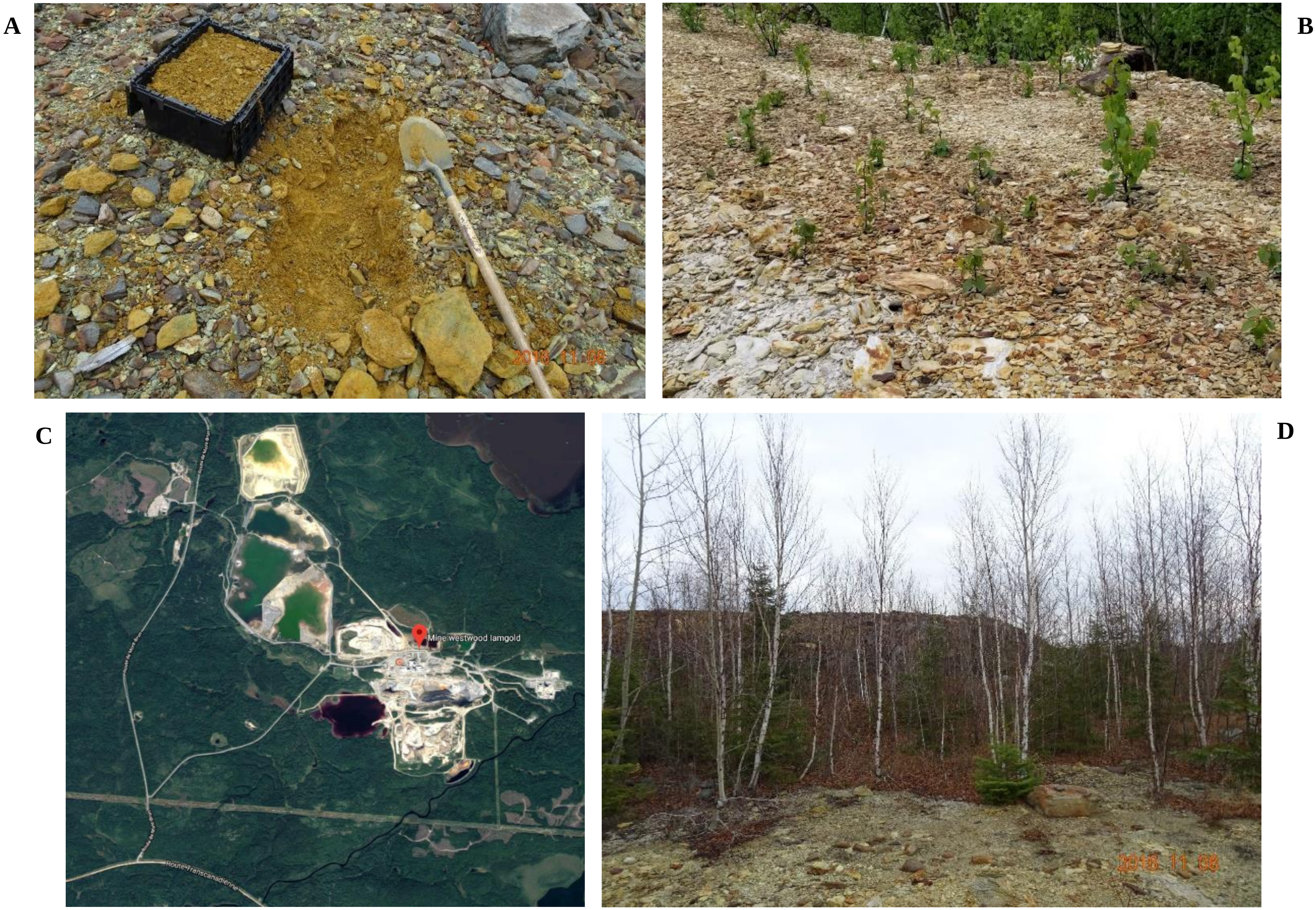

Supplement: Supplementary file 1 [file Image_1.PDF]
