# Supplementary material for: Plant Genotype Influences Physicochemical Properties of Substrate as Well as Bacterial and Fungal Assemblages in the Rhizosphere of Balsam Poplar
Source: Front Microbiol. 2020 Nov 23;11:575625. doi: 10.3389/fmicb.2020.575625 (PMC7719689; doi:10.3389/fmicb.2020.575625)

Supplementary Figure 2. Pictures of the La Corne Mine site. Vegetated compared to unvegetated mine waste **(A)**; sampling of tailings **(B)**; screenshot from Google Map of the La Corne Mine site **(C)**; large view of the vegetation growing in tailings **(D)**.

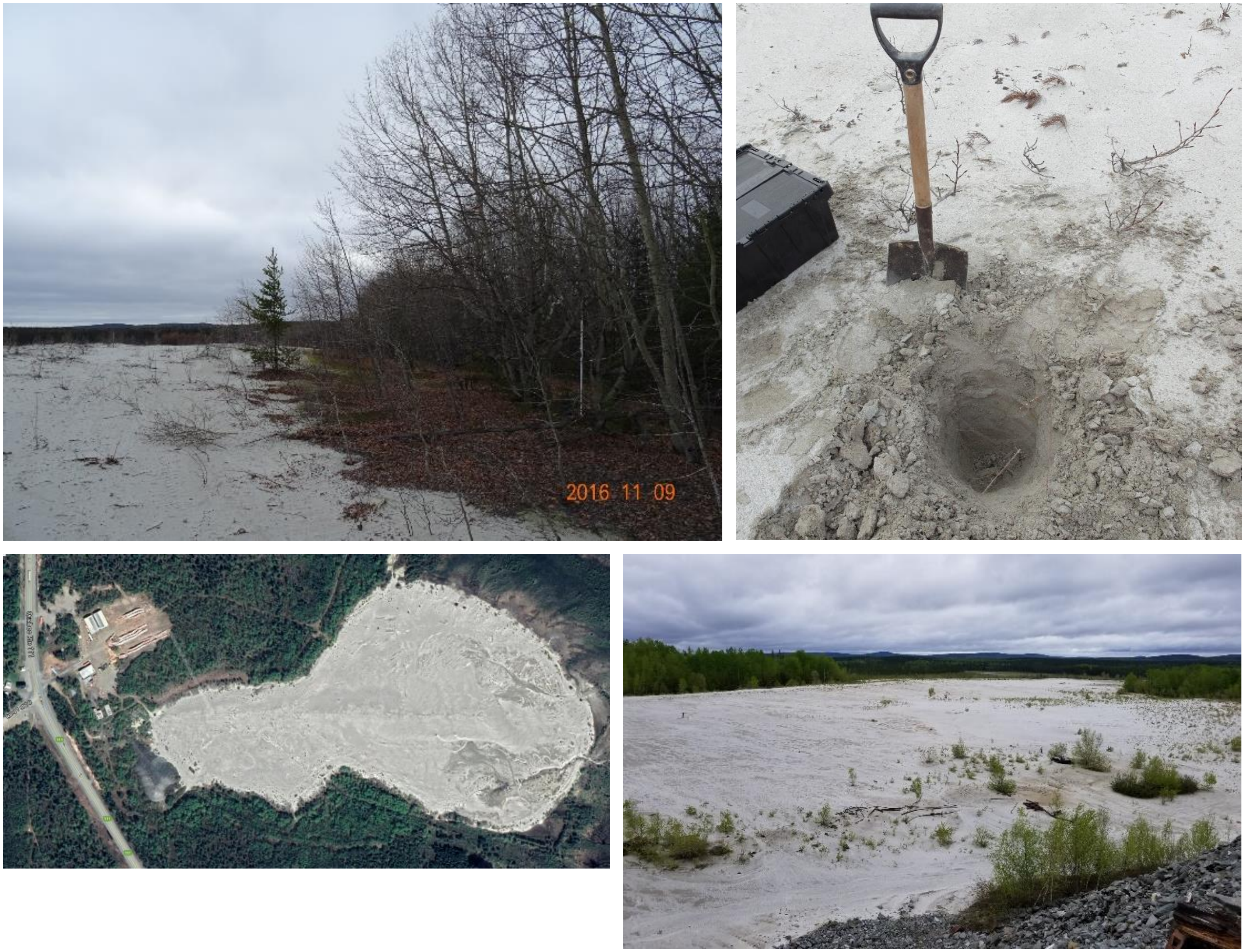

Supplement: Supplementary file 2 [file Image_2.PDF]
