# Supplementary material for: Plant Genotype Influences Physicochemical Properties of Substrate as Well as Bacterial and Fungal Assemblages in the Rhizosphere of Balsam Poplar
Source: Front Microbiol. 2020 Nov 23;11:575625. doi: 10.3389/fmicb.2020.575625 (PMC7719689; doi:10.3389/fmicb.2020.575625)
Supplement: Supplementary file 3 [file Image_3.PDF]

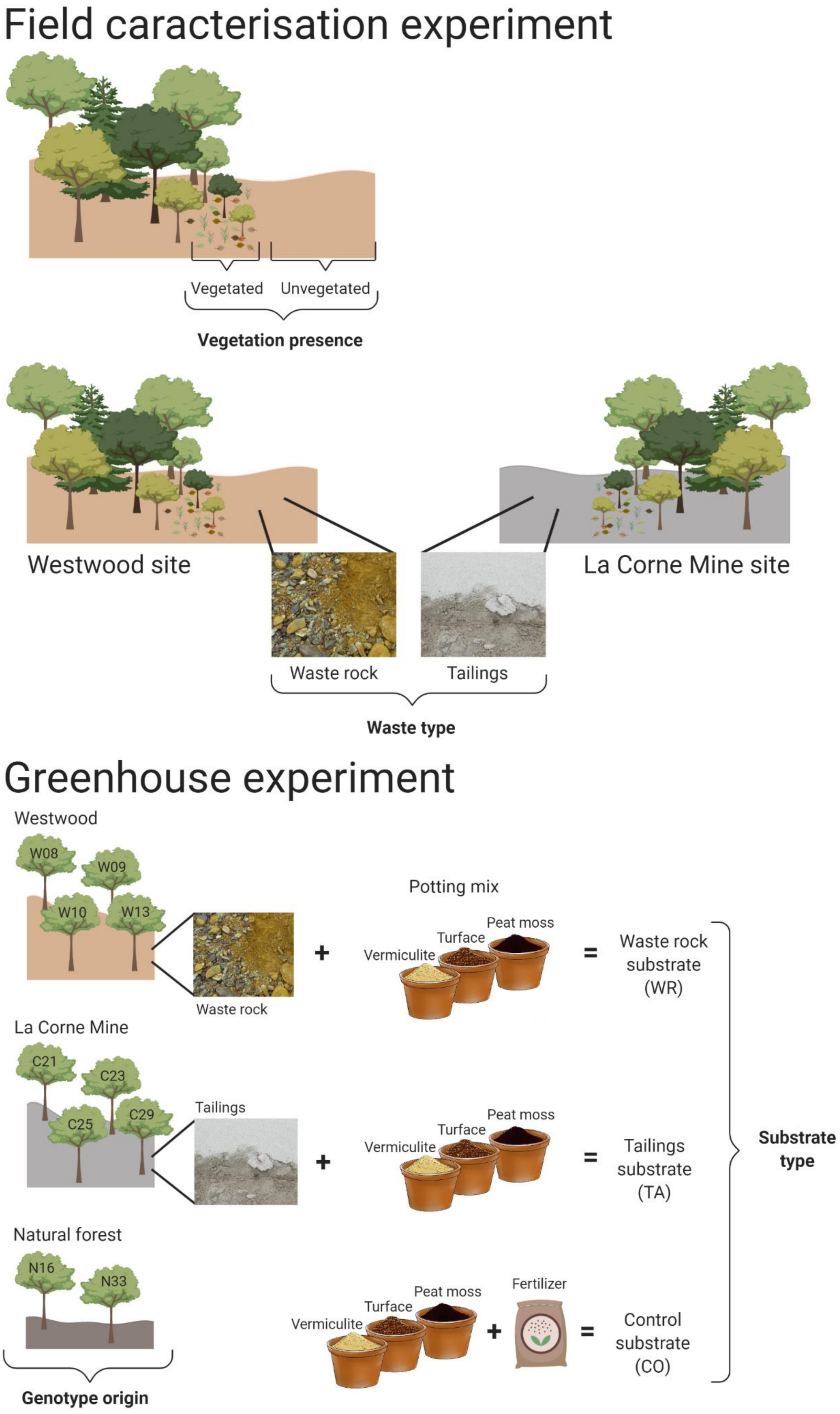
Field caracterisation experiment
Vegetated
Unvegetated
Vegetation presence
Westwood site
La Corne Mine site
Waste rock
Tailings
Waste type
Greenhouse experiment
Westwood
W08
W09
W10
W13
Waste rock
La Corne Mine
C21
C23
C25
C29
Tailings
Natural forest
N16
N33
Genotype origin
Potting mix
Vermiculite
Turface
Peat moss
Fertilizer
Waste rock substrate (WR)
Tailings substrate (TA)
Control substrate (CO)
Substrate type

Supplementary Figure 3. Illustration of the factors in each experiment. Vegetation presence (unvegetated or vegetated) and waste type (waste rock or tailings) for the field experiment. The origin of the 10 genotypes (2 from a natural forest, 4 from Westwood site and 4 from La Corne Mine site) and substrate type (waste rock, tailings or a control substrate) for the greenhouse experiment.
