# Supplementary material for: Plant Genotype Influences Physicochemical Properties of Substrate as Well as Bacterial and Fungal Assemblages in the Rhizosphere of Balsam Poplar
Source: Front Microbiol. 2020 Nov 23;11:575625. doi: 10.3389/fmicb.2020.575625 (PMC7719689; doi:10.3389/fmicb.2020.575625)
Supplement: Supplementary file 4 [file Image_4.PDF]

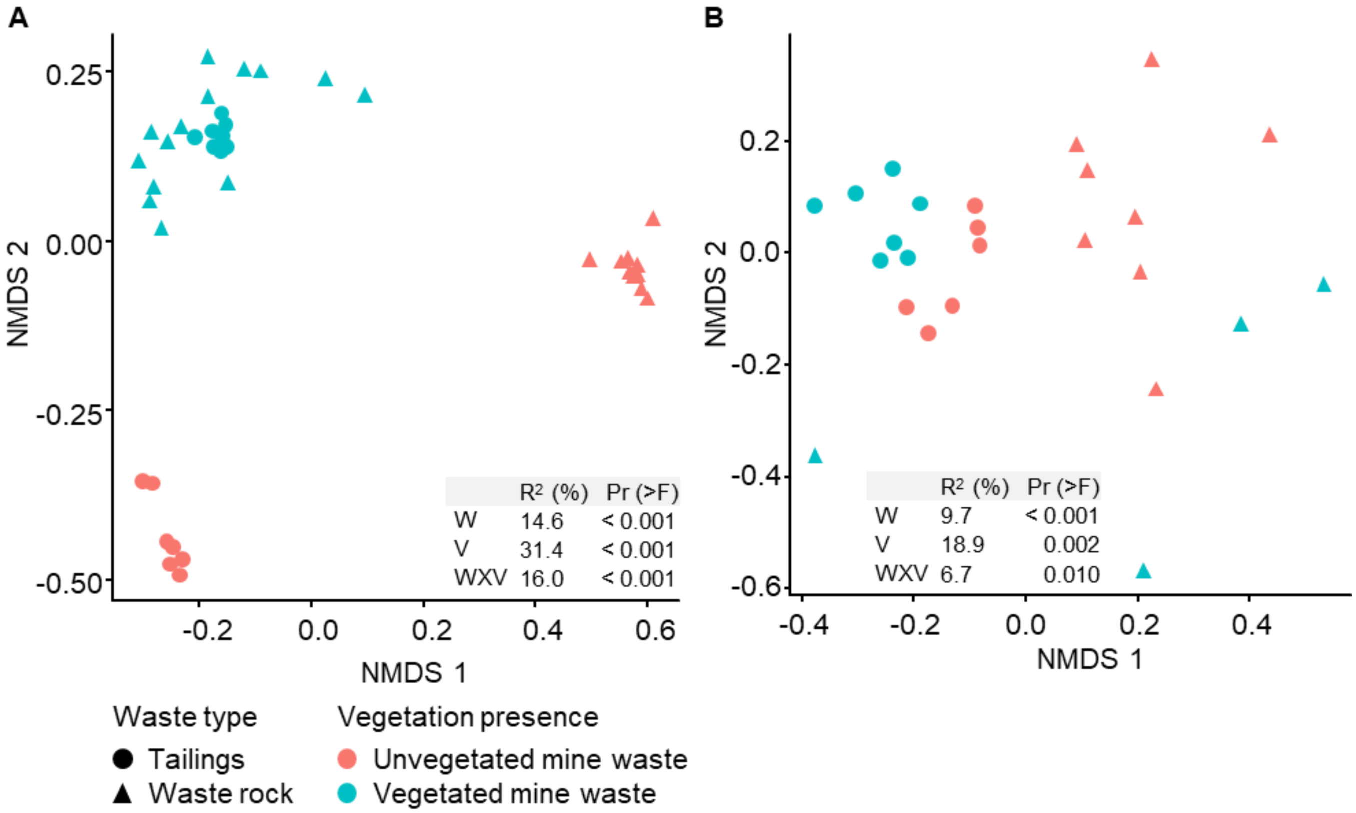

Supplementary Figure 4. Non-metric multidimensional scaling (NMDS) ordination of variation in bacterial and fungal community structure of mining substrates in the field experiment. Bacterial community **(A)** and fungal community **(B)**. A permutational multivariate analysis of variance model was implemented to discern the amount of variation attributed to each factor and their interaction ($n \geq 8$). In PERMANOVA tables, W is for waste type; V is for vegetation presence and WXV for the interaction between waste type and vegetation presence. A multilevel pairwise comparison test revealed that all treatments clustered separately for bacterial communities ($p <$ 0.001) and fungal communities ($p < 0.01$).
