# Supplementary material for: Plant Genotype Influences Physicochemical Properties of Substrate as Well as Bacterial and Fungal Assemblages in the Rhizosphere of Balsam Poplar
Source: Front Microbiol. 2020 Nov 23;11:575625. doi: 10.3389/fmicb.2020.575625 (PMC7719689; doi:10.3389/fmicb.2020.575625)
Supplement: Supplementary file 5 [file Image_5.PDF]

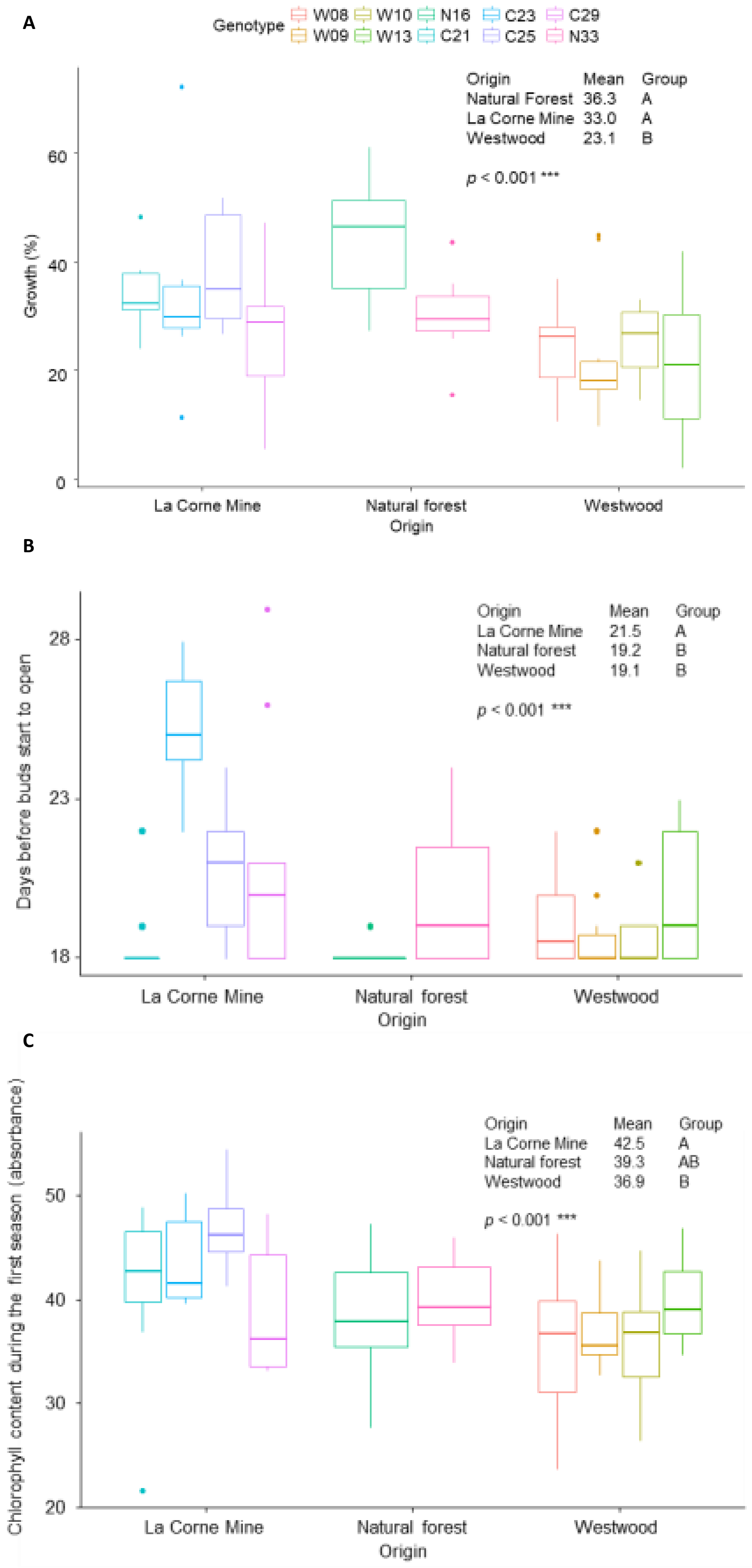
A
Genotype
W08
W09
W10
W13
N16
C21
C23
C25
C29
N33
Origin Mean Group
Natural Forest 36.3 A
La Corne Mine 33.0 A
Westwood 23.1 B
p < 0.001 ***
Growth (%)
0
20
40
60
La Corne Mine
Natural forest
Westwood
Origin
B
Origin Mean Group
La Corne Mine 21.5 A
Natural forest 19.2 B
Westwood 19.1 B
p < 0.001 ***
Days before buds start to open
18
23
28
La Corne Mine
Natural forest
Westwood
Origin
C
Origin Mean Group
La Corne Mine 42.5 A
Natural forest 39.3 AB
Westwood 36.9 B
p < 0.001 ***
Chlorophyll content during the first season (absorbance)
20
30
40
50
La Corne Mine
Natural forest
Westwood
Origin

Supplementary Figure 5. Effect of the origin of cuttings on their growth during the first season. Tukey HSD *post hoc* pairwise comparison tests were used to discern how the origin of cuttings influenced their growth ($n \geq 3$). Measurements from the first season only are shown because the origin of cuttings did not have a significant effect on tree growth measurements for the second season.
