# Supplementary material for: Plant Genotype Influences Physicochemical Properties of Substrate as Well as Bacterial and Fungal Assemblages in the Rhizosphere of Balsam Poplar
Source: Front Microbiol. 2020 Nov 23;11:575625. doi: 10.3389/fmicb.2020.575625 (PMC7719689; doi:10.3389/fmicb.2020.575625)
Supplement: Supplementary file 6 [file Image_6.PDF]

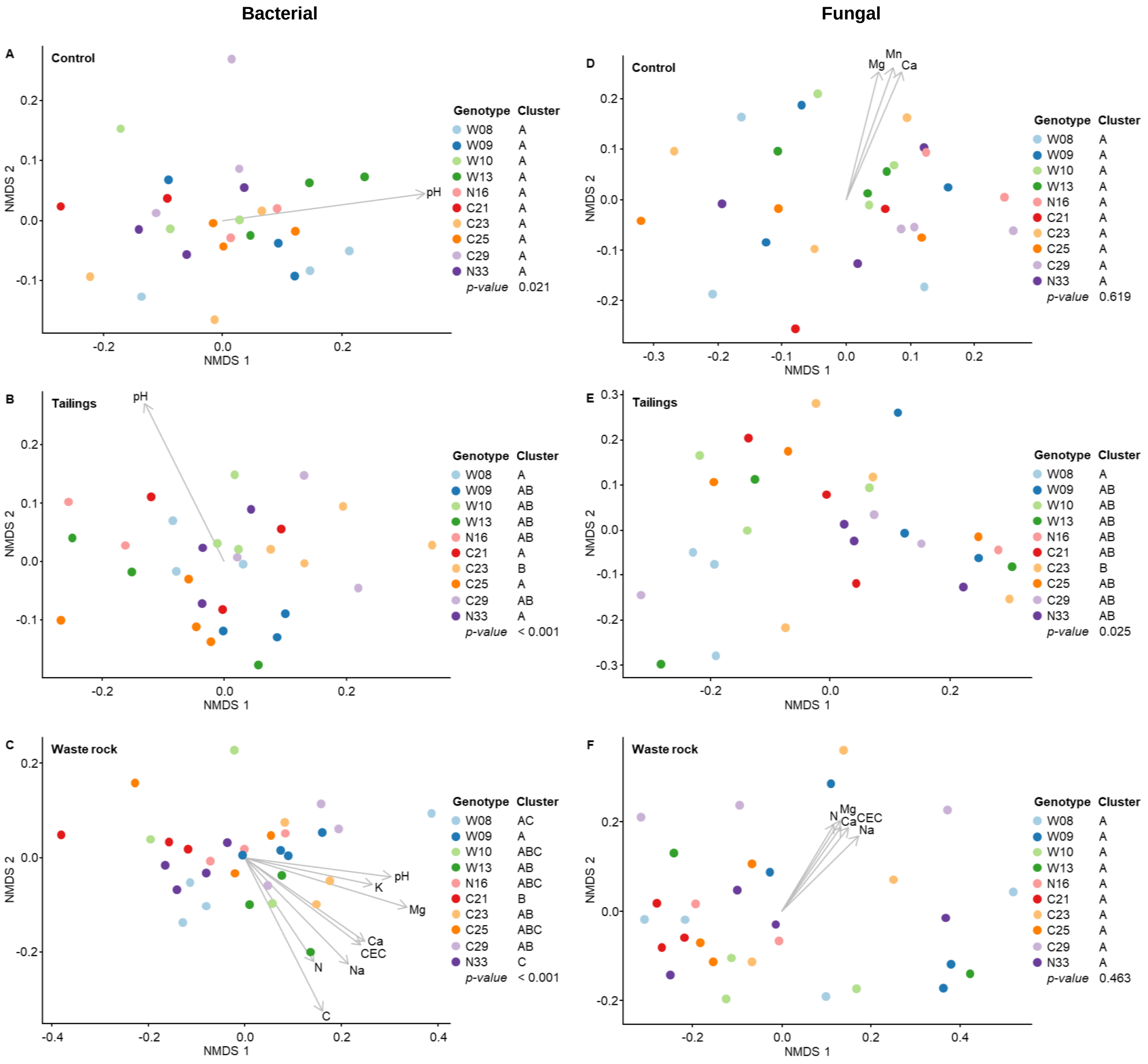

Supplementary Figure 6. Non-metric multidimensional scaling (NMDS) ordination of variation in bacterial and fungal community structure in substrates from the greenhouse experiment. Bacterial community structure in control substrate **(A)**, Tailings **(B)** and Waste rock **(C)** and fungal community structure in control substrate **(D)**, Tailings **(E)** and Waste rock **(F)**. Points represent samples and arrows represent the significant correlations between NMDS axes and the physicochemical properties of the substrates. Clusters between genotypes were determined by a multilevel pairwise comparison test (n ≥ 3).
