# Supplementary material for: Plant Genotype Influences Physicochemical Properties of Substrate as Well as Bacterial and Fungal Assemblages in the Rhizosphere of Balsam Poplar
Source: Front Microbiol. 2020 Nov 23;11:575625. doi: 10.3389/fmicb.2020.575625 (PMC7719689; doi:10.3389/fmicb.2020.575625)
Supplement: Supplementary file 7 [file Table_1.PDF]

Supplementary Table 1. Balsam poplar genotyping on a Sequenom intraspecific panel. Trees were sampled at the Westwood site (W), the La Corne Mine site (C) and in a natural forest nearby of the La Corne Mine site (N). Unique genotypes were verified using a 40 SNP-array designed to reveal *P. balsamifera* intraspecific variations.

Sample	004	006	010	011	012	018	020	021	022	024	029	031	032	036	038	040	043	047	049	053	057	061	063	064	068	071	074	075
**W01**	CC	AA	CT	TT	GG	AA	TT	CC	GT	AA	TT	CC	CT	AA	TT	AA	AA	AG	CC	CC	GT	GG	CC	CC	AA	TT	TT	GG
**W03**	TT	AA	CT	TT	GG	AA	TT	CC	TT	AT	TT	CC	CC	GG	TT	AG	AA	AA	CC	CT	GT	GG	TT	CT	AA	CT	TT	GG
**W05**	CT	AA	TT	TT	AG	AA	TT	CC	GT	TT	TT	CC	CT	AA	CT	AG	AG	GG	CC	CC	TT	CG	CC	CC	AA	CC	CC	GG
**W08**	TT	AA	CT	AT	GG	AA	TT	CC	GT	AA	CT	CC	CT	0	CT	AG	AA	GG	CC	CC	TT	GG	CT	CC	AA	CT	CT	GG
**W09**	CT	AA	CC	TT	AG	AA	CT	CC	GT	AT	TT	CC	CT	AG	TT	AG	AA	GG	CC	CT	GT	GG	CT	CC	AA	CT	TT	GG
**W10**	TT	AA	CT	TT	AG	AA	CT	CC	GT	AA	CT	CC	CT	AG	TT	AA	AA	GG	CC	CC	GG	GG	CT	CT	AA	TT	TT	GT
**W12**	TT	AA	CC	AA	AA	AA	CT	TT	GT	AT	CT	CC	CT	GG	TT	AA	AA	AG	AC	CC	GG	CG	CT	CC	AA	CT	CT	GG
**W13**	CT	AA	CT	TT	AG	AA	TT	CT	GG	AA	CC	CC	TT	AA	CT	AA	AG	GG	CC	CC	GT	GG	TT	CC	AA	TT	CT	GG
**N16**	CT	AA	CT	AT	GG	AA	CT	CT	TT	AA	CT	CC	CC	AA	CT	AG	AA	AG	CC	CC	GG	CG	CT	CC	AA	TT	CC	GG
**C17**	TT	AA	CT	AT	GG	AA	TT	TT	GT	AA	CT	AC	CC	AG	CT	AG	AA	GG	AC	CT	TT	GG	TT	CC	AA	CT	CT	GG
**C19**	CC	AC	CT	AT	GG	AA	TT	CT	GT	AA	CT	CC	CT	AA	TT	AA	AA	GG	CC	CC	GT	GG	TT	0	AA	CT	CC	GG
**C21**	TT	AA	CT	TT	AG	AA	TT	TT	GT	AT	CT	CC	CC	AG	CT	AG	AA	GG	CC	CC	TT	CG	CT	CC	AT	TT	CT	GG
**C23**	TT	AA	CT	TT	GG	AA	CT	CC	GT	AT	CC	CC	CC	AA	TT	AG	AA	AG	AC	CC	GG	CG	CT	CC	AA	TT	TT	GG
**C25**	TT	AA	CC	AT	GG	AA	TT	TT	GT	AT	CT	CC	CT	GG	CT	AG	AA	GG	CC	CT	GT	CG	CT	CT	AA	TT	TT	GG
**C27**	TT	AC	CT	TT	AG	AA	TT	CC	GT	AA	CT	CC	CC	AG	TT	GG	AA	GG	CC	CC	GG	GG	CT	CT	AA	TT	TT	GG
**C29**	TT	AA	CT	TT	GG	AA	TT	CT	GT	AA	CT	CC	CT	AG	CT	AA	AA	AG	CC	CT	GT	GG	TT	TT	AA	TT	CT	GT
**C30**	TT	AA	CT	TT	AG	AA	CT	CT	GG	AT	TT	CC	CC	AA	TT	AA	AA	GG	AC	CC	GT	GG	CT	CC	AA	TT	TT	GG
**N33**	TT	CC	CT	TT	GG	AA	CT	CC	GG	AA	CT	CC	CC	AA	TT	AA	AA	GG	CC	TT	GT	GG	CT	CT	AA	CT	CT	GG
