# Supplementary material for: Plant Genotype Influences Physicochemical Properties of Substrate as Well as Bacterial and Fungal Assemblages in the Rhizosphere of Balsam Poplar
Source: Front Microbiol. 2020 Nov 23;11:575625. doi: 10.3389/fmicb.2020.575625 (PMC7719689; doi:10.3389/fmicb.2020.575625)
Supplement: Supplementary file 8 [file Table_2.pdf]

Supplementary Table 2. Pairwise comparison between all treatments on the field for bacterial and fungal taxa abundances and fungal functions. Two-way ANOVAs were used to discern how waste type, vegetation presence and their interaction influenced taxa relative abundance and fungal functions relative abundance. When a factor was revealed as a statistically significant predictor, a Tukey HSD *post hoc* pairwise comparison test was performed between all treatments.

**Bacteria**		*Acetobacteraceae_g*	*Acidimicrobiales_f_g*	*Acidiphilium*	*Acidobacteriaceae_g*	*Actinomycetales_f_g*	*[Pedosphaerales] auto67-4W_g*
	Waste type	< 0.001	0.072	< 0.001	< 0.001	0.061	0.325
	Vegetation presence	< 0.001	< 0.001	< 0.001	< 0.001	< 0.001	< 0.001
	Interaction	< 0.001	0.047	< 0.001	< 0.001	0.038	0.524
**Pairwise comparison**							
Tailings	Unvegetated	1.9% B	16.0% A	0.0% B	0.0% C	0.0% C	0.1% B
	Vegetated	1.4% B	0.1% B	0.0% B	2.4% B	1.2% A	1.0% A
Waste rock	Unvegetated	5.3% A	6.7% A	3.2% A	13.6% A	0.1% BC	0.0% B
	Vegetated	2.0% B	0.2% B	0.0% B	3.7% B	0.7% AB	0.8% A
	p-value	< 0.001	< 0.001	< 0.001	< 0.001	< 0.001	< 0.001

**Fungi**		*Acidea*	*Agaricales_f_g*	*Alternaria*	*Amphinema*	*Apiotrichum*	*Ascomycota _o_c_f_g*
	Waste type	0.188	0.352	0.697	0.068	0.664	0.038
	Vegetation presence	0.294	0.418	0.029	< 0.001	0.473	0.665
	Interaction	0.944	0.418	0.028	0.003	0.257	0.364
**Pairwise comparison**							
Tailings	Unvegetated	0.4% A	0.0% A	5.5% A	1.4% A	0.7% A	1.2% A
	Vegetated	0.0% A	2.5% A	0.1% B	0.0% C	0.1% A	1.6% A
Waste rock	Unvegetated	2.5% A	0.0% A	0.5% AB	0.2% B	0.1% A	0.3% A
	Vegetated	0.2% A	0.0% A	0.3% B	0.0% C	1.7% A	0.0% A
	p-value	0.392	0.497	0.004	< 0.001	0.531	0.078

**Fungal functions**		Ectomycorrhizal	Saprotroph	Ericoid	Plant pathogen	Lichenized	White rot
	Waste type	0.047	0.169	0.133	0.337	0.056	0.178
	Vegetation presence	0.008	< 0.001	0.002	< 0.001	0.958	0.067
	Interaction	0.358	0.773	0.340	0.078	0.398	0.738
**Pairwise comparisons**							
Tailings	Unvegetated	22.2% B	37.5% A	2.2% B	7.9% A	3.8% A	1.7% A
	Vegetated	68.2% A	8.9% B	7.3% A	0.4% C	2.2% A	0.1% A
Waste rock	Unvegetated	11.8% B	43.2% A	10.5% AB	4.2% AB	0.7% A	2.3% A
	Vegetated	36.4% AB	19.4% AB	35.6% AB	0.8% BC	0.9% A	0.0% A
	p-value	< 0.001	< 0.001	< 0.001	< 0.001	0.142	0.092

Supplementary Table 2. Pairwise comparison between all treatments on the field for bacterial and fungal taxa abundances and fungal functions. Two-way ANOVAs were used to discern how waste type, vegetation presence and their interaction influenced taxa relative abundance and fungal functions relative abundance. When a factor was revealed as a statistically significant predictor, a Tukey HSD *post hoc* pairwise comparison test was performed between all treatments.

**Bacteria**		*Bradyrhizobium*	*Burkholderia*	*Chloroflexi C0119_o_f_g*	*Candidatus Koribacter*	*Candidatus Nitrososphaera*	*Chitinophagaceae_g*
	Waste type	< 0.001	0.333	< 0.001	< 0.001	0.018	< 0.001
	Vegetation presence	< 0.001	< 0.001	0.591	0.012	0.024	< 0.001
	Interaction	0.008	0.182	< 0.001	0.005	0.018	< 0.001
**Pairwise comparison**							
Tailings	Unvegetated	1.2% C	0.0% B	1.7% A	4.1% A	1.6% A	3.9% B
	Vegetated	7.8% A	3.8% A	0.0% B	0.4% B	0.0% B	6.7% AB
Waste rock	Unvegetated	0.0% D	0.0% B	0.0% B	0.0% B	0.0% B	0.0% C
	Vegetated	4.7% B	6.7% A	0.1% B	0.6% B	0.0% B	7.1% A
	p-value	< 0.001	< 0.001	< 0.001	< 0.001	< 0.001	< 0.001

**Fungi**		*Cenococcum*	*Chaetosphaeriaceae_g*	*Chaetothyriales_g*	*Cistella*	*Cladophialophora*	*Cladosporium*
	Waste type	< 0.001	0.318	0.887	0.977	0.112	0.937
	Vegetation presence	0.144	0.001	0.002	0.009	0.485	0.003
	Interaction	0.102	0.065	0.671	0.027	0.186	0.092
**Pairwise comparison**							
Tailings	Unvegetated	11.8% AB	1.2% A	1.2% A	4.3% A	0.1% A	11.7% A
	Vegetated	15.8% A	0.0% B	0.0% B	0.2% B	0.0% A	0.3% B
Waste rock	Unvegetated	3.5% BC	0.3% AB	0.9% AB	0.9% AB	13.1% A	3.8% AB
	Vegetated	0.0% C	0.0% B	0.0% AB	0.3% B	0.1% A	0.8% B
	p-value	< 0.001	< 0.001	< 0.001	< 0.001	0.139	< 0.001

**Fungal functions**		Mycoparasite	Arbuscular
	Waste type	0.170	0.270
	Vegetation presence	0.021	0.208
	Interaction	0.220	0.208
**Pairwise comparisons**			
Tailings	Unvegetated	0.2% A	0.2% A
	Vegetated	0.0% A	0.0% B
Waste rock	Unvegetated	1.5% A	0.0% B
	Vegetated	0.0% A	0.0% AB
	p-value	0.025	0.003

Supplementary Table 2. Pairwise comparison between all treatments on the field for bacterial and fungal taxa abundances and fungal functions. Two-way ANOVAs were used to discern how waste type, vegetation presence and their interaction influenced taxa relative abundance and fungal functions relative abundance. When a factor was revealed as a statistically significant predictor, a Tukey HSD *post hoc* pairwise comparison test was performed between all treatments.

**Bacteria**		*Cytophagaceae_g*	*[Chthoniobacteraceae] DA101*	*Deinococcus*	*Acidobacteria iii1-8 DS-18_f_g*	*Thermoplasmata E2_f_g*	*Alphaproteobacteria Ellin329_f_g*
	Waste type	0.146	< 0.001	< 0.001	< 0.001	0.010	0.085
	Vegetation presence	0.015	0.281	< 0.001	0.002	0.014	< 0.001
	Interaction	0.034	< 0.001	< 0.001	< 0.001	0.010	0.002
**Pairwise comparison**							
Tailings	Unvegetated	0.2% AB	6.0% A	1.2% A	3.3% A	1.7% A	0.0% C
	Vegetated	0.2% AB	0.6% B	0.0% B	0.2% B	0.0% B	2.8% A
Waste rock	Unvegetated	0.0% B	0.0% B	0.0% B	0.0% B	0.0% B	0.0% C
	Vegetated	0.8% A	0.1% B	0.0% B	0.1% B	0.0% B	1.7% B
	p-value	0.001	< 0.001	< 0.001	< 0.001	< 0.001	< 0.001

**Fungi**		*Clavulinopsis*	*Cortinarius*	*Cryptococcus*	*Exophiala*	*Ganoderma*	*Geomyces*
	Waste type	< 0.001	0.259	0.302	0.006	0.267	0.119
	Vegetation presence	0.015	0.002	0.209	< 0.001	0.019	0.011
	Interaction	0.016	0.298	0.098	< 0.001	0.047	0.681
**Pairwise comparison**							
Tailings	Unvegetated	0.2% B	0.8% AB	0.9% A	1.4% A	1.1% A	1.8% A
	Vegetated	2.6% A	14.6% A	0.7% A	0.0% B	0.0% B	0.2% A
Waste rock	Unvegetated	0.0% B	0.3% B	0.4% A	0.0% B	0.2% AB	2.7% A
	Vegetated	0.0% B	31.2% A	2.2% A	0.1% B	0.0% B	0.0% A
	p-value	< 0.001	< 0.001	0.097	< 0.001	0.003	0.075

Supplementary Table 2. Pairwise comparison between all treatments on the field for bacterial and fungal taxa abundances and fungal functions. Two-way ANOVAs were used to discern how waste type, vegetation presence and their interaction influenced taxa relative abundance and fungal functions relative abundance. When a factor was revealed as a statistically significant predictor, a Tukey HSD *post hoc* pairwise comparison test was performed between all treatments.

**Bacteria**		*Betaproteobacteria Ellin6067_f_g*	*Acidobacteria DA052 Ellin6513_f_g*	*Flavobacterium*	*Gaiellaceae_g*	*Gammaproteobacteria _o_f_g*	*Gemmata*
	Waste type	< 0.001	< 0.001	0.004	0.100	< 0.001	0.157
	Vegetation presence	0.393	< 0.001	< 0.001	< 0.001	0.065	0.002
	Interaction	0.004	< 0.001	0.003	0.224	< 0.001	0.005
**Pairwise comparison**							
Tailings	Unvegetated	1.2% A	0.0% C	0.0% B	1.2% A	0.3% B	2.0% A
	Vegetated	0.8% AB	7.2% A	0.4% A	0.8% AB	0.0% B	0.6% B
Waste rock	Unvegetated	0.0% C	0.0% C	0.0% B	0.0% C	14.2% A	0.0% C
	Vegetated	0.6% B	2.5% B	1.2% A	0.6% B	0.2% B	0.8% B
	p-value	< 0.001	< 0.001	< 0.001	< 0.001	< 0.001	< 0.001

**Fungi**		*Hebeloma*	*Inocybe*	*Knufia*	*Lecanoromycetes_g*	*Leotiomycetes_c_f_g*	*Leptodontidium*
	Waste type	0.291	0.288	0.214	< 0.001	0.355	0.854
	Vegetation presence	0.227	0.827	0.160	< 0.001	0.592	0.357
	Interaction	0.227	0.441	0.530	< 0.001	0.394	0.037
**Pairwise comparison**							
Tailings	Unvegetated	0.0% A	0.5% A	0.0% A	0.0% B	1.6% A	0.0% A
	Vegetated	0.0% A	8.4% A	0.1% A	1.3% A	1.5% A	0.2% A
Waste rock	Unvegetated	0.0% A	1.2% A	0.2% A	0.0% B	3.0% A	3.0% A
	Vegetated	4.8% A	0.2% A	1.2% A	0.0% B	0.1% A	0.0% A
	p-value	0.207	0.582	0.214	< 0.001	0.565	0.081

Supplementary Table 2. Pairwise comparison between all treatments on the field for bacterial and fungal taxa abundances and fungal functions. Two-way ANOVAs were used to discern how waste type, vegetation presence and their interaction influenced taxa relative abundance and fungal functions relative abundance. When a factor was revealed as a statistically significant predictor, a Tukey HSD *post hoc* pairwise comparison test was performed between all treatments.

**Bacteria**		*Gemmataceae_g*	*Acidobacteria-6 iii1-15_f_g*	*Isosphaeraceae_g*	*AD3 JG37-AG-4 _o_f_g*	*Kaistobacter*	*Koribacteraceae_g*	*Leptospirillum*
	Waste type	< 0.001	0.293	< 0.001	0.604	< 0.001	0.005	0.360
	Vegetation presence	0.285	< 0.001	< 0.001	0.927	< 0.001	< 0.001	< 0.001
	Interaction	< 0.001	0.044	0.477	0.001	< 0.001	< 0.001	< 0.001
**Pairwise comparison**								
Tailings	Unvegetated	4.7% AB	1.1% B	0.2% B	0.0% C	4.8% A	0.0% C	0.0% B
	Vegetated	2.6% B	2.0% A	1.6% A	0.1% B	0.0% C	7.9% A	0.0% B
Waste rock	Unvegetated	7.1% A	0.0% C	1.9% A	8.4% A	0.0% C	0.0% C	7.2% A
	Vegetated	1.6% B	1.3% AB	0.5% AB	0.6% BC	1.2% B	2.7% B	0.0% B
	p-value	< 0.001	< 0.001	< 0.001	< 0.001	< 0.001	< 0.001	< 0.001

**Fungi**		*Lipomyces*	*Meliniomyces*	*Oidiodendron*	*Parmelia*	*Penicillium*	*Pezoloma*	*Phialocephala*
	Waste type	0.134	0.114	0.412	0.022	0.155	0.258	0.011
	Vegetation presence	0.188	0.006	0.575	0.035	0.035	0.101	0.074
	Interaction	0.188	0.237	0.788	0.634	0.706	0.657	0.694
**Pairwise comparison**								
Tailings	Unvegetated	0.0% A	1.0% B	1.2% A	3.8% A	0.0% B	1.8% A	6.2% AB
	Vegetated	0.0% A	5.8% A	1.5% A	2.2% AB	0.7% AB	0.1% A	1.8% A
Waste rock	Unvegetated	1.2% A	7.0% AB	3.5% A	0.7% AB	0.5% AB	6.0% A	3.3% AB
	Vegetated	0.0% A	32.8% AB	2.7% A	0.0% B	1.6% A	1.2% A	0.4% B
	p-value	0.083	< 0.001	0.766	0.003	0.007	0.178	0.003

Supplementary Table 2. Pairwise comparison between all treatments on the field for bacterial and fungal taxa abundances and fungal functions. Two-way ANOVAs were used to discern how waste type, vegetation presence and their interaction influenced taxa relative abundance and fungal functions relative abundance. When a factor was revealed as a statistically significant predictor, a Tukey HSD *post hoc* pairwise comparison test was performed between all treatments.

**Bacteria**		*[Methanomassiliicoccaceae]_g*	*Methylobacterium*	*Methylocystaceae_g*	*Mycobacterium*	*Oxalobacteraceae_g*
	Waste type	0.376	0.039	< 0.001	0.011	0.003
	Vegetation presence	0.007	< 0.001	< 0.001	< 0.001	< 0.001
	Interaction	0.007	0.105	< 0.001	0.276	0.489
**Pairwise comparison**						
Tailings	Unvegetated	0.2% B	1.4% A	0.3% B	0.0% B	1.4% A
	Vegetated	0.0% B	0.0% B	1.3% A	0.6% A	0.4% AB
Waste rock	Unvegetated	2.8% A	0.0% B	0.0% B	0.1% B	0.0% C
	Vegetated	0.0% B	0.0% B	0.7% AB	1.4% A	0.3% B
	p-value	< 0.001	< 0.001	< 0.001	< 0.001	< 0.001

**Fungi**		*Piloderma*	*Pleosporales_ fam_Incertae_sedis_g*	*Pyrenopeziza*	*Rozellomycota_ unidentified*	*Sagenomella*
	Waste type	< 0.001	0.476	0.133	0.084	0.375
	Vegetation presence	0.214	0.077	0.061	< 0.001	0.888
	Interaction	0.002	0.204	0.464	0.379	0.435
**Pairwise comparison**						
Tailings	Unvegetated	4.7% B	4.6% A	0.4% A	0.0% A	0.1% A
	Vegetated	19.7% A	0.1% A	0.0% A	3.0% A	0.1% A
Waste rock	Unvegetated	2.0% BC	0.7% A	1.8% A	0.0% A	1.2% A
	Vegetated	0.0% C	0.4% A	0.2% A	0.6% A	0.1% A
	p-value	< 0.001	0.099	0.053	0.057	0.672

Supplementary Table 2. Pairwise comparison between all treatments on the field for bacterial and fungal taxa abundances and fungal functions. Two-way ANOVAs were used to discern how waste type, vegetation presence and their interaction influenced taxa relative abundance and fungal functions relative abundance. When a factor was revealed as a statistically significant predictor, a Tukey HSD *post hoc* pairwise comparison test was performed between all treatments.

**Bacteria**		*Planctomyces*	*Proteobacteria _c_o_f_g*	*Rhodoplanes*	*Rhodospirillaceae_g*	*Rubrivivax*	*Sinobacteraceae_g*
	Waste type	< 0.001	0.397	0.148	0.021	0.210	< 0.001
	Vegetation presence	0.021	0.008	< 0.001	< 0.001	< 0.001	0.032
	Interaction	< 0.001	0.006	0.001	0.087	0.558	< 0.001
**Pairwise comparison**							
Tailings	Unvegetated	0.9% A	0.1% B	0.4% B	0.2% B	1.2% A	0.3% C
	Vegetated	0.9% A	0.0% B	1.2% A	2.8% A	0.2% B	10.6% A
Waste rock	Unvegetated	0.0% B	4.0% A	0.0% C	0.0% B	0.0% C	5.3% B
	Vegetated	1.3% A	0.0% B	1.0% AB	2.3% A	0.1% B	4.1% B
	p-value	< 0.001	< 0.001	< 0.001	< 0.001	< 0.001	< 0.001

**Fungi**		*Setophoma*	*Sistotrema*	*Sordariales_g*	*Talaromyces*	*Teratosphaeriaceae_g*	*Tetracladium*
	Waste type	0.217	0.078	0.145	0.133	0.025	0.455
	Vegetation presence	0.021	0.047	0.477	0.188	0.045	0.004
	Interaction	0.717	0.047	0.179	0.188	0.045	0.231
**Pairwise comparison**							
Tailings	Unvegetated	1.7% A	0.0% A	4.9% A	0.0% A	0.0% B	2.0% A
	Vegetated	0.1% A	0.0% A	0.4% A	0.0% A	0.0% B	0.1% B
Waste rock	Unvegetated	2.3% A	0.0% A	0.0% A	1.2% A	4.4% A	1.0% AB
	Vegetated	0.0% A	11.4% A	0.0% A	0.0% A	0.0% B	0.1% B
	p-value	0.025	0.262	0.162	0.083	< 0.001	0.001

Supplementary Table 2. Pairwise comparison between all treatments on the field for bacterial and fungal taxa abundances and fungal functions. Two-way ANOVAs were used to discern how waste type, vegetation presence and their interaction influenced taxa relative abundance and fungal functions relative abundance. When a factor was revealed as a statistically significant predictor, a Tukey HSD *post hoc* pairwise comparison test was performed between all treatments.

**Bacteria**		*Solibacterales_f_g*	*Solirubrobacterales_f_g*	*Sphingobacteriaceae_g*	*Sphingobacteriales_f_g*	*Sphingomonas*
	Waste type	0.321	0.004	0.349	0.188	0.882
	Vegetation presence	< 0.001	< 0.001	< 0.001	< 0.001	< 0.001
	Interaction	< 0.001	0.427	< 0.001	0.010	0.008
**Pairwise comparison**						
Tailings	Unvegetated	0.8% B	3.0% A	0.1% C	0.4% BC	0.3% B
	Vegetated	2.4% A	0.2% B	2.7% B	0.8% AB	0.5% AB
Waste rock	Unvegetated	0.0% C	0.3% B	0.0% C	0.0% C	0.0% C
	Vegetated	2.0% A	0.3% B	5.4% A	1.1% A	4.1% A
	p-value	< 0.001	< 0.001	< 0.001	< 0.001	< 0.001

**Fungi**		*Tomentella*	*Tremella*	*Tricholoma*	*Umbelopsis*	*Venturia*
	Waste type	0.448	0.144	0.032	0.626	0.048
	Vegetation presence	0.285	0.043	0.054	0.209	0.051
	Interaction	0.693	0.248	0.159	0.391	0.086
**Pairwise comparison**						
Tailings	Unvegetated	0.0% A	0.2% A	2.3% A	1.3% A	0.2% AB
	Vegetated	0.0% A	0.0% A	8.9% A	1.4% A	0.2% B
Waste rock	Unvegetated	2.6% A	1.5% A	1.0% A	2.7% A	2.2% A
	Vegetated	0.0% A	0.0% A	0.0% A	0.5% A	0.5% AB
	p-value	0.559	0.025	0.004	0.414	0.003

Supplementary Table 2. Pairwise comparison between all treatments on the field for bacterial and fungal taxa abundances and fungal functions. Two-way ANOVAs were used to discern how waste type, vegetation presence and their interaction influenced taxa relative abundance and fungal functions relative abundance. When a factor was revealed as a statistically significant predictor, a Tukey HSD *post hoc* pairwise comparison test was performed between all treatments.

**Bacteria**		*Sulfobacillaceae _g*		*Syntrophobacteraceae_g*		*Thermogemmatisporaceae_g*		*Xanthomonadaceae_g*	
	Waste type	< 0.001		< 0.001		0.033		0.018	
	Vegetation presence	< 0.001		< 0.001		0.031		0.154	
	Interaction	< 0.001		< 0.001		0.008		0.690	
**Pairwise comparison**									
Tailings	Unvegetated	0.0%	B	0.1%	B	0.0%	A	0.0%	C
	Vegetated	0.0%	B	0.6%	A	0.1%	A	0.3%	B
Waste rock	Unvegetated	6.3%	A	1.3%	A	1.1%	A	0.1%	BC
	Vegetated	0.0%	B	0.5%	AB	1.6%	A	1.6%	A
	p-value	< 0.001		< 0.001		0.020		< 0.001	
