# Supplementary material for: Plant Genotype Influences Physicochemical Properties of Substrate as Well as Bacterial and Fungal Assemblages in the Rhizosphere of Balsam Poplar
Source: Front Microbiol. 2020 Nov 23;11:575625. doi: 10.3389/fmicb.2020.575625 (PMC7719689; doi:10.3389/fmicb.2020.575625)
Supplement: Supplementary file 9 [file Table_3.PDF]

Supplementary Table 3. Spearman linear correlation analyses between bacterial and fungal taxa abundances and physicochemical properties of substrates in field samples. Weak correlations (>|0.3|) are highlighted in red; moderate correlations (>|0.5|) are highlighted in yellow; strong correlations (>|0.7|) are highlighted in green. CEC: Cation exchange capacity; BCSR: Base cation saturation ratio.

**Bacteria**	**C total**	**N total**	**S total**	**pH**	**P**	**K**	**Ca**	**Mg**	**Mn**	**Fe**	**Na**	**CEC**	**BCSR**
Acetobacteraceae_g	-0.38	-0.54	0.74	-0.55	0.21	-0.55	0.20	-0.52	-0.68	0.56	-0.49	0.18	0.06
Acidimicrobiales_f_g	-0.95	-0.91	0.14	0.15	-0.36	-0.75	-0.50	-0.89	-0.75	-0.08	-0.44	-0.37	-0.54
Acidiphilium	-0.26	-0.42	0.80	-0.80	0.30	-0.54	0.33	-0.53	-0.68	0.70	-0.36	0.46	0.15
Acidobacteriaceae_g	0.22	-0.01	0.89	-0.88	0.56	-0.30	0.67	-0.19	-0.46	0.77	-0.35	0.58	0.51
Actinomycetales_f_g	0.83	0.82	0.15	-0.38	0.48	0.54	0.37	0.57	0.44	0.32	0.37	0.24	0.38
[Pedosphaerales] auto67-4W_g	0.81	0.85	-0.22	0.09	0.37	0.75	0.23	0.74	0.71	-0.22	0.27	0.20	0.32
Bradyrhizobium	0.82	0.93	-0.47	0.25	0.11	0.86	0.12	0.85	0.90	-0.26	0.55	0.03	0.23
Burkholderia	0.80	0.79	-0.02	0.00	0.60	0.73	0.25	0.69	0.56	-0.20	0.09	0.20	0.27
Chloroflexi C0119_o_f_g	-0.37	-0.21	-0.61	0.71	-0.36	0.28	-0.75	0.07	0.24	-0.61	0.03	-0.56	-0.60
Candidatus Koribacter	-0.26	-0.04	-0.67	0.62	-0.48	0.22	-0.71	0.01	0.31	-0.52	0.20	-0.62	-0.57
Candidatus Nitrososphaera	-0.69	-0.53	-0.53	0.73	-0.70	-0.29	-0.74	-0.40	-0.17	-0.54	-0.05	-0.73	-0.63
Chitinophagaceae_g	0.58	0.64	-0.54	0.45	0.29	0.76	-0.01	0.69	0.71	-0.49	0.19	-0.05	0.10
Cytophagaceae_g	0.54	0.53	-0.36	0.22	0.42	0.76	-0.02	0.64	0.58	-0.36	0.00	0.10	0.07
[Chthoniobacteraceae] DA101	-0.14	0.09	-0.82	0.80	-0.62	0.31	-0.64	0.21	0.49	-0.65	0.38	-0.64	-0.46
Deinococcus	-0.66	-0.53	-0.57	0.73	-0.71	-0.27	-0.72	-0.38	-0.14	-0.51	-0.13	-0.72	-0.61
Acidobacteria iii1-8 DS-18_f_g	-0.24	-0.01	-0.69	0.70	-0.61	0.13	-0.69	0.00	0.28	-0.53	0.32	-0.65	-0.52
Thermoplasmata E2_f_g	-0.69	-0.53	-0.53	0.73	-0.70	-0.29	-0.74	-0.40	-0.17	-0.54	-0.05	-0.73	-0.63
Alphaproteobacteria Ellin329_f_g	0.89	0.96	-0.19	-0.05	0.30	0.75	0.41	0.81	0.77	-0.02	0.56	0.29	0.47
Betaproteobacteria Ellin6067_f_g	-0.08	0.11	-0.96	0.84	-0.52	0.41	-0.55	0.31	0.53	-0.67	0.39	-0.42	-0.41
Acidobacteria DA052 Ellin6513_f_g	0.90	0.97	-0.20	-0.08	0.31	0.78	0.29	0.80	0.79	0.00	0.54	0.20	0.36
Flavobacterium	0.71	0.74	-0.23	0.11	0.53	0.73	0.22	0.71	0.63	-0.35	0.20	0.20	0.28
Gaiellaceae_g	-0.19	0.03	-0.84	0.78	-0.54	0.33	-0.72	0.15	0.45	-0.59	0.31	-0.59	-0.56
Gammaproteobacteria_o_f_g	-0.27	-0.40	0.67	-0.59	0.18	-0.64	0.34	-0.54	-0.70	0.57	-0.31	0.29	0.14
Gemmata	-0.16	0.03	-0.76	0.86	-0.43	0.38	-0.62	0.24	0.48	-0.73	0.17	-0.47	-0.44
Gemmataceae_g	-0.54	-0.63	0.27	-0.36	-0.30	-0.76	-0.02	-0.71	-0.64	0.48	-0.24	-0.08	-0.13
Acidobacteria-6 iii1-15_f_g	0.54	0.70	-0.74	0.46	-0.11	0.75	-0.07	0.75	0.85	-0.39	0.68	-0.02	0.04
Isosphaeraceae_g	0.40	0.26	0.51	-0.80	0.14	-0.08	0.61	0.03	-0.05	0.81	0.05	0.44	0.51
AD3 JG37-AG-4_o_f_g	0.11	-0.05	0.82	-0.92	0.47	-0.36	0.57	-0.29	-0.47	0.79	-0.17	0.61	0.40
Kaistobacter	-0.52	-0.35	-0.46	0.77	-0.18	0.12	-0.78	-0.13	0.02	-0.73	-0.17	-0.55	-0.65
Koribacteraceae_g	0.87	0.94	-0.16	-0.12	0.33	0.72	0.35	0.75	0.70	0.02	0.54	0.24	0.41
Leptospirillum	-0.26	-0.46	0.78	-0.78	0.22	-0.63	0.33	-0.58	-0.74	0.68	-0.44	0.31	0.12
Methanomassiliicoccaceae_g	-0.40	-0.61	0.48	-0.51	-0.01	-0.62	0.05	-0.63	-0.71	0.54	-0.57	0.04	-0.14

Bacteria	C total	N total	S total	pH	P	K	Ca	Mg	Mn	Fe	Na	CEC	BCSR
Methylobacterium	-0.64	-0.48	-0.63	0.77	-0.53	0.00	-0.78	-0.18	0.02	-0.63	0.01	-0.49	-0.68
Methylocystaceae_g	0.68	0.84	-0.25	0.12	0.16	0.70	-0.02	0.62	0.63	-0.17	0.50	-0.16	0.10
Mycobacterium	0.74	0.73	0.13	-0.21	0.78	0.77	0.26	0.68	0.48	-0.02	0.13	0.34	0.26
Oxalobacteraceae_g	-0.27	-0.07	-0.74	0.88	-0.46	0.24	-0.65	0.11	0.37	-0.85	0.14	-0.55	-0.48
Planctomyces	0.22	0.30	-0.61	0.53	0.11	0.61	-0.37	0.42	0.52	-0.55	0.01	-0.17	-0.27
Proteobacteria_c_o_f_g	-0.55	-0.66	0.56	-0.51	-0.06	-0.75	0.06	-0.73	-0.86	0.51	-0.31	0.10	-0.13
Rhodoplanes	0.62	0.74	-0.52	0.34	0.08	0.81	-0.10	0.74	0.87	-0.42	0.48	0.02	0.03
Rhodospirillaceae_g	0.79	0.84	-0.38	0.25	0.25	0.83	0.19	0.83	0.75	-0.32	0.36	0.12	0.28
Rubrivivax	-0.14	0.07	-0.77	0.83	-0.55	0.27	-0.61	0.18	0.37	-0.75	0.24	-0.75	-0.42
Sinobacteraceae_g	0.81	0.74	0.27	-0.59	0.26	0.33	0.76	0.58	0.42	0.60	0.47	0.57	0.74
Solibacterales_f_g	0.68	0.79	-0.34	0.22	0.32	0.81	0.00	0.73	0.72	-0.27	0.42	0.00	0.12
Solirubrobacterales_f_g	-0.62	-0.52	-0.28	0.52	-0.36	-0.29	-0.64	-0.42	-0.28	-0.34	-0.17	-0.52	-0.64
Sphingobacteriaceae_g	0.65	0.71	-0.22	0.18	0.54	0.86	0.06	0.74	0.60	-0.34	0.15	0.09	0.14
Sphingobacteriales_f_g	0.50	0.61	-0.40	0.38	0.38	0.87	-0.11	0.74	0.65	-0.47	0.31	0.05	0.01
Sphingomonas	0.49	0.54	-0.45	0.41	0.42	0.76	-0.12	0.62	0.63	-0.50	0.05	-0.02	-0.01
Sulfobacillaceae_g	-0.27	-0.46	0.79	-0.81	0.24	-0.64	0.43	-0.52	-0.72	0.71	-0.36	0.41	0.25
Syntrophobacteraceae_g	0.29	0.12	0.49	-0.67	0.45	-0.09	0.37	-0.03	-0.24	0.54	-0.06	0.32	0.19
Thermogemmatisporaceae_g	0.37	0.23	0.47	-0.52	0.71	0.10	0.14	-0.03	-0.17	0.40	-0.34	0.16	-0.01
Xanthomonadaceae_g	0.64	0.59	0.13	-0.12	0.78	0.59	0.16	0.46	0.28	-0.13	-0.14	0.14	0.13

Supplementary Table 3. Spearman linear correlation analyses between bacterial and fungal taxa abundances and physicochemical properties of substrates in field samples. Weak correlations (>|0.3|) are highlighted in red; moderate correlations (>|0.5|) are highlighted in yellow; strong correlations (>|0.7|) are highlighted in green. CEC: Cation exchange capacity; BCSR: Base cation saturation ratio.

**Fungi**	**C total**	**N total**	**S total**	**pH**	**P**	**K**	**Ca**	**Mg**	**Mn**	**Fe**	**Na**	**CEC**	**BCSR**
Acidea	-0.31	-0.39	0.42	-0.23	0.02	-0.39	-0.08	-0.50	-0.37	0.01	-0.66	-0.21	-0.09
Agaricales_f_g	0.38	0.40	-0.31	0.24	-0.31	0.03	0.31	0.31	0.31	-0.31	0.17	-0.03	0.38
Alternaria	-0.56	-0.48	-0.29	0.47	-0.45	-0.30	-0.61	-0.48	-0.32	-0.52	-0.43	-0.73	-0.60
Amphinema	-0.91	-0.86	0.02	0.20	-0.57	-0.72	-0.43	-0.79	-0.67	-0.16	-0.28	-0.39	-0.45
Apiotrichum	0.32	0.29	0.04	-0.02	0.04	0.16	0.41	0.34	0.19	0.02	-0.14	0.05	0.47
Ascomycota_o_c_f_g	0.28	0.48	-0.63	0.40	-0.45	0.30	-0.01	0.41	0.40	-0.23	0.71	-0.16	0.09
Cenococcum	0.14	0.34	-0.63	0.44	-0.66	0.18	-0.29	0.22	0.50	-0.30	0.59	-0.39	-0.14
Chaetosphaeriaceae_g	-0.63	-0.53	-0.19	0.40	-0.68	-0.34	-0.59	-0.47	-0.29	-0.37	-0.15	-0.57	-0.53
Chaetothyriales_g	-0.77	-0.76	0.09	0.06	-0.43	-0.69	-0.39	-0.84	-0.75	-0.01	-0.34	-0.32	-0.50
Cistella	-0.68	-0.59	-0.18	0.34	-0.62	-0.39	-0.59	-0.57	-0.34	-0.28	-0.07	-0.35	-0.58
Cladophialophora	-0.42	-0.59	0.65	-0.60	0.23	-0.75	0.14	-0.80	-0.70	0.49	-0.59	0.18	-0.04
Cladosporium	-0.72	-0.62	-0.14	0.23	-0.49	-0.47	-0.56	-0.71	-0.55	-0.32	-0.29	-0.51	-0.62
Clavulinopsis	0.54	0.70	-0.67	0.33	-0.44	0.43	0.07	0.63	0.65	-0.21	0.80	-0.13	0.22
Cortinarius	0.68	0.78	-0.44	0.29	0.22	0.93	-0.16	0.80	0.81	-0.24	0.46	-0.05	-0.04
Cryptococcus	0.09	0.17	-0.27	0.32	0.09	0.35	-0.15	0.33	0.25	-0.53	0.01	-0.15	-0.07
Exophiala	-0.69	-0.49	-0.29	0.52	-0.39	-0.06	-0.66	-0.31	-0.19	-0.60	-0.13	-0.51	-0.55
Ganoderma	-0.27	-0.20	-0.46	0.65	-0.52	-0.08	-0.59	-0.17	0.01	-0.56	-0.14	-0.68	-0.57
Geomyces	-0.55	-0.52	-0.09	0.25	-0.56	-0.57	-0.37	-0.64	-0.50	-0.14	-0.24	-0.49	-0.41
Hebeloma	0.21	0.17	0.13	0.16	0.51	0.39	0.07	0.31	0.20	-0.10	-0.17	0.24	0.07
Inocybe	0.02	0.08	-0.37	0.15	-0.24	0.01	0.11	0.25	0.11	-0.03	0.55	0.13	0.14
Knufia	0.34	0.24	0.03	-0.13	0.35	0.39	-0.18	0.21	0.11	0.01	-0.13	-0.07	-0.24
Lecanoromycetes_g	0.70	0.79	-0.54	0.13	-0.20	0.47	0.20	0.66	0.76	0.01	0.72	0.07	0.31
Leotiomycetes_c_f_g	0.01	0.07	-0.27	0.06	-0.54	-0.02	-0.14	-0.02	0.17	0.06	0.21	-0.21	-0.07
Leptodontidium	0.18	0.05	0.18	-0.39	-0.26	-0.28	0.53	0.04	0.05	0.46	0.18	0.51	0.49
Lipomyces	-0.10	-0.38	0.46	-0.52	0.26	-0.41	0.13	-0.28	-0.45	0.52	-0.28	0.23	-0.06
Meliniomyces	0.59	0.51	0.13	-0.17	0.39	0.22	0.52	0.48	0.34	0.06	-0.10	0.08	0.57
Oidiodendron	0.29	0.31	-0.18	0.17	0.16	0.25	0.07	0.42	0.41	-0.16	0.19	-0.04	0.16
Parmelia	-0.24	-0.02	-0.53	0.36	-0.58	0.03	-0.49	-0.09	0.17	-0.21	0.45	-0.42	-0.38
Penicillium	0.67	0.51	0.19	-0.22	0.59	0.56	0.26	0.59	0.42	0.18	0.07	0.42	0.22
Pezoloma	-0.44	-0.54	0.48	-0.44	0.10	-0.60	0.11	-0.56	-0.54	0.27	-0.46	-0.01	0.03
Phialocephala	-0.43	-0.41	0.16	-0.12	-0.44	-0.67	0.02	-0.59	-0.41	0.10	-0.09	-0.09	0.02
Piloderma	0.27	0.45	-0.68	0.36	-0.58	0.23	-0.23	0.27	0.51	-0.22	0.64	-0.34	-0.11

Fungi	C total	N total	S total	pH	P	K	Ca	Mg	Mn	Fe	Na	CEC	BCSR
Pleosporales_fam_Incertae_sedis_g	-0.54	-0.56	0.03	-0.09	-0.16	-0.33	-0.39	-0.54	-0.64	0.05	-0.27	-0.26	-0.52
Pyrenopeziza	-0.47	-0.58	0.62	-0.45	0.02	-0.59	0.04	-0.68	-0.61	0.24	-0.63	-0.02	-0.03
Rozellomycota_g	0.81	0.88	-0.39	0.18	0.23	0.75	0.19	0.78	0.84	-0.22	0.42	0.18	0.29
Sagenomella	-0.07	-0.20	0.17	-0.28	0.15	-0.18	0.12	-0.05	-0.26	0.35	-0.13	0.05	0.04
Setophoma	-0.56	-0.50	-0.03	0.11	-0.35	-0.67	-0.21	-0.72	-0.49	-0.08	-0.18	-0.22	-0.27
Sistotrema	0.10	0.11	0.13	-0.11	0.56	0.45	-0.20	0.14	0.09	-0.16	-0.23	0.02	-0.20
Sordariales_g	-0.29	-0.11	-0.56	0.43	-0.28	0.09	-0.51	-0.08	0.05	-0.42	0.12	-0.45	-0.47
Talaromyces	-0.14	-0.38	0.49	-0.45	-0.04	-0.49	0.12	-0.51	-0.51	0.45	-0.52	0.12	-0.05
Teratosphaeriaceae_g	-0.29	-0.48	0.64	-0.59	0.04	-0.68	0.30	-0.70	-0.70	0.59	-0.47	0.33	0.10
Tetracladium	-0.69	-0.59	-0.09	0.30	-0.48	-0.52	-0.47	-0.70	-0.42	-0.21	-0.19	-0.33	-0.49
Tomentella	-0.16	-0.25	0.20	-0.08	0.22	-0.11	-0.44	-0.38	-0.27	-0.17	-0.65	-0.51	-0.51
Tremella	-0.61	-0.67	0.33	-0.15	-0.17	-0.71	-0.22	-0.85	-0.70	0.14	-0.47	-0.18	-0.35
Tricholoma	-0.03	0.13	-0.39	0.14	-0.42	0.06	-0.25	0.01	0.21	0.07	0.54	-0.20	-0.17
Umbelopsis	-0.26	-0.24	-0.06	0.08	-0.05	-0.41	0.05	-0.24	-0.12	0.08	0.12	0.10	0.02
Venturia	-0.15	-0.39	0.67	-0.50	0.30	-0.52	0.32	-0.44	-0.46	0.38	-0.54	0.41	0.18
