# Supplementary material for: Plant Genotype Influences Physicochemical Properties of Substrate as Well as Bacterial and Fungal Assemblages in the Rhizosphere of Balsam Poplar
Source: Front Microbiol. 2020 Nov 23;11:575625. doi: 10.3389/fmicb.2020.575625 (PMC7719689; doi:10.3389/fmicb.2020.575625)
Supplement: Supplementary file 11 [file Table_5.PDF]

Supplementary Table 5. Other physicochemical properties of substrates after the greenhouse experiment. See Table 2 for the other parameters. CEC: Cation exchange capacity; BCSR: Base cation saturation ratio.

	**Mn (mmol/kg)**			**Na (mmol/kg)**			**CEC**			**BCSR**		
	**Control**	**Tailings**	**Waste rock**	**Control**	**Tailings**	**Waste rock**	**Control**	**Tailings**	**Waste rock**	**Control**	**Tailings**	**Waste rock**
**W08**	0.84 b	0.256	0.144	5.70 b	0.59 b	1.98 bc	16.4 c	4.0 b	12.5	15.7 abcd	55.0 d	38.9 cd
**W09**	2.79 a	0.432	0.177	16.10 a	2.47 a	3.90 a	43.9 a	7.6 a	16.0	18.0 abc	57.5 bcd	48.6 ab
**W10**	2.25 ab	0.495	0.214	14.30 ab	2.54 a	2.90 abc	42.4 a	8.8 a	15.4	9.0 bcd	61.6 bcd	45.9 abc
**W13**	2.45 a	0.514	0.185	12.70 ab	2.50 a	4.00 ab	38.7 abc	8.3 a	15.7	25.7 a	62.8 abcd	53.3 a
**N16**	1.94 ab	0.605	0.116	11.30 ab	2.42 ab	2.28 abc	34.3 abc	9.5 a	13.7	23.5 a	71.1 a	45.7 abc
**C21**	1.73 ab	0.702	0.163	12.80 ab	2.19 ab	1.59 c	39.1 abc	9.2 a	11.4	8.7 cd	64.6 ab	35.4 d
**C23**	1.77 ab	0.456	0.241	9.20 b	1.57 ab	3.66 abc	31.2 bc	8.2 a	15.4	20.0 ab	59.9 bcd	50.6 a
**C25**	2.23 ab	0.623	0.162	11.80 ab	2.91 a	2.64 abc	41.8 a	8.8 a	13.5	6.3 d	63.9 abc	42.0 bcd
**C29**	1.98 ab	0.597	0.185	11.50 ab	1.99 ab	3.13 abc	35.5 abc	9.3 a	16.4	18.0 abc	60.9 bcd	51.9 a
**N33**	2.33 ab	0.563	0.202	16.50 a	2.20 ab	2.44 abc	41.2 ab	8.6 a	13.6	8.0 cd	56.5 cd	46.6 ab
**p-value**	0.023	0.086	0.326	0.007	0.007	0.005	< 0.001	< 0.001	0.021	< 0.001	0.007	< 0.001
