# Supplementary material for: Plant Genotype Influences Physicochemical Properties of Substrate as Well as Bacterial and Fungal Assemblages in the Rhizosphere of Balsam Poplar
Source: Front Microbiol. 2020 Nov 23;11:575625. doi: 10.3389/fmicb.2020.575625 (PMC7719689; doi:10.3389/fmicb.2020.575625)
Supplement: Supplementary file 12 [file Table_6.PDF]

Supplementary Table 6. Spearman linear correlation analyses between physicochemical properties of substrates and tree growth measurements in the greenhouse experiment. Weak correlations (>|0.3|) are highlighted in red; moderate correlations (>|0.5|) are highlighted in yellow; strong correlations (>|0.7|) are highlighted in green. CEC: Cation exchange capacity; BCSR: Base cation saturation ratio.

		Chlorophyll content				
	**Growth**	**Season 1**	**Season 2**	**Shoot diameter**	**Biomass**	**Blooming**
**C total**	0.04	0.07	-0.01	0.14	0.22	-0.04
**N total**	0.00	0.05	0.05	0.17	0.32	-0.04
**S total**	-0.17	-0.03	0.21	-0.07	0.00	-0.06
**pH**	0.13	-0.18	-0.29	-0.05	-0.18	-0.06
**P**	0.00	0.05	0.00	0.05	0.13	0.03
**K**	0.19	0.08	-0.13	0.08	0.10	-0.06
**Ca**	0.05	0.05	-0.06	0.03	0.24	-0.04
**Mg**	0.18	0.11	-0.29	0.09	0.15	-0.11
**Mn**	0.14	0.12	-0.24	0.23	0.19	0.01
**Fe**	-0.15	0.00	0.27	-0.23	-0.01	0.13
**Na**	0.01	0.10	-0.08	0.16	0.39	-0.07
**CEC**	0.01	0.07	0.01	0.06	0.28	-0.05
**BCSR**	0.16	0.08	-0.27	0.15	0.09	-0.06
