# Supplementary material for: Plant Genotype Influences Physicochemical Properties of Substrate as Well as Bacterial and Fungal Assemblages in the Rhizosphere of Balsam Poplar
Source: Front Microbiol. 2020 Nov 23;11:575625. doi: 10.3389/fmicb.2020.575625 (PMC7719689; doi:10.3389/fmicb.2020.575625)
Supplement: Supplementary file 13 [file Table_7.PDF]

Supplementary Table 7. Pairwise comparison between genotypes in each substrate type in the greenhouse experiment for fungal functions relative abundance. Two-way ANOVAs were used to discern how substrate type, genotype and their interaction influenced fungal functions relative abundance. Additional analyses were performed for each substrate type separately and between substrate types to better assess the indivudial effects of genotype and substrate type.

**Anova**		Ectomycorrhizae	Saprotroph	Plant pathogen	Ericoid mycorrhizae	Brown rot
	Genotype	0.718	0.896	0.171	0.144	0.071
	Substrate type	< 0.001	< 0.001	0.029	< 0.001	0.310
	Interaction	0.093	0.450	0.594	0.099	0.558
**Pairwise comparison between substrate types**						
	Control	41.3% A	24.3% A	6.7% A	0.1% B	0.4% A
	Tailings	28.4% B	19.1% B	4.7% B	0.1% B	0.3% A
	Waste rock	49.6% A	23.6% A	5.0% AB	2.8% A	0.5% A
	p-value	< 0.001	< 0.001	0.019	0.001	0.245
**Pairwise comparison by substrate type**						
Control	W08	36.8% A	19.4% A	5.4% A	0.2% A	0.5% A
	W09	37.8% A	23.3% A	7.2% A	0.2% A	0.4% A
	W10	37.0% A	26.0% A	7.0% A	0.3% A	0.2% A
	W13	36.8% A	26.2% A	8.4% A	0.2% A	0.1% A
	N16	55.9% A	24.6% A	6.7% A	0.1% A	0.0% A
	C21	49.8% A	24.1% A	3.3% A	0.0% A	0.5% A
	C23	33.9% A	23.9% A	7.4% A	0.2% A	0.5% A
	C25	35.3% A	27.9% A	7.8% A	0.1% A	0.4% A
	C29	58.7% A	19.8% A	4.7% A	0.1% A	0.0% A
	N33	38.5% A	27.5% A	8.1% A	0.1% A	0.9% A
	p-value	0.390	0.686	0.207	0.362	0.063
Tailngs	W08	24.0% A	22.3% A	3.5% A	0.1% A	0.7% A
	W09	34.8% A	17.2% A	6.7% A	0.0% A	0.1% A
	W10	8.4% A	21.9% A	7.3% A	0.7% A	0.2% A
	W13	38.9% A	17.0% A	4.5% A	0.0% A	0.2% A
	N16	26.8% A	17.3% A	9.9% A	0.1% A	0.1% A
	C21	22.0% A	26.2% A	3.3% A	0.0% A	0.3% A
	C23	32.2% A	17.6% A	4.6% A	0.0% A	0.2% A
	C25	28.7% A	14.5% A	3.4% A	0.0% A	0.1% A
	C29	40.6% A	19.5% A	2.6% A	0.0% A	0.2% A
	N33	25.6% A	17.2% A	2.9% A	0.0% A	0.7% A
	p-value	0.148	0.229	0.516	0.414	0.730
Waste rock	W08	53.3% A	18.8% A	4.5% A	2.7% AB	0.5% A
	W09	45.1% A	27.1% A	6.8% A	0.8% B	0.3% A
	W10	63.4% A	21.8% A	2.4% A	3.4% AB	0.1% A
	W13	52.7% A	25.7% A	6.6% A	0.3% AB	0.9% A
	N16	57.5% A	25.3% A	2.4% A	2.7% AB	0.2% A
	C21	57.5% A	21.1% A	1.3% A	2.6% AB	0.2% A
	C23	40.4% A	27.9% A	5.9% A	2.5% AB	0.8% A
	C25	55.3% A	23.5% A	6.0% A	0.5% B	1.1% A
	C29	35.2% A	21.1% A	8.7% A	0.5% B	0.7% A
	N33	41.7% A	24.8% A	4.7% A	9.7% A	0.4% A
	p-value	0.380	0.907	0.484	0.005	0.348

Supplementary Table 7. Pairwise comparison between genotypes in each substrate type in the greenhouse experiment for fungal functions relative abundance. Two-way ANOVAs were used to discern how substrate type, genotype and their interaction influenced fungal functions relative abundance. Additional analyses were performed for each substrate type separately and between substrate types to better assess the indivudial effects of genotype and substrate type.

		White rot		Lichenized mycorrhizae		Arbuscular mycorrhizae	
**Anova**							
	Genotype	0.472		0.063		0.696	
	Substrate type	< 0.001		0.997		0.384	
	Interaction	0.885		0.270		0.931	
**Pairwise comparison between substrate types**							
	Control	0.2%	A	0.0%	A	0.0%	A
	Tailings	0.0%	B	0.0%	A	0.0%	A
	Waste rock	0.1%	B	0.0%	A	0.0%	A
	p-value	< 0.001		0.998		0.281	
**Pairwise comparison by substrate type**							
Control	W08	0.2%	A	0.0%	A	0.0%	A
	W09	0.3%	A	0.0%	A	0.0%	A
	W10	0.2%	A	0.0%	A	0.0%	A
	W13	0.1%	A	0.0%	A	0.0%	A
	N16	0.2%	A	0.0%	A	0.0%	A
	C21	0.1%	A	0.0%	A	0.0%	A
	C23	0.2%	A	0.0%	A	0.0%	A
	C25	0.2%	A	0.0%	A	0.0%	A
	C29	0.1%	A	0.0%	A	0.0%	A
	N33	0.2%	A	0.0%	A	0.0%	A
	p-value	0.717		0.622		0.999	
Tailngs	W08	0.0%	A	0.0%	A	0.0%	A
	W09	0.0%	A	0.0%	A	0.0%	A
	W10	0.1%	A	0.0%	A	0.0%	A
	W13	0.1%	A	0.0%	A	0.0%	A
	N16	0.0%	A	0.0%	A	0.0%	A
	C21	0.0%	A	0.0%	A	0.0%	A
	C23	0.1%	A	0.0%	A	0.0%	A
	C25	0.0%	A	0.0%	A	0.0%	A
	C29	0.0%	A	0.0%	A	0.0%	A
	N33	0.1%	A	0.0%	A	0.0%	A
	p-value	0.668		0.049		0.781	
Waste rock	W08	0.1%	A	0.0%	A	0.0%	A
	W09	0.2%	A	0.0%	A	0.0%	A
	W10	0.1%	A	0.0%	A	0.0%	A
	W13	0.1%	A	0.0%	A	0.0%	A
	N16	0.1%	A	0.0%	A	0.0%	A
	C21	0.0%	A	0.0%	A	0.0%	A
	C23	0.1%	A	0.0%	A	0.0%	A
	C25	0.0%	A	0.0%	A	0.0%	A
	C29	0.1%	A	0.0%	A	0.0%	A
	N33	0.1%	A	0.0%	A	0.0%	A
	p-value	0.645		0.150		0.999	
