# Supplementary material for: Plant Genotype Influences Physicochemical Properties of Substrate as Well as Bacterial and Fungal Assemblages in the Rhizosphere of Balsam Poplar
Source: Front Microbiol. 2020 Nov 23;11:575625. doi: 10.3389/fmicb.2020.575625 (PMC7719689; doi:10.3389/fmicb.2020.575625)
Supplement: Supplementary file 14 [file Table_8.PDF]

Supplementary Table 8. Pairwise comparison between genotypes in each substrate type in the greenhouse experiment for bacterial and fungal taxa relative abundance. Two-way ANOVAs were used to discern how substrate type, genotype and their interaction influenced taxa relative abundance. Additional analyses were performed for each substrate type separately and between substrate types to better assess the indivudial effects of genotype and substrate type.

**Bacteria**		*Anaerolineae SBR1031 A4b_g*	*Acidobacteriaceae_g*	*Bradyrhizobium*	*Caulobacteraceae_g*
	Genotype	0.004	0.694	0.013	0.168
	Substrate type	< 0.001	< 0.001	< 0.001	< 0.001
	Interaction	0.307	0.707	0.011	0.124
**Pairwise comparison between substrate types**					
	Control	0.8% B	0.1% B	0.4% C	0.5% B
	Tailings	1.4% A	0.0% C	0.9% B	0.5% B
	Waste rock	0.7% B	1.9% A	1.2% A	1.8% A
	p-value	< 0.001	< 0.001	< 0.001	< 0.001
**Pairwise comparison by substrate type**					
Control	W08	0.9% A	0.2% A	0.4% AB	0.5% A
	W09	0.6% A	0.1% A	0.5% AB	0.5% A
	W10	0.6% A	0.1% A	0.3% B	0.5% A
	W13	0.7% A	0.1% A	0.4% AB	0.4% A
	N16	0.9% A	0.0% A	0.3% AB	0.6% A
	C21	0.7% A	0.1% A	0.7% A	0.8% A
	C23	1.1% A	0.2% A	0.4% AB	0.5% A
	C25	0.7% A	0.1% A	0.4% AB	0.6% A
	C29	0.7% A	0.1% A	0.5% AB	0.6% A
	N33	0.8% A	0.1% A	0.4% AB	0.5% A
	p-value	0.321	0.557	0.011	0.503
Tailngs	W08	1.4% A	0.0% A	0.6% A	0.4% A
	W09	1.6% A	0.0% A	1.3% A	0.6% A
	W10	1.3% A	0.1% A	0.9% A	0.5% A
	W13	1.4% A	0.0% A	0.9% A	0.5% A
	N16	1.3% A	0.0% A	0.6% A	0.3% A
	C21	1.0% A	0.0% A	0.8% A	0.4% A
	C23	1.5% A	0.0% A	1.5% A	0.6% A
	C25	1.3% A	0.0% A	0.5% A	0.8% A
	C29	1.5% A	0.0% A	1.0% A	0.5% A
	N33	1.5% A	0.1% A	1.0% A	0.5% A
	p-value	0.904	0.667	0.018	0.047
Waste rock	W08	0.4% B	2.5% A	1.5% A	1.7% A
	W09	0.9% AB	1.4% A	1.3% A	1.5% A
	W10	0.5% AB	1.6% A	0.9% A	2.2% A
	W13	0.6% AB	1.5% A	1.3% A	1.4% A
	N16	0.8% AB	2.2% A	1.5% A	2.4% A
	C21	0.3% B	2.5% A	1.3% A	2.4% A
	C23	1.4% A	1.3% A	1.2% A	1.3% A
	C25	0.4% B	2.6% A	0.8% A	1.8% A
	C29	1.1% AB	1.3% A	1.0% A	1.4% A
	N33	0.5% B	1.8% A	1.1% A	1.7% A
	p-value	< 0.001	0.711	0.123	0.188

Supplementary Table 8. Pairwise comparison between genotypes in each substrate type in the greenhouse experiment for bacterial and fungal taxa relative abundance. Two-way ANOVAs were used to discern how substrate type, genotype and their interaction influenced taxa relative abundance. Additional analyses were performed for each substrate type separately and between substrate types to better assess the indivudial effects of genotype and substrate type.

**Bacteria**		*Chitinophagaceae_g*	*Acidimicrobiales EB1017_g*	*Alphaproteobacteria Ellin329_f_g*	*Betaproteobacteria Ellin6067_f_g*
	Genotype	0.011	0.690	0.022	0.203
	Substrate type	< 0.001	< 0.001	< 0.001	< 0.001
	Interaction	0.261	0.245	0.451	0.026
**Pairwise comparison between substrate types**					
	Control	22.4% A	0.9% B	6.1% B	2.0% A
	Tailings	11.9% B	1.1% A	3.6% C	1.6% B
	Waste rock	8.2% C	0.4% C	8.7% A	2.1% A
	p-value	< 0.001	< 0.001	< 0.001	< 0.001
**Pairwise comparison by substrate type**					
Control	W08	23.9% A	0.8% A	5.2% A	2.3% A
	W09	25.9% A	0.9% A	6.2% A	2.2% A
	W10	20.8% A	0.6% A	7.2% A	2.5% A
	W13	26.0% A	0.6% A	4.6% A	1.4% A
	N16	25.2% A	1.1% A	6.3% A	1.7% A
	C21	17.3% A	0.9% A	7.0% A	2.1% A
	C23	19.0% A	0.8% A	6.3% A	2.3% A
	C25	24.1% A	0.9% A	6.3% A	2.3% A
	C29	18.2% A	0.9% A	5.6% A	1.8% A
	N33	22.7% A	1.0% A	6.8% A	1.8% A
	p-value	0.069	0.710	0.823	0.217
Tailngs	W08	13.9% A	1.1% A	2.7% AB	1.5% A
	W09	13.4% A	1.3% A	2.9% AB	1.9% A
	W10	11.1% A	1.4% A	4.1% AB	1.4% A
	W13	12.5% A	1.5% A	3.8% AB	1.3% A
	N16	8.9% A	1.3% A	3.3% AB	2.1% A
	C21	10.9% A	0.7% A	4.6% AB	2.0% A
	C23	11.2% A	1.1% A	2.3% B	1.3% A
	C25	14.4% A	0.9% A	5.3% A	1.5% A
	C29	9.0% A	0.9% A	2.6% AB	1.6% A
	N33	12.6% A	1.1% A	4.4% AB	1.8% A
	p-value	0.350	0.128	0.002	0.030
Waste rock	W08	9.3% A	0.5% A	11.2% A	1.8% A
	W09	11.1% A	0.5% A	8.1% A	2.3% A
	W10	6.7% A	0.5% A	9.4% A	1.7% A
	W13	9.4% A	0.4% A	8.5% A	2.2% A
	N16	7.0% A	0.3% A	7.6% A	2.6% A
	C21	3.2% A	0.2% A	7.6% A	1.5% A
	C23	8.6% A	0.4% A	9.4% A	2.6% A
	C25	7.3% A	0.2% A	7.5% A	2.3% A
	C29	11.9% A	0.5% A	8.1% A	2.0% A
	N33	6.2% A	0.2% A	8.8% A	1.8% A
	p-value	0.051	0.527	0.719	0.155

Supplementary Table 8. Pairwise comparison between genotypes in each substrate type in the greenhouse experiment for bacterial and fungal taxa relative abundance. Two-way ANOVAs were used to discern how substrate type, genotype and their interaction influenced taxa relative abundance. Additional analyses were performed for each substrate type separately and between substrate types to better assess the indivudial effects of genotype and substrate type.

**Bacteria**		*Frankiaceae_g*	*Gaiellaceae_g*	*Gemmataceae_g*	*Geobacter*	*Acidobacteria-6 iii1_15_f_g*
	Genotype	0.008	0.339	< 0.001	< 0.001	0.608
	Substrate type	< 0.001	< 0.001	< 0.001	< 0.001	< 0.001
	Interaction	0.081	0.001	0.002	0.006	0.057
**Pairwise comparison between substrate types**						
	Control	1.4% A	0.8% B	0.8% B	0.2% B	0.9% B
	Tailings	0.8% C	0.6% C	3.7% A	5.6% A	2.2% A
	Waste rock	1.0% B	1.2% A	1.5% C	0.2% B	0.6% C
	p-value	< 0.001	< 0.001	< 0.001	< 0.001	< 0.001
**Pairwise comparison by substrate type**						
Control	W08	1.4% A	1.0% A	1.4% A	0.0% A	1.0% A
	W09	1.2% A	0.7% A	0.7% A	0,0% A	0.8% A
	W10	1.7% A	0.7% A	0.6% A	0.1% A	1.1% A
	W13	1.4% A	0.6% A	0.9% A	0.0% A	1.1% A
	N16	1.3% A	0.6% A	1.1% A	0,0% A	0.9% A
	C21	1.6% A	1.0% A	0.6% A	0,0% A	0.9% A
	C23	1.4% A	0.9% A	0.8% A	0.6% A	0.7% A
	C25	1.4% A	0.9% A	0.8% A	0.7% A	0.9% A
	C29	1.4% A	0.6% A	0.6% A	0.1% A	0.9% A
	N33	1.4% A	0.9% A	0.8% A	0.2% A	1.0% A
	p-value	0.844	0.495	0.205	0.669	0.805
Tailngs	W08	0.9% AB	0.6% A	4.8% A	4.5% ABC	2.5% AB
	W09	0.8% AB	0.8% A	2.7% CD	1.9% ABC	1.8% AB
	W10	0.7% AB	0.7% A	4.2% ABC	10.8% A	2.2% AB
	W13	0.5% B	0.6% A	4.3% AB	2.6% ABC	3.0% A
	N16	1.5% A	0.3% A	5.0% A	0.0% C	2.8% AB
	C21	0.8% AB	0.4% A	3.4% ABCD	6.7% ABC	2.4% AB
	C23	0.4% B	0.5% A	3.1% BCD	10.8% A	1.4% B
	C25	1.1% AB	0.5% A	4.0% ABCD	1.3% BC	2.0% AB
	C29	0.4% B	0.4% A	2.4% D	9.0% AB	2.1% AB
	N33	0.9% AB	0.7% A	3.6% ABCD	4.7% ABC	2.3% AB
	p-value	< 0.001	0.004	< 0.001	< 0.001	0.020
Waste rock	W08	1.2% A	1.1% AB	1.2% A	0.0% B	0.5% A
	W09	1.2% A	1.2% AB	1.9% A	0.1% B	0.8% A
	W10	0.9% A	1.0% AB	1.4% A	0.1% B	0.7% A
	W13	1.1% A	1.2% AB	1.5% A	0.4% B	0.4% A
	N16	1.2% A	1.6% A	1.7% A	0.0% B	0.9% A
	C21	1.0% A	1.4% AB	1.1% A	0.0% B	0.3% A
	C23	0.8% A	1.2% AB	1.8% A	1.5% A	0.8% A
	C25	0.8% A	1.3% AB	1.3% A	0.0% B	0.7% A
	C29	0.9% A	1.1% AB	1.3% A	0.3% B	0.9% A
	N33	0.9% A	0.9% B	1.5% A	0.1% B	0.6% A
	p-value	0.781	0.017	0.955	< 0.001	0.303

Supplementary Table 8. Pairwise comparison between genotypes in each substrate type in the greenhouse experiment for bacterial and fungal taxa relative abundance. Two-way ANOVAs were used to discern how substrate type, genotype and their interaction influenced taxa relative abundance. Additional analyses were performed for each substrate type separately and between substrate types to better assess the indivudial effects of genotype and substrate type.

**Bacteria**		*Isosphaeraceae_g*	*Myxococcales_f_g*	*Opitutaceae_g*	*Opitutus*	*Pedosphaerales_f_g*
	Genotype	0.002	0.003	0.281	0.002	0.001
	Substrate type	< 0.001	< 0.001	< 0.001	< 0.001	< 0.001
	Interaction	0.001	0.567	0.328	< 0.001	< 0.001
**Pairwise comparison between substrate types**						
	Control	0.4% B	3.3% A	0.4% B	0.8% B	0.7% B
	Tailings	0.3% B	2.4% B	2.8% A	1.2% A	0.7% B
	Waste rock	1.8% A	1.5% C	0.4% B	0.6% B	1.3% A
	p-value	< 0.001	< 0.001	< 0.001	< 0.001	< 0.001
**Pairwise comparison by substrate type**						
Control	W08	0.6% A	3.8% A	0.4% A	0.7% A	0.7% AB
	W09	0.4% AB	2.8% A	0.3% A	0.7% A	0.4% B
	W10	0.3% AB	3.3% A	0.3% A	0.8% A	0.6% B
	W13	0.2% B	4.7% A	0.5% A	0.5% A	0.4% B
	N16	0.4% AB	3.3% A	0.5% A	1.0% A	0.6% AB
	C21	0.4% AB	1.9% A	0.3% A	0.5% A	1.4% A
	C23	0.5% AB	3.0% A	0.8% A	1.0% A	1.1% AB
	C25	0.3% B	3.1% A	0.4% A	0.7% A	0.8% AB
	C29	0.2% B	3.5% A	0.2% A	0.7% A	0.8% AB
	N33	0.4% AB	2.9% A	0.2% A	0.9% A	0.8% AB
	p-value	< 0.001	0.342	0.419	0.475	< 0.001
Tailngs	W08	0.3% A	2.7% A	3.9% A	1.0% B	0.7% A
	W09	0.6% A	2.4% A	1.3% A	0.9% B	0.6% A
	W10	0.4% A	2.4% A	3.4% A	1.2% B	0.6% A
	W13	0.3% A	2.7% A	4.5% A	1.1% B	0.7% A
	N16	0.4% A	3.7% A	4.5% A	2.7% A	0.5% A
	C21	0.5% A	2.1% A	1.9% A	1.2% B	0.7% A
	C23	0.2% A	1.7% A	1.6% A	0.9% B	1.0% A
	C25	0.3% A	2.3% A	1.6% A	0.7% B	0.5% A
	C29	0.2% A	2.1% A	2.8% A	1.2% B	1.1% A
	N33	0.3% A	2.8% A	3.1% A	1.2% B	1.0% A
	p-value	0.055	0.098	0.107	< 0.001	0.051
Waste rock	W08	2.5% A	1.4% A	0.4% A	0.7% A	2.4% AB
	W09	1.4% A	1.4% A	0.4% A	0.5% A	1.1% ABC
	W10	1.1% A	1.1% A	0.2% A	0.6% A	1.2% ABC
	W13	1.0% A	1.7% A	0.3% A	0.4% A	2.7% A
	N16	3.0% A	2.7% A	0.3% A	0.5% A	1.0% ABC
	C21	2.7% A	0.9% A	0.2% A	0.7% A	0.7% C
	C23	1.2% A	1.6% A	0.6% A	1.0% A	0.9% BC
	C25	2.2% A	1.6% A	0.8% A	0.7% A	0.7% C
	C29	0.9% A	2.1% A	0.2% A	0.4% A	1.2% ABC
	N33	1.9% A	1.0% A	0.4% A	0.5% A	1.2% ABC
	p-value	0.009	0.063	0.600	0.753	< 0.001

Supplementary Table 8. Pairwise comparison between genotypes in each substrate type in the greenhouse experiment for bacterial and fungal taxa relative abundance. Two-way ANOVAs were used to discern how substrate type, genotype and their interaction influenced taxa relative abundance. Additional analyses were performed for each substrate type separately and between substrate types to better assess the indivudial effects of genotype and substrate type.

**Bacteria**		*Pirellulaceae_g*	*Planctomyces*	*Rhizobiales_f_g*	*Rhodoplanes*	*Rhodospirillaceae_g*
	Genotype	0.962	0.596	0.088	< 0.001	< 0.001
	Substrate type	< 0.001	< 0.001	0.006	< 0.001	< 0.001
	Interaction	0.068	0.001	0.186	< 0.001	< 0.001
**Pairwise comparison between substrate types**						
	Control	1.2% A	2.5% B	2.3% A	3.1% C	10.2% A
	Tailings	1.2% A	2.5% B	1.9% B	3.9% B	4.8% C
	Waste rock	0.5% B	3.7% A	1.8% B	5.7% A	6.7% B
	p-value	< 0.001	0.004	0.015	< 0.001	< 0.001
**Pairwise comparison by substrate type**						
Control	W08	0.8% A	2.2% A	2.2% A	2.8% A	7.8% D
	W09	1.1% A	2.4% A	2.6% A	2.5% A	9.8% BCD
	W10	1.1% A	2.4% A	2.0% A	3.7% A	11.3% ABC
	W13	0.8% A	2.0% A	1.9% A	2.7% A	9.1% CD
	N16	1.7% A	2.2% A	2.4% A	2.8% A	8.7% CD
	C21	1.7% A	3.1% A	3.1% A	3.7% A	14.8% A
	C23	1.4% A	2.6% A	2.2% A	3.3% A	10.3% BCD
	C25	1.1% A	2.2% A	1.7% A	3.3% A	9.9% BCD
	C29	1.2% A	2.9% A	2.0% A	2.9% A	12.3% AB
	N33	1.2% A	2.8% A	3.0% A	3.1% A	9.3% BCD
	p-value	0.201	0.697	0.238	0.098	< 0.001
Tailngs	W08	1.0% A	2.5% A	1.9% A	3.1% B	4.6% ABC
	W09	1.3% A	3.2% A	1.8% A	4.4% AB	4.5% BC
	W10	1.1% A	2.2% A	2.0% A	3.6% B	4.2% BC
	W13	1.9% A	2.5% A	2.4% A	4.0% AB	5.3% ABC
	N16	1.5% A	2.9% A	1.9% A	3.6% AB	6.5% AB
	C21	1.3% A	2.5% A	2.2% A	4.1% AB	5.0% ABC
	C23	1.0% A	2.5% A	1.5% A	3.0% B	3.7% C
	C25	1.5% A	2.9% A	2.2% A	5.7% A	6.6% A
	C29	0.7% A	2.5% A	1.7% A	3.3% B	3.9% C
	N33	1.1% A	1.8% A	1.7% A	4.2% AB	4.6% ABC
	p-value	0.076	0.437	0.294	< 0.001	< 0.001
Waste rock	W08	0.7% A	2.5% B	2.3% A	3.9% B	6.6% A
	W09	0.5% A	2.7% B	1.8% A	5.6% AB	6.9% A
	W10	0.5% A	5.9% AB	1.4% A	7.0% AB	7.1% A
	W13	0.6% A	2.4% B	2.1% A	5.2% AB	5.8% A
	N16	0.5% A	2.7% B	2.0% A	6.3% AB	6.3% A
	C21	0.5% A	7.3% A	2.6% A	5.4% AB	5.0% A
	C23	0.4% A	2.6% B	1.7% A	7.9% A	8.5% A
	C25	0.5% A	4.0% AB	1.4% A	6.5% AB	7.5% A
	C29	0.6% A	2.9% B	1.5% A	4.9% AB	7.6% A
	N33	0.4% A	4.3% AB	1.1% A	5.2% AB	6.3% A
	p-value	0.969	< 0.001	0.068	0.002	0.598

Supplementary Table 8. Pairwise comparison between genotypes in each substrate type in the greenhouse experiment for bacterial and fungal taxa relative abundance. Two-way ANOVAs were used to discern how substrate type, genotype and their interaction influenced taxa relative abundance. Additional analyses were performed for each substrate type separately and between substrate types to better assess the indivudial effects of genotype and substrate type.

**Bacteria**		*Rubrivivax*	*Sinobacteraceae_g*	*Solibacterales_f_g*	*Solirubrobacterales_f_g*
	Genotype	0.191	0.003	0.056	0.031
	Substrate type	< 0.001	< 0.001	< 0.001	< 0.001
	Interaction	0.129	< 0.001	0.278	0.043
**Pairwise comparison between substrate types**					
	Control	0.2% B	1.6% B	1.4% B	1.4% A
	Tailings	3.8% A	0.9% C	2.1% A	0.5% C
	Waste rock	0.2% B	2.8% A	1.3% B	0.8% B
	p-value	< 0.001	< 0.001	< 0.001	< 0.001
**Pairwise comparison by substrate type**					
Control	W08	0.0% A	1.1% A	1.9% A	1.7% A
	W09	0.0% A	1.9% A	1.2% A	1.7% A
	W10	0.1% A	1.4% A	1.1% A	1.2% A
	W13	0.4% A	2.1% A	1.4% A	1.4% A
	N16	0.1% A	1.9% A	1.4% A	1.2% A
	C21	0.0% A	1.6% A	1.5% A	1.1% A
	C23	0.3% A	1.2% A	1.8% A	1.6% A
	C25	0.1% A	1.6% A	1.2% A	1.0% A
	C29	0.5% A	1.6% A	1.6% A	1.6% A
	N33	0.1% A	1.6% A	1.2% A	1.2% A
	p-value	0.717	0.142	0.361	0.014
Tailngs	W08	3.2% A	0.8% A	1.8% A	0.5% AB
	W09	4.7% A	1.4% A	2.3% A	0.9% A
	W10	2.1% A	0.7% A	2.0% A	0.5% AB
	W13	2.1% A	1.0% A	2.3% A	0.3% B
	N16	2.2% A	1.0% A	1.6% A	0.6% AB
	C21	3.1% A	0.8% A	1.9% A	0.4% B
	C23	6.7% A	0.7% A	2.3% A	0.6% AB
	C25	2.8% A	1.3% A	1.6% A	0.6% AB
	C29	7.8% A	0.6% A	2.7% A	0.5% AB
	N33	2.4% A	0.9% A	2.5% A	0.4% AB
	p-value	0.009	0.009	0.026	0.011
Waste rock	W08	0.0% A	3.4% AB	1.2% A	0.8% A
	W09	0.1% A	2.3% B	1.2% A	0.7% A
	W10	0.1% A	2.8% AB	1.7% A	0.7% A
	W13	1.0% A	2.5% B	1.4% A	0.8% A
	N16	0.0% A	3.2% AB	1.2% A	0.8% A
	C21	0.0% A	4.7% A	0.8% A	0.7% A
	C23	0.2% A	1.7% B	1.9% A	0.6% A
	C25	0.3% A	2.5% B	1.4% A	0.8% A
	C29	0.2% A	2.3% B	1.5% A	0.8% A
	N33	0.0% A	3.1% AB	1.3% A	1.2% A
	p-value	0.423	< 0.001	0.451	0.760

Supplementary Table 8. Pairwise comparison between genotypes in each substrate type in the greenhouse experiment for bacterial and fungal taxa relative abundance. Two-way ANOVAs were used to discern how substrate type, genotype and their interaction influenced taxa relative abundance. Additional analyses were performed for each substrate type separately and between substrate types to better assess the indivudial effects of genotype and substrate type.

**Bacteria**		*Sphingobacteriaceae_g*	*Phycisphaerae WD2101_f_g*	*Xanthomonadaceae_g*
	Genotype	0.017	0.007	0.837
	Substrate type	< 0.001	< 0.001	< 0.001
	Interaction	0.674	0.114	0.063
**Pairwise comparison between substrate types**				
	Control	0.9% B	3.2% A	0.1% B
	Tailings	0.2% C	0.9% C	0.0% C
	Waste rock	2.1% A	2.0% B	6.4% A
	p-value	< 0.001	< 0.001	< 0.001
**Pairwise comparison by substrate type**				
Control	W08	0.9% A	4.0% A	0.1% A
	W09	0.7% A	4.9% A	0.1% A
	W10	1.1% A	2.9% A	0.1% A
	W13	0.7% A	2.8% A	0.1% A
	N16	0.6% A	2.2% A	0.0% A
	C21	1.4% A	4.1% A	0.1% A
	C23	0.5% A	2.8% A	0.0% A
	C25	1.2% A	2.5% A	0.1% A
	C29	0.6% A	2.7% A	0.1% A
	N33	1.3% A	2.7% A	0.1% A
	p-value	0.749	0.040	0.873
Tailngs	W08	0.1% A	1.1% AB	0.0% A
	W09	0.1% A	0.9% AB	0.0% A
	W10	0.2% A	0.6% AB	0.0% A
	W13	0.4% A	1.1% AB	0.0% A
	N16	0.2% A	0.8% AB	0.0% A
	C21	0.2% A	1.3% A	0.0% A
	C23	0.2% A	0.6% B	0.1% A
	C25	0.5% A	1.4% A	0.0% A
	C29	0.1% A	0.8% AB	0.0% A
	N33	0.3% A	0.9% AB	0.0% A
	p-value	0.057	0.002	0.300
Waste rock	W08	1.8% A	2.9% A	5.4% A
	W09	1.4% A	2.6% A	5.4% A
	W10	2.5% A	1.7% A	6.6% A
	W13	1.8% A	2.5% A	7.5% A
	N16	2.3% A	1.5% A	6.1% A
	C21	3.3% A	1.0% A	9.5% A
	C23	1.7% A	2.5% A	4.2% A
	C25	2.2% A	1.4% A	8.4% A
	C29	1.4% A	2.0% A	3.4% A
	N33	2.7% A	1.3% A	8.1% A
	p-value	0.182	0.474	0.039

Supplementary Table 8. Pairwise comparison between genotypes in each substrate type in the greenhouse experiment for bacterial and fungal taxa relative abundance. Two-way ANOVAs were used to discern how substrate type, genotype and their interaction influenced taxa relative abundance. Additional analyses were performed for each substrate type separately and between substrate types to better assess the indivudial effects of genotype and substrate type.

**Fungi**		*Acidea*	*Alternaria*	*Articulospora*	*Cadophora*	*Cephalothecaceae_g*	*Chrysosporium*
	Genotype	0.293	0.306	0.634	0.005	0.753	0.904
	Substrate type	< 0.001	< 0.001	< 0.001	0.081	0.089	< 0.001
	Interaction	0.626	0.790	0.809	0.329	0.107	0.133
**Pairwise comparison between substrate types**							
	Control	0.0% B	2.1% A	0.8% A	0.2% A	1.7% A	18.1% A
	Tailings	0.0% B	0.7% B	0.2% B	0.5% A	1.2% A	14.3% B
	Waste rock	3.3% A	0.7% B	0.3% B	0.1% A	1.5% A	11.6% B
	p-value	< 0.001	< 0.001	< 0.001	0.101	0.079	< 0.001
**Pairwise comparison by substrate type**							
Control	W08	0.0% A	1.6% A	0.6% A	0.2% A	1.5% A	14.0% A
	W09	0.0% A	2.5% A	1.1% A	0.1% A	1.2% A	17.8% A
	W10	0.0% A	2.2% A	0.8% A	0.1% A	1.9% A	18.9% A
	W13	0.0% A	2.6% A	1.0% A	0.1% A	1.5% A	19.9% A
	N16	0.0% A	2.1% A	0.9% A	0.0% A	1.4% A	18.7% A
	C21	0.0% A	1.1% A	0.7% A	0.1% A	1.2% A	20.9% A
	C23	0.0% A	2.2% A	0.8% A	0.8% A	2.1% A	16.6% A
	C25	0.0% A	2.5% A	0.9% A	0.1% A	2.2% A	21.0% A
	C29	0.0% A	1.6% A	0.5% A	0.1% A	1.2% A	15.2% A
	N33	0.0% A	2.7% A	1.0% A	0.2% A	2.3% A	19.0% A
	p-value	0.630	0.344	0.557	0.144	0.761	0.496
Tailngs	W08	0.0% A	1.1% A	0.3% A	0.3% A	2.0% A	17.7% A
	W09	0.0% A	0.3% A	0.1% A	2.1% A	0.9% A	10.3% A
	W10	0.0% A	0.9% A	0.3% A	0.4% A	1.7% A	16.4% A
	W13	0.0% A	1.2% A	0.4% A	0.2% A	0.7% A	11.1% A
	N16	0.0% A	0.9% A	0.3% A	0.1% A	1.7% A	12.6% A
	C21	0.0% A	0.3% A	0.2% A	0.3% A	1.4% A	20.7% A
	C23	0.0% A	0.9% A	0.3% A	0.7% A	0.7% A	12.0% A
	C25	0.0% A	0.6% A	0.3% A	0.3% A	1.5% A	11.6% A
	C29	0.0% A	0.3% A	0.2% A	0.1% A	0.8% A	16.8% A
	N33	0.0% A	0.5% A	0.2% A	0.3% A	1.4% A	13.6% A
	p-value	0.261	0.893	0.968	0.091	0.045	0.107
Waste rock	W08	3.4% A	1.0% A	0.5% A	0.2% A	1.3% A	9.0% A
	W09	3.7% A	0.9% A	0.4% A	0.1% A	2.1% A	14.8% A
	W10	2.6% A	0.6% A	0.1% A	0.0% A	0.8% A	9.9% A
	W13	2.0% A	0.8% A	0.3% A	0.4% A	1.9% A	15.5% A
	N16	2.1% A	0.6% A	0.2% A	0.0% A	1.5% A	10.5% A
	C21	2.9% A	0.2% A	0.1% A	0.1% A	1.1% A	8.8% A
	C23	3.9% A	1.4% A	0.7% A	0.4% A	1.3% A	12.3% A
	C25	3.2% A	0.2% A	0.1% A	0.0% A	1.3% A	11.0% A
	C29	1.9% A	0.9% A	0.2% A	0.1% A	1.7% A	13.4% A
	N33	5.5% A	0.5% A	0.2% A	0.1% A	1.6% A	11.6% A
	p-value	0.540	0.413	0.431	0.079	0.480	0.743

Supplementary Table 8. Pairwise comparison between genotypes in each substrate type in the greenhouse experiment for bacterial and fungal taxa relative abundance. Two-way ANOVAs were used to discern how substrate type, genotype and their interaction influenced taxa relative abundance. Additional analyses were performed for each substrate type separately and between substrate types to better assess the indivudial effects of genotype and substrate type.

**Fungi**		*Ciliophora*	*Cladosporium*	*Eurotiomycetes_o_f_g*	*Fusarium*	*Gibberella*
	Genotype	< 0.001	0.385	0.008	0.282	0.145
	Substrate type	< 0.001	< 0.001	< 0.001	0.681	< 0.001
	Interaction	0.136	0.917	0.008	0.842	0.732
**Pairwise comparison between substrate types**						
	Control	15.0% A	0.8% A	0.4% B	2.1% A	0.9% A
	Tailings	4.0% B	0.3% B	0.7% A	2.1% A	0.4% B
	Waste rock	2.7% B	0.3% B	0.8% A	2.6% A	0.3% B
	p-value	< 0.001	< 0.001	< 0.001	0.676	< 0.001
**Pairwise comparison by substrate type**						
Control	W08	26.9% A	0.7% A	0.8% A	1.8% A	0.6% A
	W09	17.1% AB	0.7% A	0.4% A	2.4% A	1.1% A
	W10	16.8% AB	0.9% A	0.1% A	2.0% A	1.1% A
	W13	14.5% AB	1.1% A	0.7% A	2.4% A	1.2% A
	N16	1.7% B	0.9% A	0.3% A	2.1% A	0.9% A
	C21	13.6% AB	0.2% A	0.2% A	1.2% A	0.4% A
	C23	21.6% AB	0.9% A	0.6% A	2.1% A	1.0% A
	C25	14.8% AB	0.8% A	0.3% A	2.2% A	1.2% A
	C29	7.4% AB	0.5% A	0.2% A	1.5% A	0.7% A
	N33	10.5% AB	0.8% A	0.3% A	2.6% A	1.0% A
	p-value	0.015	0.521	0.044	0.343	0.486
Tailngs	W08	7.3% AB	0.3% A	1.1% A	0.9% A	0.3% A
	W09	3.6% AB	0.6% A	0.7% A	4.9% A	0.2% A
	W10	2.9% AB	0.3% A	1.0% A	3.3% A	0.4% A
	W13	2.1% AB	0.6% A	0.9% A	1.8% A	0.6% A
	N16	2.3% AB	0.3% A	0.4% A	2.7% A	0.7% A
	C21	1.3% B	0.2% A	0.7% A	2.0% A	0.1% A
	C23	6.5% AB	0.3% A	0.9% A	1.5% A	0.5% A
	C25	0.5% B	0.4% A	0.3% A	1.6% A	0.5% A
	C29	0.8% B	0.1% A	0.7% A	1.4% A	0.3% A
	N33	11.2% A	0.2% A	0.5% A	1.1% A	0.2% A
	p-value	0.001	0.762	0.110	0.656	0.720
Waste rock	W08	5.5% A	0.4% A	0.5% A	2.1% A	0.4% A
	W09	4.6% A	0.4% A	1.2% A	3.7% A	0.5% A
	W10	0.4% A	0.2% A	0.4% A	0.8% A	0.3% A
	W13	1.7% A	0.3% A	1.3% A	4.0% A	0.4% A
	N16	1.0% A	0.3% A	1.1% A	0.7% A	0.2% A
	C21	1.3% A	0.1% A	0.3% A	0.6% A	0.1% A
	C23	1.9% A	0.4% A	1.0% A	2.5% A	0.7% A
	C25	0.9% A	0.2% A	1.3% A	2.5% A	0.2% A
	C29	2.7% A	0.4% A	1.0% A	6.1% A	0.3% A
	N33	4.0% A	0.2% A	0.6% A	2.5% A	0.3% A
	p-value	0.435	0.730	0.002	0.729	0.176

Supplementary Table 8. Pairwise comparison between genotypes in each substrate type in the greenhouse experiment for bacterial and fungal taxa relative abundance. Two-way ANOVAs were used to discern how substrate type, genotype and their interaction influenced taxa relative abundance. Additional analyses were performed for each substrate type separately and between substrate types to better assess the indivudial effects of genotype and substrate type.

**Fungi**		*Lecythophora*	*Leptosphaeria*	*Lindtneria*	*Meliniomyces*	*Mortierella*	*Pezoloma*
	Genotype	0.039	0.753	0.901	0.196	0.386	0.209
	Substrate type	0.253	0.004	0.994	< 0.001	< 0.001	< 0.001
	Interaction	0.519	0.203	0.527	0.046	0.478	0.029
**Pairwise comparison between substrate types**							
	Control	0.3% A	0.0% B	0.7% A	0.0% B	0.6% A	0.0% B
	Tailings	0.3% A	0.9% A	0.6% A	0.1% B	0.2% B	0.0% B
	Waste rock	0.5% A	0.0% B	0.6% A	2.3% A	0.2% B	6.6% A
	p-value	0.194	< 0.001	0.986	0.006	< 0.001	< 0.001
**Pairwise comparison by substrate type**							
Control	W08	0.5% A	0.0% A	0.8% A	0.1% A	0.3% A	0.0% A
	W09	0.3% A	0.0% A	0.0% A	0.0% A	0.7% A	0.0% A
	W10	0.1% A	0.0% A	0.6% A	0.0% A	0.7% A	0.0% A
	W13	0.0% A	0.0% A	0.2% A	0.0% A	0.6% A	0.0% A
	N16	0.0% A	0.0% A	0.2% A	0.1% A	0.7% A	0.0% A
	C21	0.5% A	0.0% A	0.0% A	0.0% A	0.3% A	0.1% A
	C23	0.4% A	0.0% A	0.6% A	0.1% A	0.6% A	0.0% A
	C25	0.3% A	0.0% A	0.6% A	0.0% A	0.7% A	0.0% A
	C29	0.0% A	0.0% A	1.0% A	0.0% A	0.4% A	0.0% A
	N33	0.8% A	0.0% A	2.4% A	0.0% A	0.7% A	0.0% A
	p-value	0.034	0.508	0.770	0.202	0.153	0.169
Tailngs	W08	0.7% A	0.4% A	0.7% A	0.1% A	0.2% A	0.0% A
	W09	0.1% A	1.1% A	0.1% A	0.0% A	0.1% A	0.0% A
	W10	0.1% A	2.1% A	1.5% A	0.6% A	0.2% A	0.0% A
	W13	0.2% A	0.1% A	1.9% A	0.0% A	0.2% A	0.0% A
	N16	0.0% A	5.2% A	0.0% A	0.0% A	0.2% A	0.0% A
	C21	0.3% A	0.3% A	1.5% A	0.0% A	0.1% A	0.0% A
	C23	0.2% A	0.8% A	0.0% A	0.0% A	0.3% A	0.0% A
	C25	0.1% A	0.1% A	0.0% A	0.0% A	0.2% A	0.0% A
	C29	0.2% A	0.1% A	0.0% A	0.0% A	0.1% A	0.0% A
	N33	0.7% A	0.1% A	0.6% A	0.0% A	0.1% A	0.1% A
	p-value	0.690	0.116	0.572	0.486	0.947	0.030
Waste rock	W08	0.4% A	0.0% A	0.4% A	2.3% AB	0.2% A	5.7% A
	W09	0.3% A	0.0% A	0.9% A	0.3% B	0.4% A	5.4% A
	W10	0.1% A	0.0% A	0.2% A	2.9% AB	0.2% A	9.6% A
	W13	0.9% A	0.0% A	1.4% A	0.0% B	0.2% A	1.4% A
	N16	0.2% A	0.0% A	1.7% A	2.1% AB	0.2% A	8.4% A
	C21	0.2% A	0.0% A	0.4% A	1.4% AB	0.0% A	9.3% A
	C23	0.8% A	0.0% A	0.5% A	2.4% AB	0.3% A	7.9% A
	C25	1.1% A	0.0% A	0.8% A	0.1% B	0.1% A	8.5% A
	C29	0.7% A	0.0% A	0.4% A	0.2% B	0.4% A	3.1% A
	N33	0.4% A	0.0% A	0.4% A	9.0% A	0.1% A	6.5% A
	p-value	0.340	0.730	0.554	0.002	0.124	0.261

Supplementary Table 8. Pairwise comparison between genotypes in each substrate type in the greenhouse experiment for bacterial and fungal taxa relative abundance. Two-way ANOVAs were used to discern how substrate type, genotype and their interaction influenced taxa relative abundance. Additional analyses were performed for each substrate type separately and between substrate types to better assess the indivudial effects of genotype and substrate type.

**Fungi**		*Phaeosphaeriaceae_g*	*Pleosporale_f_g*	*Pleosporales_ fam_Incertae_sedis_g*	*Pyrenopeziza*
	Genotype	0.422	0.411	0.265	0.384
	Substrate type	< 0.001	< 0.001	< 0.001	< 0.001
	Interaction	0.755	0.522	0.933	0.622
**Pairwise comparison between substrate types**					
	Control	0.7% A	0.5% A	3.1% A	0.0% B
	Tailings	0.2% B	0.2% B	0.9% B	0.0% B
	Waste rock	0.2% B	0.2% B	1.0% B	0.9% A
	p-value	< 0.001	< 0.001	< 0.001	< 0.001
**Pairwise comparison by substrate type**					
Control	W08	0.6% A	0.4% A	2.3% A	0.0% A
	W09	1.1% A	0.5% A	3.6% A	0.0% A
	W10	0.6% A	0.5% A	3.5% A	0.0% A
	W13	0.9% A	0.6% A	3.6% A	0.0% A
	N16	0.6% A	0.6% A	3.1% A	0.0% A
	C21	0.4% A	0.3% A	1.5% A	0.0% A
	C23	0.7% A	0.6% A	3.4% A	0.0% A
	C25	0.9% A	0.4% A	3.3% A	0.0% A
	C29	0.5% A	0.4% A	2.6% A	0.0% A
	N33	0.8% A	0.5% A	3.5% A	0.0% A
	p-value	0.190	0.511	0.425	0.987
Tailngs	W08	0.3% A	0.4% A	1.2% A	0.0% A
	W09	0.1% A	0.0% A	0.4% A	0.0% A
	W10	0.3% A	0.4% A	1.0% A	0.0% A
	W13	0.3% A	0.2% A	1.5% A	0.0% A
	N16	0.2% A	0.2% A	0.8% A	0.0% A
	C21	0.1% A	0.2% A	0.6% A	0.0% A
	C23	0.2% A	0.2% A	1.2% A	0.0% A
	C25	0.2% A	0.1% A	1.2% A	0.0% A
	C29	0.1% A	0.1% A	0.4% A	0.0% A
	N33	0.2% A	0.1% A	0.7% A	0.0% A
	p-value	0.892	0.282	0.952	0.437
Waste rock	W08	0.3% A	0.2% A	1.2% A	0.6% A
	W09	0.3% A	0.2% A	1.7% A	1.2% A
	W10	0.1% A	0.1% A	1.0% A	0.7% A
	W13	0.2% A	0.3% A	1.0% A	0.8% A
	N16	0.2% A	0.2% A	0.5% A	0.7% A
	C21	0.1% A	0.1% A	0.3% A	1.0% A
	C23	0.3% A	0.3% A	1.6% A	1.0% A
	C25	0.2% A	0.1% A	0.6% A	0.6% A
	C29	0.3% A	0.3% A	1.0% A	0.9% A
	N33	0.2% A	0.1% A	0.8% A	1.5% A
	p-value	0.580	0.681	0.300	0.334

Supplementary Table 8. Pairwise comparison between genotypes in each substrate type in the greenhouse experiment for bacterial and fungal taxa relative abundance. Two-way ANOVAs were used to discern how substrate type, genotype and their interaction influenced taxa relative abundance. Additional analyses were performed for each substrate type separately and between substrate types to better assess the indivudial effects of genotype and substrate type.

**Fungi**		*Pyrenophora*	*Russula*	*Sebacinales_f_g*	*Sordariales_f_g*	*Sphaerosporella*	*Tomentella*
	Genotype	0.514	0.892	0.008	0.639	0.031	0.395
	Substrate type	< 0.001	< 0.001	< 0.001	< 0.001	< 0.001	< 0.001
	Interaction	0.649	0.005	0.039	0.252	0.671	0.527
**Pairwise comparison between substrate types**							
	Control	0.9% A	0.0% B	0.3% B	0.1% B	9.5% A	31.6% A
	Tailings	0.2% B	0.1% B	1.7% A	33.0% A	7.7% A	20.1% B
	Waste rock	0.3% B	6.0% A	0.4% B	0.5% B	3.0% B	40.5% A
	p-value	< 0.001	< 0.001	< 0.001	< 0.001	< 0.001	< 0.001
**Pairwise comparison by substrate type**							
Control	W08	0.6% A	0.0% A	0.3% A	0.0% A	13.6% A	23.0% A
	W09	0.7% A	0.0% A	0.4% A	0.1% A	6.5% A	31.2% A
	W10	0.9% A	0.0% A	0.1% A	0.1% A	3.8% A	33.0% A
	W13	1.2% A	0.0% A	0.2% A	0.0% A	3.7% A	33.1% A
	N16	1.0% A	0.0% A	0.3% A	0.1% A	4.0% A	51.8% A
	C21	0.3% A	0.0% A	0.7% A	0.0% A	19.3% A	30.3% A
	C23	0.7% A	0.0% A	0.3% A	0.1% A	12.0% A	21.8% A
	C25	1.2% A	0.0% A	0.2% A	0.1% A	11.2% A	24.0% A
	C29	0.6% A	0.0% A	0.1% A	0.0% A	14.5% A	44.1% A
	N33	1.1% A	0.0% A	0.4% A	0.1% A	8.2% A	30.2% A
	p-value	0.279	0.910	0.048	0.634	0.246	0.211
Tailngs	W08	0.2% A	0.0% A	1.7% AB	28.1% A	3.0% A	21.0% A
	W09	0.1% A	0.0% A	2.2% AB	27.7% A	6.2% A	28.4% A
	W10	0.3% A	1.1% A	1.9% AB	46.6% A	1.5% A	5.8% A
	W13	0.5% A	0.0% A	1.2% B	27.7% A	10.2% A	28.6% A
	N16	0.1% A	0.0% A	0.4% B	34.7% A	7.5% A	19.2% A
	C21	0.2% A	0.0% A	5.7% A	31.2% A	9.9% A	9.0% A
	C23	0.3% A	0.0% A	1.3% B	27.3% A	14.7% A	17.4% A
	C25	0.2% A	0.0% A	1.3% B	44.2% A	11.1% A	17.5% A
	C29	0.2% A	0.0% A	0.2% B	30.9% A	8.6% A	31.2% A
	N33	0.1% A	0.0% A	1.0% B	33.8% A	2.3% A	23.3% A
	p-value	0.950	0.410	< 0.001	0.211	0.753	0.695
Waste rock	W08	0.4% A	7.6% AB	1.5% A	0.0% A	2.1% A	43.5% A
	W09	0.4% A	1.4% B	0.4% A	0.0% A	6.1% A	37.5% A
	W10	0.2% A	16.2% A	0.2% A	0.0% A	1.2% A	45.9% A
	W13	0.3% A	0.0% AB	0.4% A	0.0% A	2.5% A	50.1% A
	N16	0.2% A	3.1% AB	0.2% A	0.1% A	1.0% A	53.4% A
	C21	0.0% A	5.3% AB	0.2% A	5.1% A	0.6% A	51.7% A
	C23	0.5% A	2.3% AB	0.4% A	0.0% A	5.6% A	32.5% A
	C25	0.2% A	14.1% AB	0.2% A	0.0% A	1.0% A	40.1% A
	C29	0.3% A	0.6% AB	0.2% A	0.0% A	8.4% A	26.2% A
	N33	0.2% A	6.7% AB	0.4% A	0.2% A	0.9% A	34.1% A
	p-value	0.152	0.001	0.839	0.390	0.069	0.542

Supplementary Table 8. Pairwise comparison between genotypes in each substrate type in the greenhouse experiment for bacterial and fungal taxa relative abundance. Two-way ANOVAs were used to discern how substrate type, genotype and their interaction influenced taxa relative abundance. Additional analyses were performed for each substrate type separately and between substrate types to better assess the indivudial effects of genotype and substrate type.

**Fungi**		*Trichoderma*		*Vibrisseaceae_g*	
	Genotype	0.815		0.002	
	Substrate type	0.012		< 0.001	
	Interaction	0.599		0.227	
**Pairwise comparison between substrate types**					
	Control	0.4%	B	0.2%	B
	Tailings	0.7%	AB	0.0%	C
	Waste rock	1.8%	A	0.6%	A
	p-value	0.009		< 0.001	
**Pairwise comparison by substrate type**					
Control	W08	0.9%	A	0.1%	A
	W09	0.1%	A	0.4%	A
	W10	0.2%	A	0.0%	A
	W13	0.1%	A	0.0%	A
	N16	0.1%	A	0.2%	A
	C21	0.1%	A	0.5%	A
	C23	0.9%	A	0.3%	A
	C25	0.5%	A	0.1%	A
	C29	0.1%	A	0.1%	A
	N33	0.6%	A	0.4%	A
	p-value	0.754		0.052	
Tailngs	W08	0.5%	A	0.0%	B
	W09	1.0%	A	0.0%	B
	W10	0.8%	A	0.0%	B
	W13	0.4%	A	0.0%	B
	N16	1.5%	A	0.0%	AB
	C21	0.4%	A	0.1%	A
	C23	1.0%	A	0.0%	B
	C25	0.1%	A	0.0%	AB
	C29	0.9%	A	0.0%	B
	N33	0.3%	A	0.0%	AB
	p-value	0.601		0.005	
Waste rock	W08	0.3%	A	1.3%	A
	W09	1.5%	A	0.4%	A
	W10	0.3%	A	0.4%	A
	W13	4.5%	A	0.2%	A
	N16	2.7%	A	0.2%	A
	C21	1.0%	A	0.7%	A
	C23	2.7%	A	1.3%	A
	C25	1.4%	A	0.3%	A
	C29	0.8%	A	0.1%	A
	N33	4.0%	A	0.4%	A
	p-value	0.542		0.541	
