# Supplementary material for: Plant Genotype Influences Physicochemical Properties of Substrate as Well as Bacterial and Fungal Assemblages in the Rhizosphere of Balsam Poplar
Source: Front Microbiol. 2020 Nov 23;11:575625. doi: 10.3389/fmicb.2020.575625 (PMC7719689; doi:10.3389/fmicb.2020.575625)
Supplement: Supplementary file 17 [file Table_11.PDF]

Supplementary Table 11. Pairwise comparison between origin of cuttings in each substrate type in the greenhouse experiment for bacterial and fungal taxa relative abundance. Two-way ANOVAs were used to discern how substrate type (Control, Tailings or Waste rock), origin of cuttings (La Corne Mine, Westwood or Natural forest) and their interaction influenced taxa relative abundance. Additional analyses were performed for each substrate type separately and between substrate types to better assess the indivudial effects of origin and substrate type.

**Bacteria**		*Anaerolineae SBR1031 A4b_g*	*Acidobacteriaceae_g*	*Bradyrhizobium*	*Caulobacteraceae_g*	*Chitinophagaceae_g*	*Acidimicrobiales EB1017_g*
	Origin	0.511	0.300	0.120	0.062	0.030	0.252
	Substrate type	< 0.001	< 0.001	< 0.001	< 0.001	< 0.001	< 0.001
	Interaction	0.562	0.732	0.259	0.461	0.380	0.003
**Pairwise comparison between origin**							
	Natural	0.9% B	0.8% A	0.9% A	1.1% A	13.0% A	0.8% A
	La Corne Mine	1.0% A	0.7% A	0.9% A	1.0% A	12.8% A	0.7% A
	Westwood	0.9% B	0.7% A	0.9% A	0.9% A	15.1% A	0.8% A
	p-value	0.688	0.873	0.995	0.742	0.323	0.574
**Pairwise comparison by substrate type**							
Control	Natural	0.9% A	0.1% A	0.4% AB	0.5% A	23.7% AB	1.0% A
	La Corne Mine	0.8% A	0.1% A	0.5% A	0.6% A	19.8% B	0.9% A
	Westwood	0.7% A	0.1% A	0.4% B	0.5% A	24.1% A	0.7% A
	p-value	0.331	0.576	0.030	0.179	0.031	0.064
Tailings	Natural	1.4% A	0.1% A	0.8% A	0.4% A	11.2% A	1.2% AB
	La Corne Mine	1.3% A	0.0% A	1.0% A	0.6% A	11.3% A	0.9% B
	Westwood	1.4% A	0.0% A	0.9% A	0.5% A	12.7% A	1.3% A
	p-value	0.750	0.199	0.743	0.112	0.465	0.014
Waste rock	Natural	0.6% A	2.0% A	1.3% A	2.0% A	6.6% A	0.3% A
	La Corne Mine	0.8% A	1.9% A	1.1% A	1.7% A	7.8% A	0.3% A
	Westwood	0.6% A	1.8% A	1.3% A	1.7% A	9.3% A	0.4% A
	p-value	0.465	0.954	0.377	0.509	0.250	0.111

Supplementary Table 11. Pairwise comparison between origin of cuttings in each substrate type in the greenhouse experiment for bacterial and fungal taxa relative abundance. Two-way ANOVAs were used to discern how substrate type (Control, Tailings or Waste rock), origin of cuttings (La Corne Mine, Westwood or Natural forest) and their interaction influenced taxa relative abundance. Additional analyses were performed for each substrate type separately and between substrate types to better assess the indivudial effects of origin and substrate type.

**Bacteria**		*Alphaproteobacteria Ellin329_f_g*	*Betaproteobacteria Ellin6067_f_g*	*Frankiaceae_g*	*Gaiellaceae_g*	*Gemmataceae_g*	*Geobacter*	*Acidobacteria-6 iii1-15_f_g*
	Origin	0.812	0.604	0.129	0.134	0.094	0.025	0.391
	Substrate type	< 0.001	< 0.001	< 0.001	< 0.001	< 0.001	< 0.001	< 0.001
	Interaction	0.584	0.377	0.153	0.055	0.433	0.567	0.306
**Pairwise comparison between origin**								
	Natural	6.5% A	2.0% A	1.2% A	0.9% A	2.1% A	0.9% A	1.3% A
	La Corne Mine	5.9% A	1.9% A	0.9% A	0.9% A	1.8% A	2.9% A	1.2% A
	Westwood	6.3% A	1.9% A	1.1% A	0.9% A	2.1% A	1.6% A	1.3% A
	p-value	0.744	0.846	0.149	0.946	0.686	0.154	0.852
**Pairwise comparison by substrate type**								
Control	Natural	6.6% A	1.8% A	1.4% A	0.8% A	0.9% A	0.1% A	0.9% A
	La Corne Mine	6.3% A	2.1% A	1.4% A	0.9% A	0.7% A	0.4% A	0.9% A
	Westwood	5.8% A	2.1% A	1.4% A	0.8% A	0.9% A	0.0% A	1.0% A
	p-value	0.655	0.453	0.977	0.653	0.472	0.248	0.436
Tailings	Natural	3.9% A	1.9% A	1.1% A	0.5% AB	4.1% A	2.8% A	2.5% A
	La Corne Mine	3.6% A	1.6% A	0.6% B	0.5% B	3.2% A	7.3% A	1.9% A
	Westwood	3.4% A	1.5% A	0.7% AB	0.7% A	4.0% A	4.9% A	2.4% A
	p-value	0.722	0.185	0.027	0.003	0.033	0.191	0.127
Waste rock	Natural	8.3% A	2.1% A	1.1% A	1.2% A	1.6% A	0.1% A	0.7% A
	La Corne Mine	8.2% A	2.1% A	0.8% A	1.2% A	1.4% A	0.4% A	0.7% A
	Westwood	9.4% A	2.0% A	1.1% A	1.1% A	1.5% A	0.2% A	0.6% A
	p-value	0.415	0.875	0.175	0.640	0.898	0.123	0.634

Supplementary Table 11. Pairwise comparison between origin of cuttings in each substrate type in the greenhouse experiment for bacterial and fungal taxa relative abundance. Two-way ANOVAs were used to discern how substrate type (Control, Tailings or Waste rock), origin of cuttings (La Corne Mine, Westwood or Natural forest) and their interaction influenced taxa relative abundance. Additional analyses were performed for each substrate type separately and between substrate types to better assess the indivudial effects of origin and substrate type.

**Bacteria**		*Isosphaeraceae_g*	*Myxococcales_f_g*	*Opitutaceae_g*	*Opitutus*	*Pedosphaerales_f_g*	*Pirellulaceae_g*	*Planctomyces*
	Origin	0.624	0.024	0.750	0.085	0.014	1.000	0.357
	Substrate type	< 0.001	< 0.001	< 0.001	< 0.001	< 0.001	< 0.001	0.005
	Interaction	0.414	0.147	0.188	0.020	< 0.001	0.047	0.545
**Pairwise comparison between origin**								
	Natural	1.2% A	2.5% A	1.3% A	1.0% A	0.9% A	1.0% A	2.9% A
	La Corne Mine	0.8% A	2.1% A	1.0% A	0.8% A	0.9% A	1.0% A	3.2% A
	Westwood	0.8% A	2.5% A	1.3% A	0.8% A	1.0% A	0.9% A	2.7% A
	p-value	0.325	0.365	0.656	0.138	0.573	0.951	0.451
**Pairwise comparison by substrate type**								
Control	Natural	0.4% A	3.1% A	0.3% A	0.9% A	0.7% AB	1.4% A	2.6% A
	La Corne Mine	0.4% A	2.9% A	0.4% A	0.8% A	1.0% A	1.3% A	2.7% A
	Westwood	0.4% A	3.7% A	0.4% A	0.7% A	0.5% B	0.9% A	2.2% A
	p-value	0.790	0.293	0.683	0.242	< 0.001	0.030	0.290
Tailings	Natural	0.3% A	3.2% A	3.7% A	1.8% A	0.8% A	1.2% A	2.2% A
	La Corne Mine	0.3% A	2.0% B	1.9% A	1.0% B	0.8% A	1.1% A	2.6% A
	Westwood	0.4% A	2.5% AB	3.3% A	1.1% B	0.6% A	1.3% A	2.6% A
	p-value	0.473	0.004	0.063	0.002	0.301	0.554	0.516
Waste rock	Natural	2.4% A	1.7% A	0.3% A	0.5% A	1.1% AB	0.5% A	3.6% A
	La Corne Mine	1.7% A	1.5% A	0.4% A	0.7% A	0.9% B	0.5% A	4.2% A
	Westwood	1.6% A	1.4% A	0.4% A	0.6% A	1.8% A	0.6% A	3.3% A
	p-value	0.232	0.752	0.805	0.478	0.003	0.479	0.513

Supplementary Table 11. Pairwise comparison between origin of cuttings in each substrate type in the greenhouse experiment for bacterial and fungal taxa relative abundance. Two-way ANOVAs were used to discern how substrate type (Control, Tailings or Waste rock), origin of cuttings (La Corne Mine, Westwood or Natural forest) and their interaction influenced taxa relative abundance. Additional analyses were performed for each substrate type separately and between substrate types to better assess the indivudial effects of origin and substrate type.

**Bacteria**		*Rhizobiales_f_g*	*Rhodoplanes*	*Rhodospirillaceae_g*	*Rubrivivax*	*Sinobacteraceae_g*	*Solibacterales_f_g*
	Origin	0.689	0.192	0.217	0.426	0.436	0.594
	Substrate type	0.014	< 0.001	< 0.001	< 0.001	< 0.001	< 0.001
	Interaction	0.268	0.926	0.038	0.140	0.956	0.959
**Pairwise comparison between origin**							
	Natural	1.9% A	4.4% A	6.8% A	0.7% A	2.1% A	1.5% A
	La Corne Mine	1.9% A	4.5% A	7.6% A	2.0% A	1.7% A	1.7% A
	Westwood	2.0% A	4.1% A	6.9% A	1.1% A	1.8% A	1.6% A
	p-value	0.818	0.547	0.462	0.104	0.501	0.541
**Pairwise comparison by substrate type**							
Control	Natural	2.8% A	3.0% A	9.1% B	0.1% A	1.7% A	1.3% A
	La Corne Mine	2.2% A	3.3% A	11.6% A	0.2% A	1.5% A	1.5% A
	Westwood	2.2% A	2.9% A	9.5% B	0.1% A	1.6% A	1.4% A
	p-value	0.217	0.361	0.005	0.586	0.626	0.498
Tailings	Natural	1.7% A	3.9% A	5.3% A	2.3% A	1.0% A	2.1% A
	La Corne Mine	1.8% A	4.0% A	4.7% A	5.2% A	0.9% A	2.2% A
	Westwood	2.0% A	3.8% A	4.7% A	3.0% A	1.0% A	2.1% A
	p-value	0.484	0.892	0.489	0.037	0.676	0.937
Waste rock	Natural	1.5% A	5.6% A	6.3% A	0.0% A	3.1% A	1.3% A
	La Corne Mine	1.8% A	6.2% A	7.2% A	0.2% A	2.8% A	1.4% A
	Westwood	1.9% A	5.3% A	6.6% A	0.3% A	2.7% A	1.4% A
	p-value	0.489	0.382	0.607	0.622	0.741	0.926

Supplementary Table 11. Pairwise comparison between origin of cuttings in each substrate type in the greenhouse experiment for bacterial and fungal taxa relative abundance. Two-way ANOVAs were used to discern how substrate type (Control, Tailings or Waste rock), origin of cuttings (La Corne Mine, Westwood or Natural forest) and their interaction influenced taxa relative abundance. Additional analyses were performed for each substrate type separately and between substrate types to better assess the indivudial effects of origin and substrate type.

**Bacteria**		*Solirubrobacterales_f_g*	*Sphingobacteriaceae_g*	*Phycisphaerae WD2101_f_g*	*Xanthomonadaceae_g*
	Origin	0.543	0.718	0.230	0.670
	Substrate type	< 0.001	< 0.001	< 0.001	< 0.001
	Interaction	0.185	0.677	0.139	0.498
**Pairwise comparison between origin**					
	Natural	0.9% A	1.4% A	1.5% A	3.0% A
	La Corne Mine	0.8% A	1.1% A	1.8% A	2.2% A
	Westwood	0.9% A	1.0% A	2.4% A	2.3% A
	p-value	0.628	0.409	0.066	0.705
**Pairwise comparison by substrate type**					
Control	Natural	1.2% A	1.0% A	2.5% A	0.1% A
	La Corne Mine	1.3% A	0.9% A	2.9% A	0.1% A
	Westwood	1.5% A	0.8% A	3.6% A	0.1% A
	p-value	0.176	0.854	0.103	0.339
Tailings	Natural	0.5% A	0.2% A	0.8% A	0.0% A
	La Corne Mine	0.5% A	0.2% A	1.0% A	0.1% A
	Westwood	0.6% A	0.2% A	0.9% A	0.0% A
	p-value	0.816	0.917	0.749	0.544
Waste rock	Natural	1.0% A	2.5% A	1.4% A	7.2% A
	La Corne Mine	0.8% A	2.2% A	1.7% A	6.4% A
	Westwood	0.8% A	1.8% A	2.5% A	6.1% A
	p-value	0.171	0.287	0.093	0.674

Supplementary Table 11. Pairwise comparison between origin of cuttings in each substrate type in the greenhouse experiment for bacterial and fungal taxa relative abundance. Two-way ANOVAs were used to discern how substrate type (Control, Tailings or Waste rock), origin of cuttings (La Corne Mine, Westwood or Natural forest) and their interaction influenced taxa relative abundance. Additional analyses were performed for each substrate type separately and between substrate types to better assess the indivudial effects of origin and substrate type.

**Fungi**		*Acidea*	*Alternaria*	*Articulospora*	*Cadophora*	*Cephalothecaceae_g*	*Chrysosporium*	*Ciliophora*	*Cladosporium*
	Origin	0.672	0.397	0.562	0.541	0.349	0.950	0.123	0.121
	Substrate type	< 0.001	< 0.001	< 0.001	0.108	0.094	< 0.001	< 0.001	< 0.001
	Interaction	0.418	0.774	0.827	0.587	0.874	0.922	0.040	0.770
**Pairwise comparison between origin**									
	Natural	1.6% A	1.2% A	0.4% A	0.1% A	1.7% A	14.3% A	5.7% A	0.4% A
	La Corne Mine	1.0% A	1.0% A	0.4% A	0.3% A	1.4% A	14.8% A	5.9% A	0.4% A
	Westwood	1.1% A	1.3% A	0.5% A	0.3% A	1.5% A	14.4% A	8.6% A	0.5% A
	p-value	0.547	0.488	0.629	0.459	0.377	0.940	0.283	0.176
**Pairwise comparison by substrate type**									
Control	Natural	0,0% A	2.4% A	0.9% A	0.1% A	1.9% A	18.9% A	7.0% B	0.8% A
	La Corne Mine	0.0% A	1.9% A	0.8% A	0.3% A	1.7% A	18.2% A	14.5% AB	0.6% A
	Westwood	0.0% A	2.2% A	0.9% A	0.1% A	1.5% A	17.7% A	18.8% A	0.8% A
	p-value	0.807	0.432	0.497	0.424	0.677	0.846	0.031	0.519
Tailings	Natural	0.0% A	0.7% A	0.2% A	0.2% A	1.5% A	13.2% A	7.6% A	0.3% A
	La Corne Mine	0.0% A	0.6% A	0.2% A	0.4% A	1.0% A	15.0% A	2.6% A	0.2% A
	Westwood	0.0% A	0.9% A	0.3% A	0.8% A	1.3% A	13.9% A	4.0% A	0.5% A
	p-value	0.321	0.608	0.912	0.409	0.364	0.768	0.070	0.257
Waste rock	Natural	4.4% A	0.5% A	0.2% A	0.1% A	1.6% A	11.3% A	3.0% A	0.2% A
	La Corne Mine	3.0% A	0.7% A	0.3% A	0.1% A	1.4% A	11.4% A	1.7% A	0.3% A
	Westwood	3.1% A	0.8% A	0.3% A	0.2% A	1.5% A	12.0% A	3.5% A	0.4% A
	p-value	0.367	0.618	0.586	0.759	0.768	0.927	0.387	0.495

Supplementary Table 11. Pairwise comparison between origin of cuttings in each substrate type in the greenhouse experiment for bacterial and fungal taxa relative abundance. Two-way ANOVAs were used to discern how substrate type (Control, Tailings or Waste rock), origin of cuttings (La Corne Mine, Westwood or Natural forest) and their interaction influenced taxa relative abundance. Additional analyses were performed for each substrate type separately and between substrate types to better assess the indivudial effects of origin and substrate type.

**Fungi**		*Eurotiomycetes_o_f_g*	*Fusarium*	*Gibberella*	*Lecythophora*	*Leptosphaeria*	*Lindtneria*	*Meliniomyces*	*Mortierella*
	Origin	0.032	0.194	0.533	0.453	0.564	0.527	0.761	0.692
	Substrate type	< 0.001	0.692	< 0.001	0.199	0.005	0.986	0.001	< 0.001
	Interaction	0.516	0.759	0.817	0.408	0.293	0.485	0.008	0.578
**Pairwise comparison between origin**									
	Natural	0.5% A	2.0% A	0.5% A	0.4% A	0.7% A	0.9% A	2.5% A	0.3% A
	La Corne Mine	0.6% A	2.1% A	0.5% A	0.4% A	0.1% A	0.5% A	0.4% B	0.3% A
	Westwood	0.8% A	2.5% A	0.6% A	0.3% A	0.3% A	0.7% A	0.6% B	0.3% A
	p-value	0.195	0.720	0.728	0.615	0.350	0.516	0.010	0.751
**Pairwise comparison by substrate type**									
Control	Natural	0.3% A	2.4% A	0.9% A	0.5% A	0.0% A	1.5% A	0.0% A	0.7% A
	La Corne Mine	0.4% A	1.8% A	0.9% A	0.3% A	0.0% A	0.6% A	0.0% A	0.5% A
	Westwood	0.5% A	2.2% A	1.0% A	0.3% A	0.0% A	0.4% A	0.0% A	0.6% A
	p-value	0.313	0.195	0.789	0.401	0.390	0.307	0.841	0.492
Tailings	Natural	0.5% B	1.8% A	0.4% A	0.4% A	2.1% A	0.4% A	0.0% A	0.1% A
	La Corne Mine	0.7% AB	1.6% A	0.4% A	0.2% A	0.4% A	0.3% A	0.0% A	0.2% A
	Westwood	0.9% A	2.7% A	0.3% A	0.3% A	0.9% A	1.0% A	0.2% A	0.2% A
	p-value	0.022	0.469	0.943	0.529	0.240	0.395	0.441	0.924
Waste rock	Natural	0.8% A	1.9% A	0.2% A	0.4% A	0,0% A	0.8% A	6.7% A	0.1% A
	La Corne Mine	0.9% A	3.0% A	0.3% A	0.7% A	0,0% A	0.5% A	1.0% B	0.2% A
	Westwood	0.8% A	2.6% A	0.4% A	0.4% A	0.0% A	0.7% A	1.5% B	0.3% A
	p-value	0.903	0.832	0.330	0.292	0.508	0.775	0.002	0.360

Supplementary Table 11. Pairwise comparison between origin of cuttings in each substrate type in the greenhouse experiment for bacterial and fungal taxa relative abundance. Two-way ANOVAs were used to discern how substrate type (Control, Tailings or Waste rock), origin of cuttings (La Corne Mine, Westwood or Natural forest) and their interaction influenced taxa relative abundance. Additional analyses were performed for each substrate type separately and between substrate types to better assess the indivudial effects of origin and substrate type.

**Fungi**		*Pezoloma*	*Phaeosphaeriaceae_g*	*Pleosporale_f_g*	*Pleosporales _fam_Incertae_sedis_g*	*Pyrenopeziza*	*Pyrenophora*	*Russula*
	Origin	0.459	0.242	0.628	0.259	0.310	0.466	0.538
	Substrate type	< 0.001	< 0.001	< 0.001	< 0.001	< 0.001	< 0.001	< 0.001
	Interaction	0.208	0.961	0.707	0.834	0.405	0.756	0.806
**Pairwise comparison between origin**								
	Natural	2.7% A	0.4% A	0.3% A	1.6% A	0.5% A	0.4% A	2.1% A
	La Corne Mine	2.4% A	0.3% A	0.3% A	1.5% A	0.3% A	0.4% A	1.9% A
	Westwood	2.1% A	0.4% A	0.3% A	1.9% A	0.3% A	0.5% A	2.4% A
	p-value	0.856	0.372	0.653	0.490	0.533	0.643	0.910
**Pairwise comparison by substrate type**								
Control	Natural	0.0% A	0.7% A	0.6% A	3.3% A	0,0% A	1.0% A	0.0% A
	La Corne Mine	0.0% A	0.7% A	0.4% A	2.8% A	0,0% A	0.8% A	0.0% A
	Westwood	0.0% A	0.8% A	0.5% A	3.3% A	0,0% A	0.9% A	0.0% A
	p-value	0.778	0.526	0.511	0.525	0.999	0.506	0.551
Tailings	Natural	0.0% A	0.2% A	0.2% A	0.8% A	0,0% A	0.1% A	0.0% A
	La Corne Mine	0.0% A	0.2% A	0.2% A	0.9% A	0,0% A	0.2% A	0.0% A
	Westwood	0.0% A	0.3% A	0.2% A	1.0% A	0.0% A	0.3% A	0.3% A
	p-value	0.046	0.442	0.473	0.873	0.479	0.650	0.464
Waste rock	Natural	7.1% A	0.2% A	0.2% A	0.7% A	1.2% A	0.2% A	5.5% A
	La Corne Mine	7.2% A	0.2% A	0.2% A	0.9% A	0.9% A	0.3% A	5.6% A
	Westwood	5.8% A	0.2% A	0.2% A	1.3% A	0.8% A	0.3% A	6.5% A
	p-value	0.670	0.764	0.882	0.273	0.329	0.551	0.937

Supplementary Table 11. Pairwise comparison between origin of cuttings in each substrate type in the greenhouse experiment for bacterial and fungal taxa relative abundance. Two-way ANOVAs were used to discern how substrate type (Control, Tailings or Waste rock), origin of cuttings (La Corne Mine, Westwood or Natural forest) and their interaction influenced taxa relative abundance. Additional analyses were performed for each substrate type separately and between substrate types to better assess the indivudial effects of origin and substrate type.

**Fungi**		*Sebacinales_f_g*	*Sordariales_f_g*	*Sphaerosporella*	*Tomentella*	*Trichoderma*	*Vibrisseaceae_g*
	Origin	0.872	0.594	0.021	0.518	0.820	0.272
	Substrate type	< 0.001	< 0.001	< 0.001	< 0.001	0.009	< 0.001
	Interaction	0.476	0.821	0.338	0.858	0.517	0.604
**Pairwise comparison between origin**							
	Natural	0.5% A	10.8% A	3.8% B	34.1% A	1.7% A	0.2% A
	La Corne Mine	0.9% A	12.3% A	9.6% A	28.5% A	0.9% A	0.3% A
	Westwood	0.9% A	10.6% A	5.0% B	31.7% A	0.8% A	0.3% A
	p-value	0.517	0.897	0.007	0.516	0.197	0.981
**Pairwise comparison by substrate type**							
Control	Natural	0.3% A	0.1% A	6.5% A	38.8% A	0.4% A	0.3% A
	La Corne Mine	0.3% A	0.1% A	13.8% A	30.1% A	0.4% A	0.2% A
	Westwood	0.2% A	0.1% A	6.9% A	30.0% A	0.3% A	0.1% A
	p-value	0.707	0.588	0.044	0.428	0.924	0.256
Tailings	Natural	0.8% A	34.1% A	4.4% A	21.6% A	0.8% A	0.0% A
	La Corne Mine	2.1% A	32.9% A	11.4% A	18.7% A	0.6% A	0.0% A
	Westwood	1.8% A	32.5% A	5.2% A	21.0% A	0.7% A	0.0% A
	p-value	0.424	0.965	0.937	0.921	0.920	0.350
Waste rock	Natural	0.3% A	0.2% A	1.0% A	40.5% A	3.5% A	0.4% A
	La Corne Mine	0.2% A	1.3% A	3.9% A	37.6% A	1.5% A	0.6% A
	Westwood	0.7% A	0.0% A	3.2% A	43.2% A	1.3% A	0.6% A
	p-value	0.502	0.486	0.345	0.679	0.190	0.777
