# Supplementary material for: Plant Genotype Influences Physicochemical Properties of Substrate as Well as Bacterial and Fungal Assemblages in the Rhizosphere of Balsam Poplar
Source: Front Microbiol. 2020 Nov 23;11:575625. doi: 10.3389/fmicb.2020.575625 (PMC7719689; doi:10.3389/fmicb.2020.575625)
Supplement: Supplementary file 18 [file Table_12.DOCX]

# Tree production process

## Hydroponic system

Cuttings were prepared from the tree branches collected on the field. The base of each cutting was cut diagonally and immediately dipped in a rooting powder containing 0.8% of Indole-3-butyric acid, a plant hormone helping for the initiation of root development (STIM-ROOT No3, Plant Product Co. LTD). Cuttings were than inserted into a rooting medium (ROOTCUBES® 1½’ square, Smithers-OASIS) in the hydroponic system. Buds containing flowers were removed. The cuttings were watered automatically twice a day, at 08:00 and 20:00, for 4 minutes (just enough time for the container to be filled and drained slowly). Day/night greenhouse temperatures were set at 22/18°C with 16 hours supplemented lighting (less than 250 W/m2) between 08:00 and 24:00. Every four weeks of growth in hydroponic, a rooting fertilizer (8 mL / 40 L; Roots&Rhizo, Fred T. Lizer; 10-32-2, N-P-K) was added to the irrigation system to help root development.

## Growth in pots

After two months of growth in the hydroponic system, cuttings were transferred to pots and grown for another three months. The potting mix consisted of four parts peat (Agro Mix G6, Fafard), two parts vermiculite (Perlite Canada Inc.) and one part Turface (calcined clay particles, Turface MVP, Turface Athletics®). The potting mix was watered and autoclaved to kill insects potentially present in the peat. All substrates contained the same autoclaved potting mix, therefore waste mine substrates were amended with the same substrate as was contained in the control substrate, allowing for a normalized comparison of the tree substrate types. Nine grams of slow release fertilizer (18.6.8, N.P.K; Nutricote Total, Type:100, Chisso-Asahi Fertilizer Co. LTD) was then added per liter of potting mix. Cuttings were planted in 250 mL square pots. Temperature and light conditions were the same as for the hydroponic growth. For the first two months, the trees were watered automatically by a drip irrigation system twice a day for 3 minutes at 08:00 and 20:00. For the last month, the watering program was changed to three times per day for 5, 3 and 5 minutes at 08:00, 16:00 and 24:00. Trees were given extra manual watering as required.

# Greenhouse conditions for the experiment

In the greenhouse, from April to August, the trees were watered automatically using a drip irrigation system three times per day for 5 minutes at 08:00, 16:00 and 24:00. Temperature and light settings were as described previously. When transferred outside in august, the trees were watered manually, as required.

The trees were returned to the greenhouse in January as to simulate spring. The greenhouse was set to a day/night temperature of 10/5°C with 10 hours of supplemented lighting from 08:00 to 16:00 hours. The trees were immediately cut back to between 30 cm and 50 cm high so as to keep 10 buds per tree, including both lateral and terminal buds. Temperature and lighting were gradually raised by 5°C and two hours respectively at two weeks interval until reaching maximum day/night temperatures of 22/18°C and 14 hours lighting (between 06:00 and 20:00). Irrigation started one week after the first buds started to flush, again using the drip irrigation system. Trees were initially watered for 3 minutes at 08:00 once every three days. After ten days, this was increased to once every two days, then to once a day a week later, and finally to twice a day, for 5 minutes, at 08:00 and 20:00 11 days after that. The trees were grown for about three months, until they naturally set bud.
